# Synthesis of Functionalized Cannabilactones

**DOI:** 10.3390/molecules25030684

**Published:** 2020-02-06

**Authors:** Yingpeng Liu, Thanh C. Ho, Mohammed Baradwan, Maria Pascual Lopez-Alberca, Christos Iliopoulos-Tsoutsouvas, Spyros P. Nikas, Alexandros Makriyannis

**Affiliations:** 1 Center for Drug Discovery and Department of Pharmaceutical Sciences, Northeastern University, Boston, MA 02115, USA; liu.yingp@husky.neu.edu (Y.L.); t.ho@northeastern.edu (T.C.H.); baradwan.m@gmail.com (M.B.); mpascualla@gmail.com (M.P.L.-A.); iliopoulostsoutsou.c@husky.neu.edu (C.I.-T.); 2 Departments of Chemistry and Chemical Biology, Northeastern University, Boston, MA 02115, USA

**Keywords:** cannabinoids, cannabilactones, CB2 selective ligands, Suzuki cross-coupling

## Abstract

A new approach to synthesize cannabilactones using Suzuki cross-coupling reaction followed by one-step demethylation-cyclization is presented. The two key cannabilactone prototypes AM1710 and AM1714 were obtained selectively in high overall yields and in a lesser number of synthetic steps when compared to our earlier synthesis. The new approach expedited the synthesis of cannabilactone analogs with structural modifications at the four potential pharmacophoric regions.

## 1. Introduction

Two cannabinoid receptors (CB1 and CB2) belonging to the superfamily of G-protein-coupled receptors (GPCRs) are well characterized and play a vital role in multiple (patho)physiological functions [1,2]. Their widespread distribution and potential therapeutics have made CB1 and CB2 attractive targets for the treatment of pain, inflammation, glaucoma, cancer, and central nervous system (CNS) disorders [1,3,4]. The CB1 receptor is abundantly expressed in the CNS and mediates most of the cannabinoid psychotropic and behavioral effects. The CB2 receptor is predominantly expressed in immune cells and in peripheral tissues and organs. In the brain, this receptor is detectable at very low levels; however, it may be induced under inflammatory situations [5,6,7]. CB2 receptor activation does not produce the unwanted CNS mediated side effects associated with CB1, but it does have therapeutic potential in neurodegenerative, fibrotic, and inflammatory diseases [8,9]. For these reasons, the development of CB2 selective agonists has received significant attention from both pharmaceutical industry and academic institutions. The recent breakthrough work on the crystal structures of the antagonist- and agonist-bound CB1 and CB2 should aid in the future rational design of CB2 selective agonists with improved biopharmaceutical profiles [10,11,12,13,14,15].

**Figure 1:**
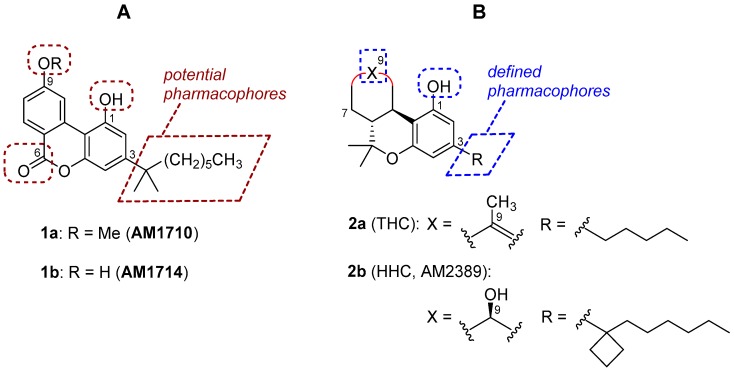
(**A**), structures of AM1710 and AM1714 and potential pharmacophores of the cannabilactone template. (**B**), representative tetrahydrocannabinol (THC) and hexahydrocannabinol (HHC) analogs with the well-defined pharmacophoric regions.

In earlier work including a preceding article of this special issue, we have identified the tricyclic cannabilactone chemical structure as a novel template for developing CB2 selective agonists [16,17]. Two first-generation cannabilactone analogs, namely AM1710 and AM1714 (**1a** and **1b**, Figure 1), exhibited potent peripheral analgesic activity in several animal models of inflammatory and neuropathic pain [16,18,19]. Additionally, AM1710 was found to suppress chemotherapy-induced neuropathic pain and delay the development of antinociceptive tolerance to morphine in rodents [20]. The cannabilactone template has some structural similarities with the psychoactive cannabis constituent (−)-Δ^9^/Δ^8^-tetrahydrocannabinol (THC, **2a**, Figure 1). Thus, both prototypes have a tricyclic ring system carrying a hydroxy group at C1 and a hydrophobic alkyl chain at C3. However, we can also observe structural dissimilarities between the two templates. In the CB2 selective cannabilactone template, the six-membered lactone contributes to the overall aromatic character, thus making the molecules a robust tricyclic system in which all three rings are coplanar [16]. In contrast, the tricyclic ring system in THC and the related hexahydrocannabinols (HHCs, for example **2b** (AM2389) [21], Figure 1) is not coplanar, and these compounds bind both at CB1 and CB2 and behave as mixed CB1/CB2 agonists [21,22,23,24]. It should also be noted that the pharmacophoric regions (Figure 1) of the classical tricyclic THC/HHC scaffold are well defined through extensive structure-activity relationship (SAR) studies [21,22]. However, the pharmacophoric regions of the cannabilactone scaffold have not been well explored. Taking together all the above, we have decided to revisit the cannabilactone prototype and explore in more detail the four potential pharmacophoric regions, including the phenolic hydroxyl at C1, the side chain at C3, the C6 keto group, and the substituents at C9 (see Figure 1). The long-term goal of this project is to develop second-generation cannabilactone analogs with optimized pharmacodynamic and pharmacokinetic properties. Toward this end, the development of a general and effective synthesis of functionalized cannabilactones was required.

## 2. Results and Discussion

**Scheme 1:**
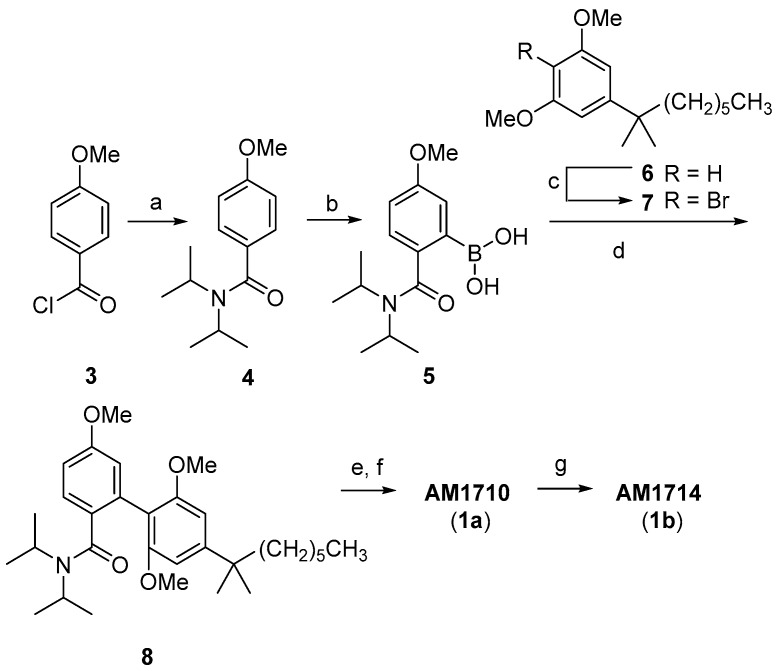
Earlier synthesis of AM1710 and AM1714 (6–7 steps, 27–38% overall yields) *^a^*. *^a^* Reagents and conditions: (a) Diisopropylamine, Et_3_N, CH_2_Cl_2_, rt, 2 h, 75% [26]; (b) i. *sec*-BuLi, *N*,*N*,*N*′,*N*′-tetramethylethylenediamine (TMEDA), THF, −78 °C, 1 h; ii. B(OCH_3_)_3_, −78 °C to rt, 12 h; iii. 5% aq. HCl, 96%; (c) Br_2_, CCl_4_, 0 °C, 20 min, 85%; (d) Pd(Ph_3_P)_4_, Ba(OH)_2_, DME/H_2_O, 110 °C, microwave irradiation, 15 min, 83%; (e) 9-iodo-9-BBN, CH_2_Cl_2_, 0 °C, 4 h; (f) AcOH, reflux, 5 h, 76% from **8**; (g) BBr_3_, CH_2_Cl_2_, reflux, 12 h, 72%.

In our earlier work [16], AM1710 and AM1714 were prepared in six to seven synthetic steps and in 38% and 27% overall yields, respectively. In this method, the Suzuki coupling reaction [25] of (2-(diisopropylcarbamoyl)-5-methoxyphenyl)boronic acid (**5**) with 2-bromo-5-(1′,1′-dimethylheptyl)-1,3-dimethoxybenzene (**7**) is the key synthetic step (Scheme 1) [16]. Both boronic acid **5** and aryl bromide **7** are not commercially available. As shown in Scheme 1, **5** was prepared from 4-methoxybenzoyl chloride (**3**) through amide formation and subsequent ortho lithiation–boronation reactions. This lengthens by two steps the overall synthesis of AM1710 and AM1714. Synthesis of the other component of the Suzuki coupling, the aryl bromide **7**, requires a careful monobromination of the synthetically feasible resorcinol dimethyl ether **6** using toxic bromine. Additionally, this earlier approach has some limitations regarding its application to the synthesis of functionalized cannabilactone analogs, especially those with modifications at C9. This is because the required boronic acids or their synthetic precursors are not commercially available, and their synthesis would require additional synthetic steps.

**Scheme 2:**
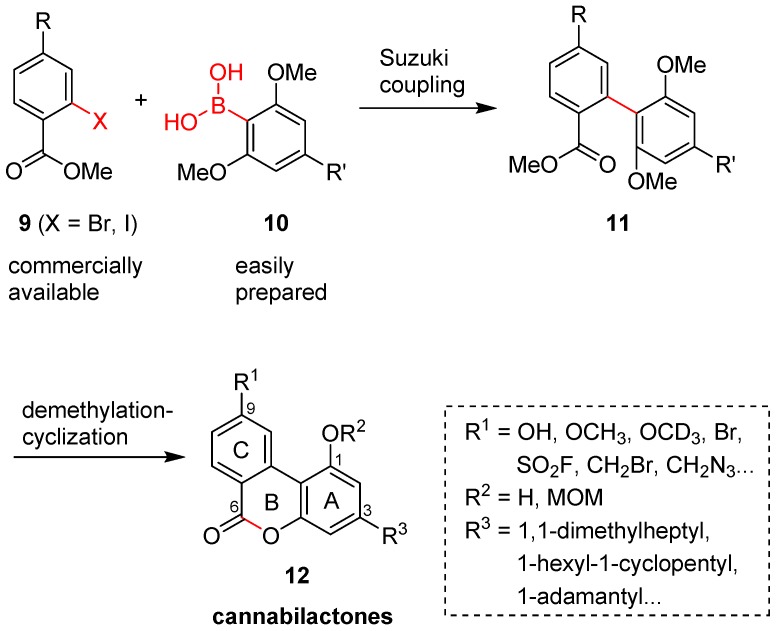
New synthesis of functionalized cannabilactones.

Herein, we report a general and effective approach for the synthesis of cannabilactones including AM1710 and AM1714 as well as their analogs with structural modifications at the potential pharmacophoric regions (Scheme 2). The new approach reverses the partners of the Suzuki cross-coupling reaction to take advantage of the large variety of commercially available aryl halides (bromides and iodides, **9**). The preparation of resorcinol boronic acids **10** is straightforward, while the use of bromine is avoided [27]. It is also worthy to note that the presence of the methyl carboxylate in the biphenyl intermediates **11** allows the subsequent demethylation–lactonization reactions to occur in a single operation and provides the final products in high overall yields. This demethylation–lactonization process could be carried out in the presence of either BBr_3_ or the milder reagent 9-iodo-9-BBN [23]. Unlike the carboxylate biphenyls **11**, the conversion of diisopropyl amide biphenyl intermediates, for example **8** (Scheme 1), to the final product lactones requires two to three synthetic steps. Due to these modifications the new synthesis of AM1710 and AM1714 proceeded in only three steps and in 55–58% overall yields. This is a significant improvement compared to the earlier method (six to seven steps, 27–38% overall yields). Furthermore, the new synthetic approach allowed us to synthesize various cannabilactone analogs with structural modifications at the C3 side chain, C9/C11, and C1 positions. Some C6 derivatives of cannabilactone were also prepared.

**Scheme 3:**
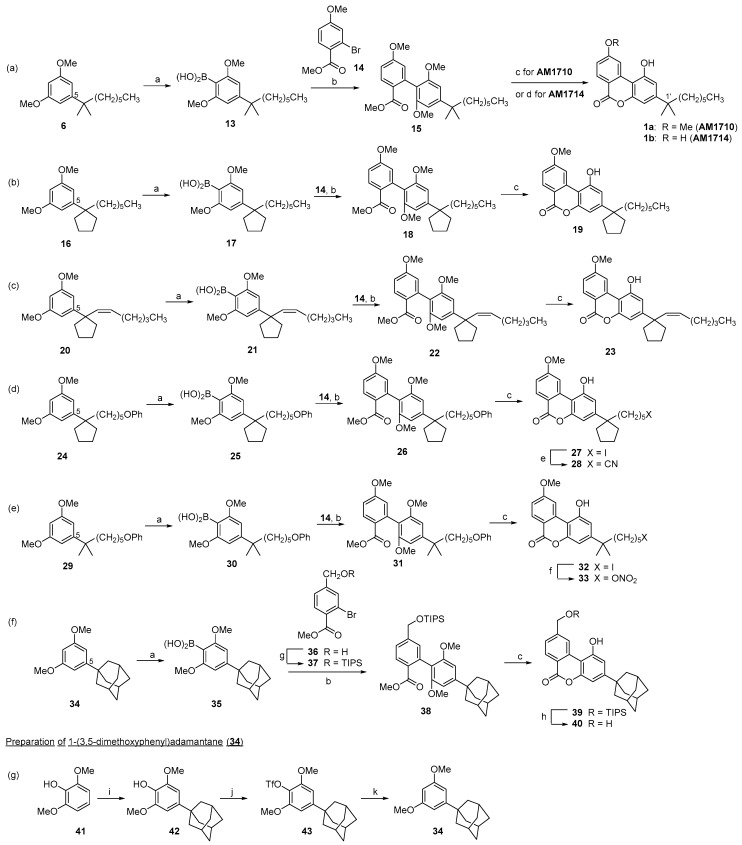
Synthesis of AM1710, AM1714, and C3 side chain modified cannabilactones (**a**–**g**) *^a^*. *^a^* Reagents and conditions: (a) i. *n*-BuLi, THF, −78 °C, 45 min, 10 °C, 1.5 h, −78 °C 30 min; ii. B(OMe)_3_, −78 °C to rt, overnight; iii. 5% aq. HCl, 88% for **13**, 65% for **17**, 80% for **21**, 73% for **25**, 61% for **30**, and 61% for **35**; (b) Cs_2_CO_3_, DME/H_2_O, Pd(Ph_3_P)_4_, 110 °C, microwave irradiation, 45 min, 84% for **15**, 69% for **18**, 81% for **22**, 76% for **26**, 84% for **31**, and 83% for **38**; (c) 9-iodo-9-BBN, CH_2_Cl_2_, 0 °C to rt, overnight, 78% for AM1710, 75% for **19**, 70% for **23**, 67% for **27**, 68% for **32**, and 73% for **39**; (d) BBr_3_, CH_2_Cl_2_, 2 days, reflux, 74% for AM1714; (e) NaCN, DMSO, rt, overnight, 90%; (f) AgNO_3_, CH_3_CN, rt, 5 h, 91%; (g) TIPSOTf, 2,6-lutidine, DCM, 0 °C to rt, 2 h, 89% (h) TBAF, THF, rt, 2 h, 90%; (i) 1-adamantanol, MeSO_3_H, 50 °C, 3 h, 58%; (j) Tf_2_NPh, Et_3_N, 4-(dimethylamino)pyridine (DMAP), CH_2_Cl_2_, reflux, 18 h, 95%; (k) PdCl_2_(Ph_3_P)_2_, dppp, *n*-Bu_3_N, HCOOH, polymethylhydrosiloxan (PMHS), DMF, reflux, 19 h, 92%.

The details of the synthesis of AM1710 and AM1714 are summarized in Scheme 3a. Cannabilactones AM1710 and AM1714 were prepared starting from commercially available 1-(1,1-dimethylheptyl)-3,5-dimethoxybenzene (**6**). Ortho-lithiation of **6** followed by in situ boronation with trimethyl borate, and hydrolysis of the resulting boronate ester intermediate led to boronic acid **13** in 88% yield. Twenty-five Suzuki cross-coupling of **13** with commercially available 2-bromo-4-methoxybenzoate (**14**) using tetrakis(triphenylphosphophine)palladium(0) as catalyst and cesium carbonate in dimethoxyethane/water under microwave irradiation at 110 °C for 45 min gave biphenyl compound **15** in 84% yield. AM1710 and AM1714 were obtained selectively in high yield from biphenyl **15**. The exposure of **15** to excess boron tribromide in dichloromethane at reflux for 2 days led to the cleavage of all methyl ether groups and lactonization in a single step that resulted in the formation of AM1714 directly in 74% yield. The use of stoichiometric 9-iodo-9-BBN at room temperature in place of boron tribromide gave AM1710 in 78% yield from **15**. During this transformation, cleavage of the methoxy group of the electron-poor aromatic C-ring bearing a carboxyester group did not occur.

Cannabilactone analogs with modifications at the C3 side chain were prepared following the general method used for the synthesis of AM1710 (Scheme 3b–f). The design of these analogs was influenced by our earlier SAR work on the C3 side chain of tricyclic tetrahydrocannabinols (THCs) and hexahydrocannabinols (HHCs) [21,23,24,28,29,30,31,32]. The addition of the larger cyclopentyl ring at C1′ was attempted to explore the effect of the size of the benzylic substituents. Incorporation of a double bond at C2′ imposes additional conformational restrictions on the flexible side chain. Groups at the terminal carbon atom will probe the potency while they enhance the polar characteristics of the molecule. The adamantyl group represents a conformationally fixed and sterically demanding side chain. For the synthesis of these cannabilactone analogs, the synthesis of resorcinol dimethyl ether precursors carrying suitable substituents at C5 is required. Thus, resorcinol dimethyl ethers **16** [24], **20** [24], **24** [21], and **29** [33] were synthesized following the procedures previously reported by our group, while 2-(3,5-dimethoxyphenyl)adamantane (**34**) was prepared from 2,6-dimethoxyphenol (**41**) as follows. Friedel–Crafts alkylation of **41** (Scheme 3g) with 1-adamantanol in the presence of methanesulfonic acid gave **42** in 58% yield. The exposure of **42** to *N*-phenyltrifluoromethanesulfonimide formed the phenolic triflate **43** in high yield (95%). Reductive cleavage of the phenolic triflate **43** in the presence of PdCl_2_, 1,3-bis(diphenylphosphino)propane (dppp), and polymethylhydrosiloxane (PHMS) gave **34** in 92% yield [31]. With resorcinol dimethyl ethers **16**, **20**, **24**, **29**, and **34** in hand, we follow the three steps used in the new synthesis of AM1710 to obtain cannabilactones **19**, **23**, **27**, **32**, and **39** in good to excellent overall yields. These steps include the formation of boronic acids from the respective resorcinol dimethyl ethers, the Suzuki coupling of boronic acids with either commercially available aryl bromide **14** or triisopropylsilyl (TIPS)-protected bromide **37**, and finally, the one-step demethylation/cyclization. Note that the phenoxy group in **26** and **31** (Scheme 3d,e) was also replaced with the iodo group upon exposure of these biphenyls to 9-iodo-9-BBN reagent. This enables the incorporation of functional groups and/or pharmaceutically favorable moieties at the terminal carbon atom of the side chain of the cannabilactone prototype. For example, the exposure of iodide **27** to sodium cyanide in dimethyl sulfoxide gave cannabilactone **28** in 90% yield, whereas the treatment of **32** with silver nitrate in acetonitrile led to **33** in 91% yield. Furthermore, silyl ether **39**, when treated with tetrabutylammonium fluoride (TBAF) in THF, formed the C3 adamantyl cannabilactone **40** in 90% yield. This analog is also an example of dual modification of the cannabilactone prototype at C3 and C11 positions.

**Scheme 4:**
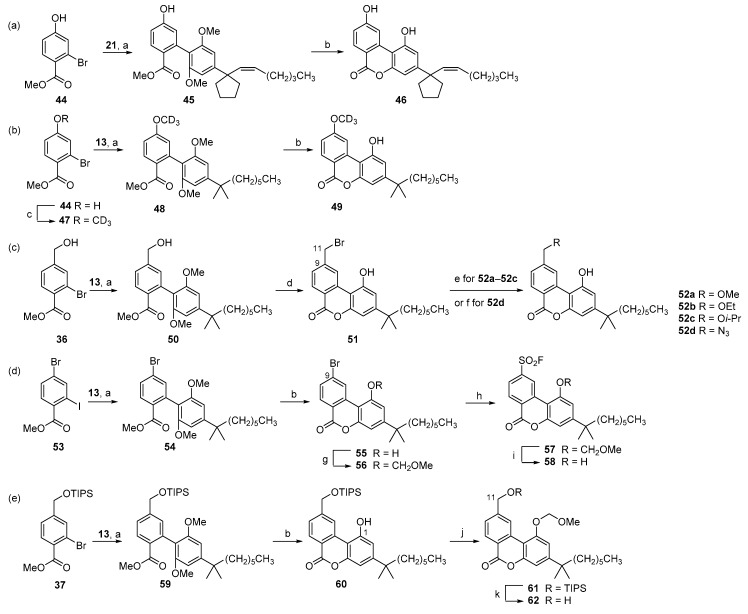
Synthesis of C9/C11 and C1 modified cannabilactones (**a**–**e**) *^a^*. *^a^* Reagents and conditions: (a) Cs_2_CO_3_, DME/H_2_O, Pd(Ph_3_P)_4_, 110 °C, microwave irradiation, 45 min, 82% for **45**, 81% for **48**, 80% for **50**, 62% for **54**, 86% for **59**; (b) 9-iodo-9-BBN, DCM, 0°C to rt, overnight, 65% for **46**, 75% for **49**, 73% for **55**, 60% for **60**; (c) K_2_CO_3_, CD_3_I, DMF, 0 °C to rt, 3 h, 86%; (d) BBr_3_, CH_2_Cl_2_, −78 °C to rt, overnight, 68%; (e) FeSO_4_·7H_2_O, CH_2_Cl_2_, MeOH, EtOH, or propan-2-ol, 130 °C, microwave, 1 h, 97–99% for **52a**–**52c**; (f) *n*-Bu_4_N^+^N_3_^−^, CH_2_Cl_2_, rt, 2 days, 96% for **52d**; (g) MOMCl, DIPEA, CH_2_Cl_2_, 0 °C to rt, 1 h, 92%; (h) DABSO, PdCl_2_(Amphos)_2_, Et_3_N, propan-2-ol, 75 °C, 24 h, then NFSI, rt, 3 h, 71%; (i) Sc(OTf)_3_, EtOH/CH_3_CN, rt, 8 h, 91%; (j) MOMCl, DIPEA, CH_2_Cl_2_, 0 °C to rt, overnight, 98%; (k) TBAF, THF, 0 °C to rt, 30 min, 97%.

Cannabilactone analogs with modifications at the C9/C11 positions were also prepared in the same manner (Scheme 4). Earlier SAR data indicate that the C9/C11 position of the tricyclic cannabinoids (i.e., THCs, HHCs) is another pharmacophore that can affect the ligand’s binding affinity for CB1 and CB2 receptors and pharmacological potency [28,30,31,34]. Thus, in this work, we incorporate various functional groups at C9 of the cannabilactone template, including the hydroxyl and deuterated methoxy groups. This is expected to probe the potential hydrogen bonding interactions between the ligand and the receptor and/or to modify the polar/pharmacokinetic profiles of the analogs [35]. Deuterium atoms are introduced to the C9 methoxy group, which is a potential labile site, in order to increase the metabolic stability. Cannabilactones bearing C11 alkoxide groups with different bulkiness (OMe, OEt, and O*i*-Pr) were prepared to explore the stereoelectronic requirements for activity of the ether group seen in AM1710. Functionalized cannabilactones carrying the electrophilic sulfonyl fluoride moiety [36], or the photoactivatable azido group [31,37] (yields nitrene upon UV irradiation) offer opportunities for developing potentially tight/irreversible binders for the receptor [2]. The synthesis of these cannabilactones requires the use of aryl halides containing an appropriate moiety that would be transformed into the desired functional group at the C9/C11 position of the final product. Aryl bromides **44**, **36**, and **53** are commercially available. Bromide **47** was prepared from **44** in good yields via *O*-alkylation, while bromide **37** was prepared through TIPS protection of **36** (Scheme 3f). Following the approach typified by the synthesis of AM1710, Suzuki cross-coupling reaction of aryl halides **44**, **47**, **36**, **53**, and **37** with either boronic acid **21** or **13**, followed by one-step demethylation/lactonization afforded cannabilactones **46**, **49**, **51**, **55**, and **60**, respectively, in satisfactory yields.

It is worthy to note that the treatment of biphenyl **50** (Scheme 4c) with boron tribromide induces multiple transformations and provides the desirable benzyl bromide **51** in a single operation and in 68% yield. These transformations involve the cleavage of all methyl ether groups, lactonization, and introduction of the C11 bromide. Additionally, the Suzuki cross-coupling reaction of methyl 4-bromo-2-iodobenzoate (**53**, Scheme 4d) proceeded with exclusive regioselectivity at the iodo carbon giving biphenyl **54** in 62% yield [38]. Cannabilactones **51**, **55**, and **60** were used as intermediates to prepare additional C9/C11 and C1 modified analogs via further functional group transformations as follows. The etherification of **51** (Scheme 4c) with methanol, ethanol, or 2-propanol in the presence of FeSO_4_·7H_2_O in a microwave reactor at 130 °C for 1 h led to the corresponding ethers **52a**–**52c** in 97–99% yields [39]. The treatment of bromide **51** with tetrabutylammonium azide in dichloromethane at room temperature provided azide **52d** in a 96% yield. The attempted sulfonylation of aryl bromide **55** (Scheme 4d) without masking the phenolic hydroxyl group did not lead to the desired product. Thus, **55** was treated with methoxymethyl chloride in the presence of *N*,*N*-diisopropylethylamine to produce **56** in 92% yield. The palladium-catalyzed sulfonylation of aryl bromide **56** using 1,4-diazabicyclo[2.2.2]octane bis(sulfur dioxide) adduct (DABSO) [40], followed by in situ treatment of the resulting sulfinate intermediate with *N*-fluorobenzene sulfonimide provided sulfonyl fluoride **57** in 71% yield [41]. This was followed by removal of the methoxymethyl protecting group with stoichiometric scandium(III) triflate in the presence of excess ethanol to give cannabilactone **58** in 91% yield [42]. Cannabilactone **60** (Scheme 4e) with a silyl ether protecting group at the C11 position can be used as an intermediate to synthesize analogs with further modifications at the C1 phenolic hydroxyl group. Such analogs would be useful to explore the role of this hydroxyl group in the cannabilactone template in terms of CB2 receptor recognition and activation. This is based on earlier observations that the C1 phenolic hydroxyl group in classical cannabinoids is important for activity, predominantly for CB1 [43,44,45,46,47]. On the other hand, compound **60** can be converted to C11-hydroxyl cannabilactone **62** in good yields via methoxymethyl ether protection and subsequent removal of the silyl ether protecting group. Compound **62** with a methoxymethyl ether protecting group at the C1 position can serve as an intermediate to prepare analogs with modifications on the C11 hydroxyl group. Thus, our approach provides a useful tool to prepare cannabilactone analogs with selective modifications on a hydroxyl group at either the C1 or C9/C11 position.

**Scheme 5:**
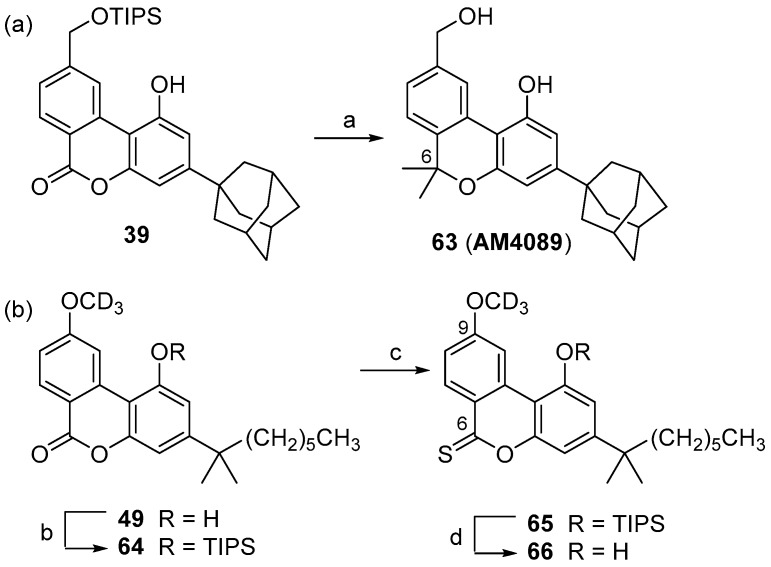
Synthesis of AM4089 and thiocannabilactone (**a**,**b**) *^a^*. *^a^* Reagents and conditions: (a) CH_3_MgBr, Et_2_O/THF, reflux, 2 h; *p*-TsOH·H_2_O, CHCl_3_, rt, 12 h, 65% from **39**; (b) TIPSOTf, 2,6-lutidine, CH_2_Cl_2_, 0 °C to rt, 2 h, 89%; (c) Lawesson reagent, toluene, reflux, 24 h, 76%; (d) TBAF, THF, −40 °C, 30 min, 82%.

The new method for the synthesis of cannabilactones also provides a short approach to prepare analogs of cannabinol (CBN), which is another understudied bioactive constituent of the cannabis plant. For example, in earlier work, we synthesized the CBN analog AM4089, which is a potent CB1 agonist, in 11 steps in ca. 1.1% overall yield [30]. Key steps in this synthesis were a Friedel–Craft alkylation reaction and an oxidative aromatization with sulfur at 250 °C. Both reactions proceeded in low yields (32% and 34%, respectively). Following the approach reported here, AM4089 was synthesized in seven steps in 12.5% overall yield (Scheme 3f and Scheme 5a). Treatment of cannabilactone **39** (Scheme 5a) with excess methylmagnesium bromide in THF at reflux [16,30] followed by cyclization and simultaneous cleavage of the silyl ether group with *p*-toluenesulfonic acid [48] afforded AM4089 (**63**) in one-pot reaction and in 65% yield from **39**.

Finally, thiocannabilactone **66** (Scheme 5b) with modifications at two potential pharmacophores, the C6 and C9, was prepared from the deuterated analog of AM1710 (**49**, Scheme 5b). Silyl ether protection of **49** gave **64** in 89% yield. Subsequent treatment of **64** with the Lawesson reagent [49,50] in toluene at reflux afforded thioester **65** in 76% yield. This was followed by fluorodesilylation using tetrabutylammonium fluoride to provide **66** in 82% yield.

## 3. Experimental Section

All reagents and solvents were purchased from Sigma-Aldrich Chemical Company, unless otherwise specified, and used without further purification. All anhydrous reactions were performed under a static argon atmosphere in flame-dried glassware using scrupulously dry solvents. Flash column chromatography employed silica gel 60 (230–400 mesh). All compounds were demonstrated to be homogeneous by analytical thin layer chromatography (TLC) on pre-coated silica gel TLC plates (Merck, 60 F245 on glass, layer thickness 250 mm), and chromatograms were visualized by phosphomolybdic acid staining. IR spectra were recorded on a PerkinElmer Spectrum One FT-IR spectrometer. NMR spectra were recorded in the indicated solvent on Varian 500 (^1^H-NMR at 500 MHz, ^13^C-NMR at 126 MHz), and Bruker 400 (^1^H-NMR at 400 MHz, ^13^C-NMR at 100 MHz) NMR spectrometers, and chemical shifts are reported in units of δ relative to internal tetramethylsilane (TMS). Multiplicities are indicated as br (broadened), s (singlet), d (doublet), t (triplet), q (quartet), m (multiplet), and coupling constants (*J*) and are reported in hertz (Hz). Mass spectral data are reported in the form of *m*/*z* (intensity relative to base = 100). Purities of compounds were determined by LC/MS analysis using a Waters MicroMass ZQ system (electrospray ionization (ESI) with a Waters-2525 binary gradient module coupled to a photodiode array detector (Waters-2996) and evaporative light scattering (ELS) detector (Waters-2424) using a XTerra MS C18 (5 µm, 4.6 mm × 50 mm column and acetonitrile/water) and were >95%.

*Methyl 2′,5,6′-trimethoxy-4′-(2-methyloctan-2-yl)-[1,1′-biphenyl]-2-carboxylate* (**15**). Argon was bubbled through a mixture of boronic acid **13** [27] (1.43 g, 4.65 mmol), methyl 2-bromo-4-methoxybenzoate (**14**, 950 mg, 3.88 mmol), and Cs_2_CO_3_ (5.06 g, 15.5 mmol) in DME/H_2_O (5:1, 10.5 mL of DME and 2.1 mL of H_2_O) for 10 min. The Pd(PPh_3_)_4_ (448 mg, 0.39 mmol) catalyst was added to the mixture while argon bubbling was maintained through the mixture, and degassing was continued for an additional 5 min. The reaction mixture was microwaved with stirring at 110 °C for 45 min in a Biotage apparatus [25]. Then, the mixture was cooled to room temperature and filtered through a short celite pad. Volatiles were removed under reduced pressure, and Et_2_O was added. The ethereal solution was washed with water and brine, dried over MgSO_4_, and concentrated under reduced pressure. Purification by flash column chromatography on silica gel (10–30% EtOAc/hexane) gave **15** (1.4 g, 84% yield) as a yellow oil. IR (thin film, cm^−1^) 2930, 1727 (CO), 1600, 1575, 1282, 1256, 1238, 1121, 1032, 831, 781, 671; ^1^H-NMR (500 MHz, CDCl_3_) δ 7.93 (d, *J* = 8.8 Hz, 1H, Ar-H), 6.86 (dd, *J* = 8.8, 1.5 Hz, 1H, Ar-H), 6.82 (d, *J* = 1.5 Hz, 1H, Ar-H), 6.56 (s, 2H, Ar-H), 3.82 (s, 3H, −OCH_3_), 3.68 (s, 6H, −OCH_3_), 3.54 (s, 3H, −COOCH_3_), 1.65–1.57 (m, 2H), 1.32 (s, 6H, >C(CH_3_)_2_), 1.24 (m, 6H), 1.11 (m, 2H), 0.84 (t, *J* = 6.7 Hz, 3H). ^13^C-NMR (100 MHz, CDCl_3_) δ 167.9, 161.7, 156.6, 150.9, 137.5, 131.8, 124.1, 117.7, 116.7, 112.3, 102.2, 55.9, 55.3, 51.2, 44.7, 38.2, 31.8, 30.0, 28.9, 24.7, 22.6, 14.1. Mass spectrum (ESI) *m*/*z* (relative intensity) 451 (M^+^ + Na, 30), 397 (M^+^ + H − MeOH, 100). LC/MS analysis (Waters MicroMass ZQ system) showed retention time 5.9 min for the title compound.

*1-Hydroxy-9-methoxy-3-(2-methyloctan-2-yl)-6H-benzo[c]chromen-6-one* (**1a**). To a solution of **15** (100 mg, 0.23 mmol) in anhydrous CH_2_Cl_2_ (3.0 mL) at 0 °C was added dropwise 9-Iodo-9-borabicyclo[3.3.1]nonane (9-iodo-9-BBN, 0.78 mL, 1.0 M solution in hexane, 0.78 mmol). The reaction mixture was stirred at 0 °C for 1 h; then, it was warmed to room temperature over a 3-h period and kept stirring at room temperature overnight. Then, the mixture was treated with 0.5 mL of ethanolamine and stirred for an additional 30 min. Solvent evaporation and purification by flash column chromatography on silica gel (CH_2_Cl_2_/Et_2_O/hexane, 2:2:6) gave **1a** (67 mg, 78%) as a white solid. m.p. 149–151 °C. Literature [16], m.p. 149–151 °C. IR (thin film, cm^−1^) 3210 (OH) 1676 (CO), 1606, 1401, 1302, 1229, 1110; ^1^H NMR (500 MHz, CDCl_3_) δ 8.49 (d, *J* = 2.5 Hz, 1H, Ar-H), δ 8.35 (d, *J* = 8.9 Hz, 1H, Ar-H), 7.07 (dd, *J* = 8.9, 2.5 Hz, 1H, Ar-H), 6.96 (d, *J* = 1.8 Hz, 1H, Ar-H), 6.67 (d, *J* = 1.8 Hz, 1H, Ar-H), 5.66 (brs, 1H, OH), 3.97 (s, 3H, OCH_3_), 1.60–1.57 (m, 2H, CH_2_), 1.29 (s, 6H, 2 × CH_3_), 1.25–1.18 (m, 6H, 3 × CH_2_), 1.08–1.03 (m, 2H, CH_2_), 0.84 (t, *J* = 6.7 Hz, 3H, CH_3_). HRMS (ESI) for C_23_H_29_O_4_: calculated 369.2066; found 369.2056. Mass spectrum (ESI) *m*/*z* (relative intensity) 369 (M^+^ + H, 100). LC/MS analysis (Waters MicroMass ZQ system) showed retention time 5.5 min for the title compound.

*1,9-Dihydroxy-3-(2-methyloctan-2-yl)-6H-benzo[c]chromen-6-one* (**1b**). To a solution of **15** (43 mg, 0.1 mmol) in CH_2_Cl_2_ (3.0 mL) under an argon atmosphere at room temperature, 1 M solution of BBr_3_ in CH_2_Cl_2_ (1.2 mL, 1.2 mmol) was added, and the reaction mixture was refluxed for 2 days. After the mixture was cooled to room temperature, water was added slowly, and the organic material was extracted with CH_2_Cl_2_. The combined organic extracts were washed with 5% aqueous NaHCO_3_, water, and brine, dried over MgSO_4_, filtered, and concentrated under reduced pressure. The residue was purified by silica gel column chromatography with EtOAc/hexane (3/7) as eluent to afford **1b** (26 mg, 74%) as an off-white foam. m.p. 104–108 °C. Literature [16], m.p. 103–108 °C. IR (thin film, cm^−1^) 3319 (OH), 2929, 1687 (CO), 1622, 1600, 1410, 1277, 1217, 1108, 1057, 748; ^1^H-NMR (400 MHz, MeOD-*d_4_*) δ 8.58 (d, *J* = 2.4 Hz, 1H, Ar-H), 8.17 (d, *J* = 8.7 Hz, 1H, Ar-H), 8.94 (dd, *J* = 8.7, 2.4 Hz, 1H, Ar-H), 6.82 (d, *J* = 1.7 Hz, 1H, Ar-H), 6.77 (d, *J* = 1.7 Hz, 1H, Ar-H), 1.65–1.55 (m, 2H), 1.28 (s, 6H), 1.25–1.14 (m, 6H), 1.13–1.01 (m, 2H), 0.82 (t, *J* = 6.8 Hz, 3H); ^13^C-NMR (100 MHz, MeOD-*d*_4_) δ 165.1, 163.9, 157.7, 154.1, 154.0, 138.9, 133.2, 116.9, 113.8, 113.0, 110.7, 106.9, 105.6, 45.3, 38.8, 32.9, 31.0, 29.1, 25.8, 23.6, 14.4. HRMS (ESI) for C_22_H_27_O_4_: calculated 355.1919; found 355.1912. Mass spectrum (ESI) *m*/*z* (relative intensity) 355 (M^+^ + H, 100). LC/MS analysis (Waters MicroMass ZQ system) showed retention time 5.2 min for the title compound.

The experimental procedures for the synthesis and characterization data of novel compounds, the novel experimental procedures for the synthesis and characterization data of reported compounds, and the reproductions of NMR spectra for representative and final compounds can be found at the supplementary materials.

## 4. Conclusions

Following our earlier work including a preceding article of this special issue, an efficient synthesis of cannabilactones has been achieved via Suzuki cross-coupling reaction followed by one-step demethylation/cyclization. The two key cannabilactone prototypes AM1710 and AM1714 were obtained selectively in high overall yields and in a lesser number of synthetic steps when compared to our earlier synthesis. The new synthetic approach was used to prepare cannabilactone analogs and derivatives with modifications at four potential pharmacophoric regions including the C3 side chain, as well as the C9/C11, C1, and C6 positions.

## Supplementary Materials

The following are available online, (i) experimental procedures for the synthesis and characterization data of novel compounds **17**, **18**, **19**, **21**, **22**, **23**, **25**, **26**, **27**, **28**, **30**, **31**, **32**, **33**, **35**, **37**, **38**, **39**, **43**, **45**, **46**, **47**, **48**, **49**, **50**, **51**, **52a–d**, **54**, **55**, **56**, **57**, **58**, **59**, **60**, **61**, **62**, **64**, **65**, and **66**; (ii) novel experimental procedures for the synthesis and characterization data of reported compounds **34**, **40**, **42**, and **63**; (iii) reproductions of NMR spectra for representative and final compounds **1a** (AM1710), **1b** (AM1714), **19**, **23**, **27**, **28**, **32**, **33**, **39**, **40**, **46**, **49**, **51**, **52a–d**, **54**, **55**, **56**, **57**, **58**, **60**, **62**, **63**, and **66**.
