# Supplementary material for: Synthesis of Functionalized Cannabilactones
Source: Molecules. 2020 Feb 6;25(3):684. doi: 10.3390/molecules25030684 (PMC7037859; doi:10.3390/molecules25030684)
Supplement: Supplementary file 1 [file molecules-25-00684-s001.pdf]

SUPPORTING INFORMATION

Synthesis of functionalized cannabilactones

Yingpeng Liu,[a] Thanh C. Ho,[a] Mohammed Baradwan,[a] Maria Pascual Lopez-Alberca,[a] Christos Iliopoulos-Tsoutsouvas,[a] Spyros P. Nikas,*[,a] Alexandros Makriyannis.*[,a,b]

[a]Center for Drug Discovery and Department of Pharmaceutical Sciences, Northeastern University, Boston, Massachusetts 02115, United States

[b]Departments of Chemistry and Chemical Biology, Northeastern University, Boston, Massachusetts 02115, United States

*To whom correspondence should be addressed.

LIST OF CONTENTS

1. Experimental procedures, spectroscopic, analytical, and physical data for

all synthesized compounds....…………………………………………...……….………..... Pages 2−21

2. References…....…………….........………………………………………………...….. Page 22

3. Reproductions of the NMR spectra for final compounds……………………………………....Page 23

Experimental section

All reagents and solvents were purchased from Sigma-Aldrich Chemical Company, unless otherwise specified, and used without further purification. All anhydrous reactions were performed under a static argon atmosphere in flame-dried glassware using scrupulously dry solvents. Flash column chromatography employed silica gel 60 (230-400 mesh). All compounds were demonstrated to be homogeneous by analytical TLC on pre-coated silica gel TLC plates (Merck, 60 F245 on glass, layer thickness 250 mm), and chromatograms were visualized by phosphomolybdic acid staining. IR spectra were recorded on a PerkinElmer Spectrum One FT-IR spectrometer. NMR spectra were recorded in the indicated solvent on Varian 500 (^{1}H at 500 MHz, ^{13}C at 126 MHz), and Bruker 400 (^{1}H at 400 MHz, ^{13}C at 100 MHz) NMR spectrometers and chemical shifts are reported in units of δ relative to internal TMS. Multiplicities are indicated as br (broadened), s (singlet), d (doublet), t (triplet), q (quartet), m (multiplet) and coupling constants (*J*) are reported in hertz (Hz). Mass spectral data are reported in the form of m/z (intensity relative to base = 100). Purities of compounds were determined by LC/MS analysis using a Waters MicroMass ZQ system (electrospray ionization (ESI) with Waters-2525 binary gradient module coupled to a photodiode array detector (Waters-2996) and ELS detector (Waters-2424) using a XTerra MS C18 (5 µm, 4.6 mm × 50 mm column and acetonitrile/water) and were > 95 %.

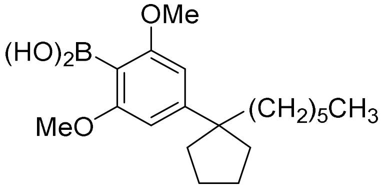

(4-(1-Hexylcyclopentyl)-2,6-dimethoxyphenyl)boronic acid (17). To a solution of 1-(1-hexylcyclopentyl)-3,5-dimethoxybenzene[1] (**16**, 206 mg, 0.71 mmol) in anhydrous THF (1.8 mL) at −78 °C under an argon atmosphere was added dropwise *n*-BuLi (0.57 mL of a 2.5 M solution in hexane, 1.42 mmol). The reaction mixture was stirred at −78 °C for 45 min, then at 10 °C for 1.5h, and back to −78 °C for another 30 min. To the reaction mixture was added dropwise trimethyl borate (369 mg, 3.55 mmol) and it was stirred from −78 °C to room temperature over a period of 12 h. Then the reaction mixture was cooled to 0 °C and acidified to pH 6.5 with 5% aqueous HCl, and extracted with Et_2O. The combined organic extracts were washed with brine, dried over $MgSO_4$ and concentrated under reduced pressure. Purification by flash column chromatography on silica gel (10-30% EtOAc/hexane) gave **17** (153 mg, 65 % yield) as a colorless viscous oil. IR (thin film, cm^{-1}) 3508 (OH), 2924, 2858, 1607, 1559, 1421, 1118, 764, 704; ^{1}H NMR (500 MHz, $CDCl_3$) δ 7.19 (s, 2H, 2 × OH), 6.52 (s, 2H, Ar-H), 3.90 (s, 6H, $-OCH_3$), 1.93 – 1.79 (m, 4H), 1.78 – 1.61 (m, 4H), 1.60 – 1.53 (m, 2H), 1.24 – 1.09 (m, 6H), 1.03 – 0.91 (m, 2H), 0.83 (t, *J* = 7.0 Hz, 3H). ^{13}C NMR (100 MHz, $CDCl_3$) δ 165.1, 155.4, 103.3, 55.9, 51.9, 41.7, 37.6, 31.7, 29.8, 25.1, 23.2, 22.6, 14.0.

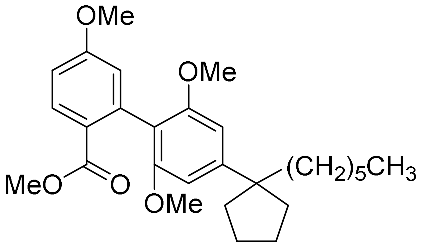

Methyl 4'-(1-hexylcyclopentyl)-2',5,6'-trimethoxy-[1,1'-biphenyl]-2-carboxylate (18). The synthesis was carried out as described for **15**, using boronic acid **17** (150 mg, 0.45 mmol), **14** (121 mg, 0.49 mmol), Cs_2CO_3 (587 mg, 1.8 mmol) and $Pd(PPh_3)_4$ (52 mg, 0.045 mmol) in DME/H_2O (5:1, 1.25 mL of DME and 0.25 mL of H_2O). The reaction was completed in 45 min and the crude oil obtained after work up was purified by flash column chromatography on silica gel (10-25% EtOAc/hexane) to give **18** (140 mg, 69 % yield) as a yellow oil. IR (thin film, cm^{-1}) 2953, 2933, 1733 (CO), 1581, 1512, 1322, 1236, 1181, 1019, 863, 757, 696; ^1H NMR (500 MHz, $CDCl_3$) δ 7.94 (d, J = 8.7 Hz, 1H, Ar-H), 6.88 (dd, J = 8.7, 2.5 Hz, 1H, Ar-H), 6.84 (d, J = 2.5 Hz, 1H, Ar-H), 6.52 (s, 2H, Ar-H), 3.83 (s, 3H, -OCH_3), 3.69 (s, 6H, -OCH_3), 3.53 (s, 3H, - $COOCH_3$), 2.00 – 1.90 (m, 2H), 1.89 – 1.81 (m, 2H), 1.80 – 1.65 (m, 4H), 1.63 – 1.56 (m, 2H), 1.24 – 1.14 (m, 6H), 1.09 – 1.00 (m, 2H), 0.84 (t, J = 7.0 Hz, 3H). ^{13}C NMR (100 MHz, $CDCl_3$) δ 168.0, 161.6, 156.4, 150.3, 137.4, 131.9, 124.2, 117.7, 116.6, 112.3, 103.2, 55.9, 55.3, 51.6, 51.1, 42.0, 37.8, 31.8, 29.9, 25.2, 23.4, 22.6, 14.0.

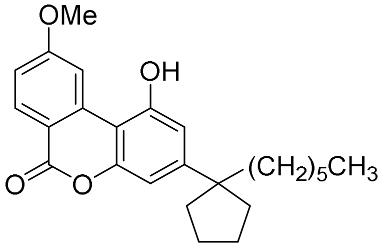

3-(1-Hexylcyclopentyl)-1-hydroxy-9-methoxy-6H-benzo[c]chromen-6-one (19). The synthesis was carried out as described for **1a**, using **18** (140 mg, 0.31 mmol) and 9-iodo-9-BBN (1.0 mL of a 1.0 M in hexane, 1.0 mmol) in anhydrous CH_2Cl_2 (3.8 mL). The reaction was quenched by ethanolamine (0.5 mL) and the crude obtained after work up was purified by flash column chromatography on silica gel (CH_2Cl_2/Et_2O/hexane, 2:2:6) to give **19** (91 mg, 75 % yield) as a white solid. Mp 159-164 °C. IR (thin film, cm^{-1}) 3321 (OH), 2926, 1683 (CO), 1609, 1403, 1106, 1030, 872, 747; ^1H NMR (500 MHz, $CDCl_3$) δ 8.56 (d, J = 2.4 Hz, 1H, Ar-H), 8.36 (d, J = 8.8 Hz, 1H, Ar-H), 7.06 (dd, J = 8.8, 2.4 Hz, 1H, Ar-H), 6.88 (d, J = 1.5 Hz, 1H, Ar-H), 6.71 (d, J = 1.5 Hz, 1H, Ar-H), 6.48 (s, 1H, OH), 3.96 (s, 3H, -OCH_3), 1.95 – 1.83 (m, 2H), 1.82 – 1.73 (m, 2H), 1.73 – 1.59 (m, 4H), 1.59 – 1.52 (m, 2H), 1.22 – 1.07 (m, 6H), 0.97 (s, 2H), 0.80 (t, J = 7.1 Hz, 3H). ^{13}C NMR (100 MHz, $CDCl_3$) δ 164.6, 161.5, 153.6, 152.8, 152.4, 136.7, 132.3, 114.9, 113.7, 110.8, 110.7, 109.0, 104.8, 55.6, 51.2, 41.8, 37.6, 31.7, 29.9, 25.3, 23.2, 22.6, 14.0. HRMS (ESI) for $C_{25}H_{31}O_4$: calculated 395.2222; found 395.2214. Mass spectrum (ESI) m/z (relative intensity) 395 (M^+ + H, 100). LC/MS analysis (Waters MicroMass ZQ system) showed retention time 5.8 min for the title compound.

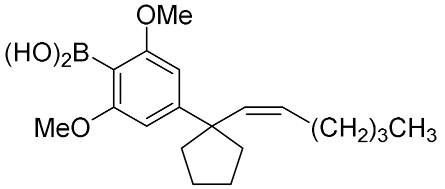

(Z)-(4-(1-(Hex-1-en-1-yl)cyclopentyl)-2,6-dimethoxyphenyl)boronic acid (21). The synthesis was carried out as described for **17**, using **20**[1] (286 mg, 1.0 mmol), *n*-BuLi (0.8 mL of a 2.5 M solution in hexane, 2.0 mmol) and trimethyl borate (520 mg, 5.0 mmol) in dry THF (2.5 mL). The crude oil obtained after acidic work up was purified by flash column chromatography on silica gel (10-30% EtOAc/hexane) to give **21** (265 mg, 80 % yield) as a colorless viscous oil. IR (thin film, cm^{-1}) 3513 (OH), 3087, 2941, 2869, 1606, 1587, 1554, 1351, 1171, 1113, 761; ^1H NMR (500 MHz, $CDCl_3$) δ 7.18 (s, 2H, 2 × OH), 6.64 (s, 2H, Ar-H), 5.71 (d, J = 11.3 Hz, 1H), 5.34 (dt, J = 11.3, 7.4 Hz, 1H), 3.89 (s, 6H, -OCH_3), 1.98 (m, 4H), 1.84 – 1.65 (m, 6H), 1.44 – 1.05 (m, 4H), 0.74 (t, J = 6.9 Hz, 3H). ^{13}C NMR (100 MHz, $CDCl_3$) δ 165.1, 155.5, 138.0, 132.7, 103.3, 55.9, 52.8, 41.1, 31.3, 28.4, 23.7, 22.3, 13.9.

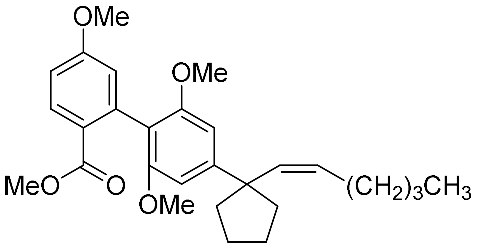

Methyl (Z)-4'-(1-(hex-1-en-1-yl)cyclopentyl)-2',5,6'-trimethoxy-[1,1'-biphenyl]-2-carboxylate (22). The synthesis was carried out as described for **15**, using boronic acid **21** (265 mg, 0.79 mmol), **14** (197 mg, 0.80 mmol), Cs_2CO_3 (1043 mg, 3.20 mmol) and $Pd(PPh_3)_4$ (93 mg, 0.08 mmol) in DME/H_2O (5:1, 2.5 mL of DME and 0.5 mL of H_2O). The reaction was completed in 45 min and the crude oil obtained after work up was purified by flash column chromatography on silica gel (5-20% EtOAc/hexane) to give **22** (290 mg, 81 % yield) as a yellow oil. IR (thin film, cm^{-1}) 3085, 2974, 2911, 2854, 1722 (CO), 1575, 1433, 1122, 1021, 773, 696; ^{1}H NMR (500 MHz, $CDCl_3$) δ 7.94 (d, *J* = 8.7 Hz, 1H, Ar-H), 6.87 (dd, *J* = 8.7, 2.6 Hz, 1H, Ar-H), 6.81 (d, *J* = 2.6 Hz, 1H, Ar-H), 6.63 (s, 2H, Ar-H), 5.74 (d, *J* = 11.3 Hz, 1H), 5.34 (dt, *J* = 11.3, 7.4 Hz, 1H), 3.83 (s, 3H, $-OCH_3$), 3.67 (s, 6H, $-OCH_3$), 3.54 (s, 3H, $-COOCH_3$), 2.12 – 2.03 (m, 2H), 2.03 – 1.95 (m, 2H), 1.87 – 1.71 (m, 6H), 1.19 – 1.45 (m, 4H), 0.80 (t, *J* = 7.0 Hz, 3H). ^{13}C NMR (100 MHz, $CDCl_3$) δ 167.9, 161.6, 156.4, 150.3, 138.7, 137.4, 132.0, 131.8, 124.1, 117.8, 116.6, 112.2, 103.2, 55.9, 55.3, 52.7, 51.1, 41.1, 31.5, 28.3, 23.7, 22.4, 13.9. Mass spectrum (ESI) m/z (relative intensity) 475 (M^+ + Na, 30), 421 (M^+ -MeO, 100). LC/MS analysis (Waters MicroMass ZQ system) showed retention time 5.9 min for the title compound.

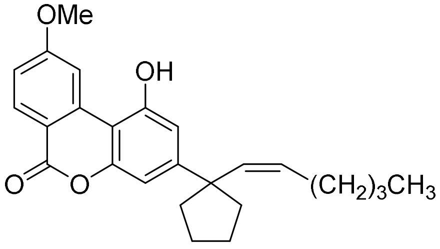

(Z)-3-(1-(Hex-1-en-1-yl)cyclopentyl)-1-hydroxy-9-methoxy-6H-benzo[c]chromen-6-one (23). The synthesis was carried out as described for **1a**, using **22** (110 mg, 0.24 mmol), 9-iodo-9-BBN (0.8 mL of a 1.0 M in hexane, 0.80 mmol) in anhydrous CH_2Cl_2 (3 mL). The reaction was quenched by ethanolamine (0.5 mL) and the crude obtained after work up was purified by flash column chromatography on silica gel (CH_2Cl_2/Et_2O/hexane, 2:2:6) to give **23** (67 mg, 70 % yield) as a white solid. Mp 146-149 °C. IR (thin film, cm^{-1}) 3269 (OH), 2955, 2871, 1679 (CO), 1604, 1399, 1103, 1027, 748; ^{1}H NMR (500 MHz, $CDCl_3$) δ 8.50 (d, *J* = 2.3 Hz, 1H, Ar-H), 8.35 (d, *J* = 8.8 Hz, 1H, Ar-H), 7.06 (dd, *J* = 8.8, 2.3 Hz, 1H, Ar-H), 7.00 (d, *J* = 1.4 Hz, 1H, Ar-H), 6.72 (d, *J* = 1.4 Hz, 1H, Ar-H), 5.88 (s, 1H, OH), 5.71 (d, *J* = 11.3 Hz, 1H), 5.34 (dt, *J* = 11.3, 7.4 Hz, 1H), 3.97 (s, 3H, $-OCH_3$), 2.05-1.90 (m, 4H), 1.81 – 1.66 (m, 6H), 1.14-1.06 (m, 4H), 0.71 (t, *J* = 6.9 Hz, 3H). ^{13}C NMR (100 MHz, $CDCl_3$) δ 164.6, 161.6, 153.7, 152.8, 152.6, 137.8, 136.7, 132.9, 132.3, 114.9, 113.6, 111.0, 110.7, 108.6, 104.7, 55.6, 52.0, 41.0, 31.2, 28.5, 23.6, 22.3, 13.8. HRMS (ESI) for $C_{25}H_{29}O_4$: calculated 393.2066; found 393.2062. Mass spectrum (ESI) m/z (relative intensity) 393 (M^+ + H, 100). LC/MS analysis (Waters MicroMass ZQ system) showed retention time 5.6 min for the title compound.

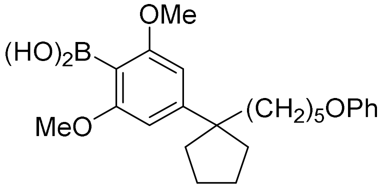

(2,6-Dimethoxy-4-(1-(5-phenoxypentyl)cyclopentyl)phenyl)boronic acid (25). The synthesis was carried out as described for **17**, using **24**[2] (1050 mg, 2.85 mmol), *n*-BuLi (2.3 mL of a 2.5 M solution in hexane, 5.7 mmol) and trimethyl borate (1481 mg, 14.25 mmol) in dry THF (7.1 mL). The crude oil obtained after acidic work up was purified by flash column chromatography on silica gel (10-30% EtOAc/hexane) to give **25** (853 mg, 73 % yield) as a colorless viscous oil. IR (thin film, cm^{-1}) 3515 (OH), 2938, 2868, 1604, 1557, 1328, 1228, 1108, 753, 690; ^{1}H NMR (500 MHz, $CDCl_3$) δ 7.26 (t, *J* = 7.9 Hz, 2H, Ar-H), 7.19 (s, 2H, 2 x OH), 6.92 (t, *J* = 7.9 Hz, 1H, Ar-H), 6.84 (d, *J* = 7.9 Hz, 2H, Ar-H), 6.53 (s, 2H, Ar-H), 3.90 (s, 6H, $-OCH_3$), 3.86 (t, *J* = 6.4 Hz, 2H), 1.94 – 1.81 (m, 4H), 1.79 – 1.64 (m, 6H), 1.64 – 1.56 (m, 2H), 1.39 – 1.29 (m, 2H), 1.11 – 1.01 (m, 2H). ^{13}C NMR (100 MHz, $CDCl_3$) δ 165.2, 159.0, 155.2, 129.3, 120.5, 114.5, 103.3, 67.7, 55.9, 51.9, 41.6, 37.6, 29.0, 26.6, 24.9, 23.2.

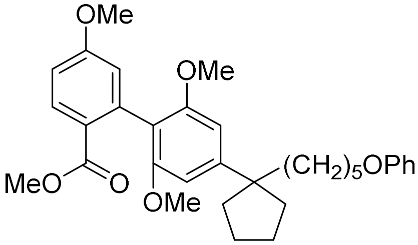

Methyl 2',5,6'-trimethoxy-4'-(1-(5-phenoxypentyl)cyclopentyl)-[1,1'-biphenyl]-2-carboxylate (26). The synthesis was carried out as described for **15**, using boronic acid **25** (770 mg, 1.87 mmol), **14** (458 mg, 1.87 mmol), Cs_2CO_3 (2.44 g, 7.48 mmol) and $Pd(PPh_3)_4$ (219 mg, 0.19 mmol) in DME/H_2O (5:1, 5 mL of DME and 1 mL of H_2O). The reaction was completed in 45 min and the crude oil obtained after work up was purified by flash column chromatography on silica gel (10-40% EtOAc/hexane) to give **26** (757 mg, 76 % yield) as a yellow oil. IR (thin film, cm^{-1}) 2937, 2863, 1728 (CO), 1600, 1574, 1237, 1121, 1019, 754, 692; ^{1}H NMR (500 MHz, $CDCl_3$) δ 7.94 (d, *J* = 8.7 Hz, 1H, Ar-H), 7.25 (t, *J* = 8.0 Hz, 2H, Ar-H), 6.94 – 6.82 (m, 5H, Ar-H), 6.52 (s, 2H, Ar-H), 3.89 (t, *J* = 6.5 Hz, 2H), 3.83 (s, 3H, $-OCH_3$), 3.68 (s, 6H, $-OCH_3$), 3.53 (s, 3H, $-COOCH_3$), 2.02 – 1.93 (m, 2H), 1.91 – 1.82 (m, 2H), 1.79 – 1.67 (m, 6H), 1.66 – 1.61 (m, 2H), 1.42 – 1.33 (m, 2H), 1.19 – 1.09 (m, 2H). ^{13}C NMR (100 MHz, $CDCl_3$) δ 167.8, 161.7, 159.1, 156.5, 150.1, 137.5, 131.8, 129.3, 124.1, 120.4, 117.7, 116.9, 114.5, 112.3, 103.3, 67.8, 55.9, 55.3, 51.6, 51.1, 41.9, 37.8, 29.2, 26.7, 25.1, 23.4. Mass spectrum (ESI) m/z (relative intensity) 555 (M^+ + Na, 30), 501 (M^+ -MeO, 100). LC/MS analysis (Waters MicroMass ZQ system) showed retention time 6.0 min for the title compound.

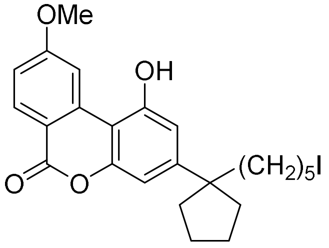

1-Hydroxy-3-(1-(5-iodopentyl)cyclopentyl)-9-methoxy-6H-benzo[c]chromen-6-one (27). The synthesis was carried out as described for **1a**, using **26** (620 mg, 1.16 mmol), 9-iodo-9-BBN (5.3 mL of a 1.0 M in hexane, 5.3 mmol) in anhydrous CH_2Cl_2 (14.5 mL). The reaction was quenched by ethanolamine (1 mL) and the crude

obtained after work up was purified by flash column chromatography on silica gel (CH_2Cl_2/Et_2O/hexane, 2:2:6) to give **27** (395 mg, 67 % yield) as a white solid. Mp 141-143 ºC. IR (thin film, cm^{-1}) 3280 (OH), 2925, 2854, 1694 (CO), 1673, 1608, 1445, 1401, 1298, 1228, 1104, 869, 748; ^{1}H NMR (500 MHz, $CDCl_3$) δ 8.53 (d, J = 2.4 Hz, 1H, Ar-H), 8.36 (d, J = 8.8 Hz, 1H, Ar-H), 7.07 (dd, J = 8.8, 2.4 Hz, 1H, Ar-H), 6.88 (d, J = 1.5 Hz, 1H, Ar-H), 6.67 (d, J = 1.5 Hz, 1H, Ar-H), 6.17 (s, 1H, OH), 3.97 (s, 3H, -OCH_3), 3.08 (t, J = 7.0 Hz, 2H), 1.95 – 1.87 (m, 2H), 1.83 –1.54 (m, 10H), 1.30 – 1.18 (m, 2H), 1.09 – 0.95 (m, 2H). ^{13}C NMR (100 MHz, $CDCl_3$) δ 164.7, 161.6, 153.9, 152.8, 152.0, 136.7, 132.3, 115.0, 113.6, 110.8, 110.7, 108.9, 104.9, 55.6, 51.1, 41.6, 37.6, 33.3, 31.0, 24.3, 23.2, 7.1. Mass spectrum (ESI) m/z (relative intensity) 507 (M^+ + H, 100). LC/MS analysis (Waters MicroMass ZQ system) showed retention time 5.7 min for the title compound.

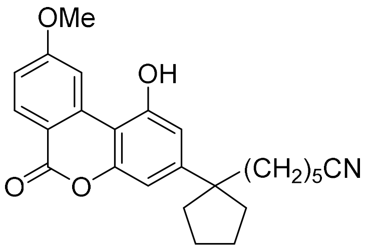

6-(1-(1-Hydroxy-9-methoxy-6-oxo-6H-benzo[c]chromen-3-yl)cyclopentyl)hexanenitrile (28). To a stirred solution of **27** (105 mg, 0.21 mmol) in DMSO (4.1 mL), at room temperature under an argon atmosphere, was added NaCN (61 mg, 1.2 mmol). After stirring at the same temperature overnight, ice-cold water was added into the reaction mixture. The mixture was extracted with Et_2O and the combined organic layer was washed with brine, dried over $MgSO_4$, and the solvent was removed under reduced pressure. The residue was purified by flash column chromatography on silica gel (5-30% EtOAc/hexane) gave **28** (76 mg, 90%) as a white amorphous solid. IR (thin film, cm^{-1}) 3289 (OH), 2935, 2866, 2245 (CN), 1714 (CO), 1603, 1402, 1343, 1261, 1017, 748; ^{1}H NMR (400 MHz, $CDCl_3$) δ 8.64 (d, J = 2.5 Hz, 1H, Ar-H), 8.52 (brs, 1H, OH), 8.35 (d, J = 8.8 Hz, 1H, Ar-H), 7.05 (dd, J = 8.8, 2.5 Hz, 1H, Ar-H), 6.83 (d, J = 1.7 Hz, 1H, Ar-H), 6.78 (d, J = 1.7 Hz, 1H, Ar-H), 3.97 (s, 3H, -OCH_3), 2.25 (t, J = 7.0 Hz, 2H), 2.00 – 1.88 (m, 2H), 1.85 –1.50 (m, 10H), 1.39 – 1.29 (m, 2H), 1.14 – 0.98 (m, 2H). ^{13}C NMR (100 MHz, $CDCl_3$) δ 164.6, 161.7, 155.3, 152.7, 151.5, 137.2, 132.1, 119.8, 114.7, 113.6, 110.8, 110.7, 107.7, 104.8, 55.5, 51.1, 41.5, 37.6, 29.1, 25.1, 24.5, 23.2, 17.0. HRMS (ESI) for $C_{25}H_{28}NO_4$: calculated 406.2018; found 406.2010. Mass spectrum (ESI) m/z (relative intensity) 406 (M^+ + H, 100). LC/MS analysis (Waters MicroMass ZQ system) showed retention time 5.1 min for the title compound.

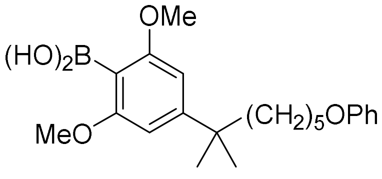

(2,6-Dimethoxy-4-(2-methyl-7-phenoxyheptan-2-yl)phenyl)boronic acid (30). The synthesis was carried out as described for **17**, using **29**[3] (1010 mg, 2.95 mmol), *n*-BuLi (2.4 mL of a 2.5 M solution in hexane, 5.9 mmol) and trimethyl borate (1.53 g, 14.75 mmol) in dry THF (7.3 mL). The crude oil obtained after acidic work up was purified by flash column chromatography on silica gel (10-30% EtOAc/hexane) to give **30** (695 mg, 61 % yield) as a colorless viscous oil. IR (thin film, cm^{-1}) 3519 (br, OH), 2937, 2862, 1604, 1558, 1464, 1419, 1324, 1230, 1110, 1044. ^{1}H NMR (500 MHz, $CDCl_3$) δ 7.28–7.25 (m, 2H, Ar-H), 7.19 (s, 2H, 2 × OH), 6.94–6.91 (m, 1H, Ar-H), 6.87–6.84 (m, 2H, Ar-H), 6.58 (s, 2H, Ar-H), 3.90 (s, 6H, 2 × OCH_3), 3.89 (t, J = 6.4 Hz, 2H, OCH_2), 1.75–1.70 (m, 2H, CH_2), 1.65–1.62 (m, 2H, CH_2), 1.43–1.37 (m, 2H, CH_2), 1.31 (s, 6H, 2 × CH_3), 1.17–1.11 (m, 2H, CH_2). ^{13}C NMR (126 MHz, $CDCl_3$) δ 165.3, 159.0, 155.9, 129.4, 120.5, 114.4, 103.4, 102.3, 67.6, 55.9, 44.2, 38.6, 29.1, 28.8, 26.7, 24.5. Mass spectrum (ESI) m/z (relative intensity) 409 (M^+ + Na, 50), 249 (M^+ + H – OPh

– $B(OH)_2$, 100). LC/MS analysis (Waters MicroMass ZQ system) showed retention time 5.3 min for the title compound.

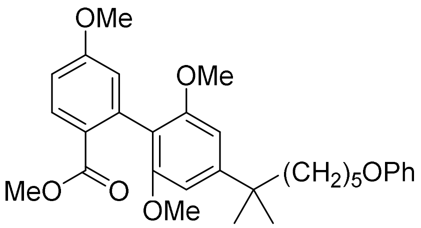

Methyl 2',5,6'-trimethoxy-4'-(2-methyl-7-phenoxyheptan-2-yl)-[1,1'-biphenyl]-2-carboxylate (31). The synthesis was carried out as described for **15**, using boronic acid **30** (600 mg, 1.55 mmol), **14** (382 mg, 1.56 mmol), Cs_2CO_3 (2.02 g, 6.2 mmol) and $Pd(PPh_3)_4$ (173 mg, 0.15 mmol) in DME/H_2O (5:1, 4.5 mL of DME and 0.9 mL of H_2O). The reaction was completed in 45 min and the crude oil obtained after work up was purified by flash column chromatography on silica gel (10-30% EtOAc/hexane) to give **31** (695 mg, 84 % yield) as a yellow oil. IR (thin film, cm^{-1}) 2937, 2862, 1727 (CO), 1600, 1575, 1463, 1406, 1241, 1123, 1034. ^1H NMR (500 MHz, $CDCl_3$) δ 7.96 (d, J = 8.7 Hz, 1H, Ar-H), 7.29–7.25 (m, 2H, Ar-H), 6.94–6.90 (m, 1H, Ar-H), 6.90–6.87 (m, 3H, Ar-H), 6.85 (d, J = 2.7 Hz, 1H, Ar-H), 6.59 (s, 2H, Ar-H), 3.93 (t, J = 6.5 Hz, 2H, OCH_2), 3.84 (s, 3H, OCH_3), 3.70 (s, 6H, 2 × OCH_3), 3.56 (s, 3H, OCH_3), 1.79–1.73 (m, 2H, CH_2), 1.69–1.66 (m, 2H, CH_2), 1.46–1.36 (m, 2H, CH_2), 1.36 (s, 6H, 2 × CH_3), 1.26–1.20 (m, 2H, CH_2). ^{13}C NMR (126 MHz, $CDCl_3$) δ 167.7, 161.8, 159.2, 156.7, 150.8, 137.7, 132.0, 129.5, 124.1, 120.6, 117.9, 116.8, 114.5, 112.3, 102.2, 67.8, 55.9, 55.3, 51.3, 44.8, 38.4, 29.4, 29.1, 26.9, 24.7. Mass spectrum (ESI) m/z (relative intensity) 529 (M^+ + Na, 50), 475 (M^+ – MeO, 100). LC/MS analysis (Waters MicroMass ZQ system) showed retention time 5.8 min for the title compound.

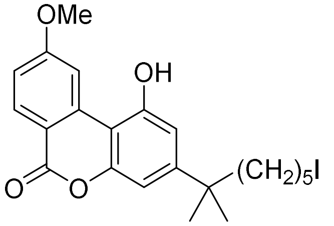

1-Hydroxy-3-(7-iodo-2-methylheptan-2-yl)-9-methoxy-6*H*-benzo[*c*]chromen-6-one (32). The synthesis was carried out as described for **1a**, using **31** (352 mg, 0.69 mmol), 9-iodo-9-BBN (3.13 mL of a 1.0 M in hexane, 3.13 mmol) in anhydrous CH_2Cl_2 (7.5 mL). The reaction was quenched by ethanolamine (1 mL) and the crude obtained after work up was purified by flash column chromatography on silica gel (CH_2Cl_2/Et_2O/hexane, 2:2:6) to give **32** (224 mg, 68 % yield) as a white solid. Mp 150−151 °C. IR (thin film, cm^{-1}) 3297 (br, OH), 2932, 2859, 1683 (CO), 1605, 1401, 1267, 1226, 1103, 1026. ^1H NMR (500 MHz, $CDCl_3$) δ 8.49 (d, J = 2.5 Hz, 1H, Ar-H), 8.36 (d, J = 8.9 Hz, 1H, Ar-H), 7.07 (dd, J = 8.9, 2.5 Hz, 1H, Ar-H), 6.94 (d, J = 1.8 Hz, 1H, Ar-H), 6.65 (d, J = 1.8 Hz, 1H, Ar-H), 5.65 (brs, 1H, OH), 3.97 (s, 3H, OCH_3), 3.12 (t, J = 7.0 Hz, 2H, CH_2I), 1.77–1.71 (m, 2H, CH_2), 1.62–1.59 (m, 2H, CH_2), 1.37–1.27 (m, 2H, CH_2), 1.30 (s, 6H, 2 × CH_3), 1.14–1.04 (m, 2H, CH_2). ^{13}C NMR (126 MHz, $CDCl_3$) δ 165.0, 163.0, 155.2, 152.8, 152.6, 137.5, 132.1, 115.3, 113.0, 110.7, 110.4, 107.1, 104.9, 55.7, 44.0, 37.9, 33.3, 31.1, 28.6, 23.7, 7.1. Mass spectrum (ESI) m/z (relative intensity) 481 (M^+ + H, 100). LC/MS analysis (Waters MicroMass ZQ system) showed retention time 5.4 min for the title compound.

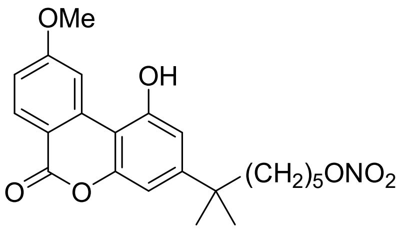

6-(1-Hydroxy-9-methoxy-6-oxo-6H-benzo[c]chromen-3-yl)-6-methylheptyl nitrate (33). To a solution of **32** (32 mg, 0.067 mmol) in CH_3CN (3.3 mL) at room temperature was added $AgNO_3$ (33 mg, 0.196 mmol). After being flushed with argon, the reaction mixture was stirred at room temperature until most of the starting material was consumed as judged by TLC (ca. 4.5 h). Celite was added, and CH_3CN was removed under reduced pressure. The residue was purified by silica gel column chromatography with EtOAc/hexane (1/4) as eluent to afford **33** (25 mg, 91%) as a white solid. Mp 131−132 ºC. IR (thin film, cm^{-1}) 3255 (br, OH), 2937, 2862, 1682 (CO), 1620, 1602, 1400, 1275, 1224, 1104, 1024, 864. ^1H NMR (500 MHz, $CDCl_3$) δ 8.50 (d, J = 2.5 Hz, 1H, Ar-H), 8.36 (d, J = 8.8 Hz, 1H, Ar-H), 7.07 (dd, J = 8.8, 2.5 Hz, 1H, Ar-H), 6.93 (d, J = 1.8 Hz, 1H, Ar-H), 6.67 (d, J = 1.8 Hz, 1H, Ar-H), 5.89 (s, 1H, OH), 4.37 (t, J = 6.6 Hz, 2H, CH_2ONO_2), 3.97 (s, 3H, OCH_3), 1.66–1.59 (m, 4H, 2 × CH_2), 1.36–1.23 (m, 2H, CH_2), 1.30 (s, 6H, 2 × CH_3), 1.15–1.05 (m, 2H, CH_2). ^{13}C NMR (126 MHz, $CDCl_3$) δ 165.1, 163.0, 155.3, 152.7, 152.6, 137.5, 132.1, 115.3, 113.0, 110.8, 110.4, 107.0, 104.9, 73.3, 55.7, 43.9, 37.9, 28.6, 26.6, 26.2, 24.3. HRMS (ESI) for $C_{22}H_{26}NO_7$: calculated 416.1719; found 416.1702. Mass spectrum (ESI) m/z (relative intensity) 416 (M^+ + H, 100), 370 (M^+ + H – NO_2, 10). LC/MS analysis (Waters MicroMass ZQ system) showed retention time 5.2 min for the title compound.

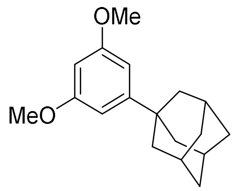

1-(3,5-Dimethoxyphenyl)adamantane (34).[4] To a solution of triflate **43** (2.1 g, 5.0 mmol) in DMF (25 mL) at room temperature was subsequently added $PdCl_2(PPh_3)_2$ (190 mg, 0.27 mmol), dppp (206 mg, 0.5 mmol), *n*-Bu_3N (6 mL, 25.0 mmol), HCOOH (88% w/v in H_2O, 0.5 mL, 11.7 mmol), and PMHS (350 mg). After being flushed with argon, the reaction mixture was stirred at room temperature for 5 min and then at 95 ºC for 19 h. The mixture was cooled to room temperature, diluted with Et_2O (ca. 13 mL) and aqueous 1 M HCl (ca. 5 mL), and stirred for 30 min. The organic material was extracted with Et_2O, and the combined organic extracts were washed with brine, dried over $MgSO_4$, filtered, and concentrated under reduced pressure. The residue was purified by silica gel column chromatography with (0-10% acetone/hexane) as eluent to afford **34** (1.25 g, 92%) as a white solid. Mp 54–55 ºC. Literature,[5] Mp 47–48 ºC. IR (thin film, cm^{-1}) 2900, 2847, 1594, 1453, 1421, 1320, 1203, 1151, 1068, 1037, 926, 844. ^1H NMR (500 MHz, $CDCl_3$) δ 6.58 (d, J = 2.3 Hz, 2H, Ar-H), 6.35 (dd, J = 2.3, 2.3 Hz, 1H, Ar-H), 3.83 (s, 6H, 2 × OCH_3), 2.13–2.12 (m, 3H, Ad-H), 1.94–1.93 (m, 6H, Ad-H), 1.84–1.76 (m, 6H, Ad-H). ^{13}C NMR (126 MHz, $CDCl_3$) δ 160.7, 154.2, 103.6, 97.1, 55.3, 43.2, 36.9, 36.6, 29.1. Mass spectrum (ESI) m/z (relative intensity) 273 (M^+ + H, 100). LC/MS analysis (Waters MicroMass ZQ system) showed retention time 5.5 min for the title compound.

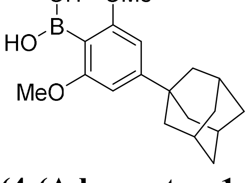

(4-(Adamantan-1-yl)-2,6-dimethoxyphenyl)boronic acid (35). The synthesis was carried out as described for **17**, using **34** (1.2 g, 4.41 mmol), *n*-BuLi (3.5 mL of a 2.5 M solution in hexane, 8.82 mmol) and trimethyl borate (2.29 g, 22.05 mmol) in dry THF (7.5 mL). The crude oil obtained after acidic work up was purified by flash column chromatography on silica gel (10-20% EtOAc/hexane) to give **35** (850 mg, 61 % yield) as a colorless oil.

IR (thin film, cm^{-1}) 3494 (OH), 2904, 2847, 1607, 1558, 1455, 1415, 1332, 1118, 1081. ^{1}H NMR (500 MHz, $CDCl_3$) δ 7.19 (s, 2H, 2 × OH), 6.62 (s, 2H, Ar-H), 3.92 (s, 6H, 2 × OCH_3), 2.11–2.10 (m, 3H, Ad-H), 1.91–1.90 (m, 6H, Ad-H), 1.82–1.74 (m, 6H, Ad-H). ^{13}C NMR (126 MHz, $CDCl_3$) δ 165.1, 157.1, 103.1, 101.0, 55.6, 42.6, 36.8, 36.4, 28.5. Mass spectrum (ESI) m/z (relative intensity) 317 (M^+ + H, 65), 273 (M^+ + 2H – $B(OH)_2$, 100). LC/MS analysis (Waters MicroMass ZQ system) showed retention time 5.1 min for the title compound.

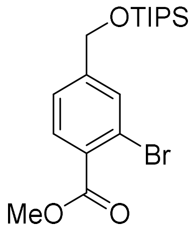

Methyl 2-bromo-4-(((triisopropylsilyl)oxy)methyl)benzoate (37). To a solution of **36** (4.40 g, 17.95 mmol) in DMF (18 mL) under an argon atmosphere at 0 °C was added imidazole (2.44 g, 35.90 mmol) and triisopropylsilyl chloride (4.1 mL, 18.85 mmol), and the reaction mixture was stirred at room temperature for 2 h. Water was added to the reaction mixture, and the organic material was extracted with EtOAc. The combined organic extracts were washed with brine, dried over Na_2SO_4, filtered, and concentrated under reduced pressure. The residue was purified by silica gel column chromatography with (0-10% EtOAc/hexane) as eluent to afford **37** (6.85 g, 95%) as a colorless oil. IR (thin film, cm^{-1}) 2944, 2866, 1735 (CO), 1605, 1463, 1434, 1290, 1246, 1151, 1039, 882. ^{1}H NMR (500 MHz, $CDCl_3$) δ 7.79 (d, J = 8.1 Hz, 1H, Ar-H), 7.66 (d, J = 1.5 Hz, 1H, Ar-H), 7.34 (dd, J = 8.1, 1.5 Hz, 1H, Ar-H), 4.83 (s, 2H, CH_2), 3.92 (s, 3H, $COOCH_3$), 1.21–1.14 (m, 3H, 3 × CH), 1.09 (d, J = 7.2 Hz, 18H, 6 × CH_3); ^{13}C NMR (126 MHz, $CDCl_3$) δ 166.3, 147.1, 131.4, 131.4, 130.0, 124.1, 121.8, 63.8, 52.2, 18.0, 12.0.

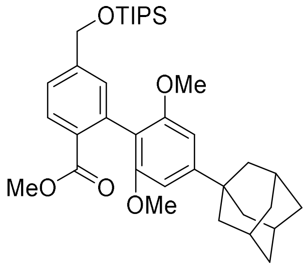

Methyl 4'-(adamantan-1-yl)-2',6'-dimethoxy-5-(((triisopropylsilyl)oxy)methyl)-[1,1'-biphenyl]-2-carboxylate (38). The synthesis was carried out as described for **15**, using boronic acid **35** (800 mg, 2.53 mmol), methyl 2-bromo-4-(((triisopropylsilyl)oxy)methyl)benzoate (**37**, 1015 mg, 2.53 mmol), Cs_2CO_3 (2.1 g, 6.44 mmol) and $Pd(PPh_3)_4$ (289 mg, 0.25 mmol) in DME/H_2O (5:1, 5 mL of DME and 1 mL of H_2O). The reaction was completed in 45 min and the crude oil obtained after work up was purified by flash column chromatography on silica gel (10-25% EtOAc/hexane) to give **38** (1.25 g, 83 % yield) as a white solid. Mp 173–174 °C. IR (thin film, cm^{-1}) 2903, 2865, 2849, 1733 (CO), 1609, 1574, 1461, 1449, 1407, 1283, 1238, 1125, 1103. ^{1}H NMR (500 MHz, $CDCl_3$) δ 7.90 (d, J = 8.1 Hz, 1H, Ar-H), 7.37 (dd, J = 8.1, 1.6 Hz, 1H, Ar-H), 7.30 (d, J = 1.6 Hz, 1H, Ar-H), 6.61 (s, 2H, Ar-H), 4.87 (s, 2H, CH_2), 3.69 (s, 6H, 2 × OCH_3), 3.60 (s, 3H, $COOCH_3$), 2.13–2.12 (m, 3H, Ad-H), 1.98–1.97 (m, 6H, Ad-H), 1.83–1.77 (m, 6H, Ad-H), 1.20–1.13 (m, 3H, 3 × CH), 1.08 (d, J = 7.1 Hz, 18H, 6 × CH_3). ^{13}C NMR (126 MHz, $CDCl_3$) δ 168.2, 156.7, 152.4, 144.7, 135.0, 130.3, 130.0, 129.6, 124.0, 116.5, 101.0, 64.8, 55.7, 51.4, 43.3, 36.9, 36.9, 29.1, 18.1, 12.0.

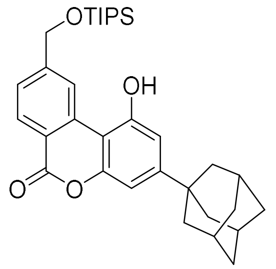

3-(Adamantan-1-yl)-1-hydroxy-9-(((triisopropylsilyl)oxy)methyl)-6*H*-benzo[*c*]chromen-6-one (39). The synthesis was carried out as described for **1a**, using **38** (1000 mg, 1.69 mmol), 9-iodo-9-BBN (5.5 mL of a 1.0 M in hexane, 5.5 mmol) in anhydrous CH_2Cl_2 (15 mL). The reaction was quenched by ethanolamine (1.3 mL) and the crude obtained after work up was purified by flash column chromatography on silica gel (CH_2Cl_2/Et_2O/hexane, 2:2:6) to give **39** (657 mg, 73 % yield) as a white solid. Mp 246−247 °C. IR (thin film, cm^{-1}) 3344 (br, OH), 2902, 2848, 1691 (CO), 1612, 1402, 1278, 1261, 1099. ^{1}H NMR (500 MHz, DMSO-d_6) δ 10.76 (brs, 1H, OH), 9.14 (d, J = 1.7 Hz, 1H, Ar-H), 8.18 (d, J = 8.1 Hz, 1H, Ar-H), 7.46 (dd, J = 8.1, 1.7 Hz, 1H, Ar-H), 6.91 (d, J = 1.8 Hz, 1H, Ar-H), 6.80 (d, J = 1.8 Hz, 1H, Ar-H), 4.94 (s, 2H, CH_2), 2.06–2.01 (m, 3H, Ad-H), 1.85–1.81 (m, 6H, Ad-H), 1.76–1.65 (m, 6H, Ad-H), 1.20–1.09 (m, 3H, 3 × CH), 1.04 (d, J = 7.3 Hz, 18H, 6 × CH_3). ^{13}C NMR (126 MHz, DMSO-d_6) δ 161.0, 156.8, 154.1, 152.7, 149.2, 135.2, 129.7, 125.1, 124.0, 118.9, 109.1, 104.7, 104.3, 64.8, 42.7, 36.5, 36.4, 28.7, 18.3, 11.9.

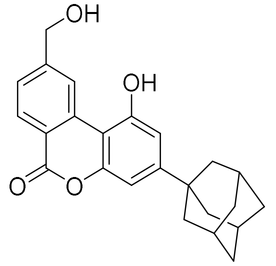

3-(Adamantan-1-yl)-1-hydroxy-9-(hydroxymethyl)-6*H*-benzo[*c*]chromen-6-one (40). To a solution of **39** (53 mg, 0.1 mmol) in THF (2.5 mL) under an argon atmosphere at room temperature was added a 1 M solution of tetrabutylammonium fluoride in THF (0.3 mL, 0.3 mmol), and the reaction mixture was stirred at room temperature for 2 h. Saturated aqueous NH_4Cl (1 mL) was added, and the organic material was extracted with EtOAc. The combined organic extracts were washed with brine, dried over $MgSO_4$, filtered, and concentrated under reduced pressure. The residue was purified by silica gel column chromatography with EtOAc/hexane (3/2) as eluent to afford **40** (34 mg, 90%) as a white solid. Mp 256–257 °C. IR (thin film, cm^{-1}) 3230 (br, OH), 2902, 2848, 1691 (CO), 1631, 1405, 1291, 1243, 1108. ^{1}H NMR (500 MHz, DMSO-d_6) δ 10.79 (brs, 1H, OH), 9.04 (d, J = 1.7 Hz, 1H, Ar-H), 8.19 (d, J = 8.1 Hz, 1H, Ar-H), 7.50 (dd, J = 8.1, 1.7 Hz, 1H, Ar-H), 6.89 (d, J = 1.8 Hz, 1H, Ar-H), 6.82 (d, J = 1.8 Hz, 1H, Ar-H), 5.49 (t, J = 5.3 Hz, 1H, OH), 4.66 (d, J = 5.3 Hz, 2H, CH_2), 2.07–2.02 (m, 3H, Ad-H), 1.87–1.82 (m, 6H, Ad-H), 1.76–1.67 (m, 6H, Ad-H). ^{13}C NMR (126 MHz, DMSO-d_6) δ 161.0, 156.7, 154.0, 152.7, 150.5, 135.0, 129.8, 126.0, 124.5, 118.7, 109.0, 104.9, 104.3, 63.3, 42.7, 36.5, 36.4, 28.7. HRMS (ESI) for $C_{24}H_{25}O_4$: calculated 377.1753; found 377.1748. Mass spectrum (ESI) m/z (relative intensity) 377 (M^+ + H, 100). LC/MS analysis (Waters MicroMass ZQ system) showed retention time 4.9 min for the title compound.

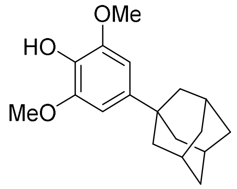

4-(Adamantan-1-yl)-2,6-dimethoxyphenol (42). To a homogeneous mixture of 1-adamantanol (6.91 g, 45.4 mmol) and 99% methanesulfonic acid (22.4 mL) at 40 °C was added 2,6-dimethoxyphenol (**41**, 7.0 g, 45.4 mmol). After being flushed with argon, the reaction mixture was stirred at 50 °C for 3 h. The mixture was cooled to room

temperature, ice cold water was added, and the organic material was extracted with CH_2Cl_2. The combined organic extracts were washed with saturated aqueous $NaHCO_3$, water, and brine, dried over $MgSO_4$, filtered, and concentrated under reduced pressure. The residue was purified by silica gel column chromatography with acetone/hexane (0/1 → 3/7) as eluent to afford **42** (7.59 g, 58%) as a white solid. Mp 115−116 ºC. Literature,[5] Mp 111–112 ºC. IR (thin film, cm^{-1}) 3465 (OH), 2897, 2847, 1607, 1522, 1451, 1354, 1265, 1189, 1114. ^1H NMR (500 MHz, $CDCl_3$) δ 6.59 (s, 2H, Ar-H), 5.41 (brs, 1H, OH), 3.90 (s, 6H, 2 × OCH_3), 2.10–2.09 (m, 3H, Ad-H), 1.90–1.89 (m, 6H, Ad-H), 1.81–1.74 (m, 6H, Ad-H). ^{13}C NMR (126 MHz, $CDCl_3$) δ 146.5, 142.6, 132.6, 101.8, 56.1, 43.3, 36.6, 36.0, 28.9. Mass spectrum (ESI) m/z (relative intensity) 289 (M^+ + H, 50), 135 (M^+ + H – $C_6H_2(OMe)_2(OH)$, 100). LC/MS analysis (Waters MicroMass ZQ system) showed retention time 4.9 min for the title compound.

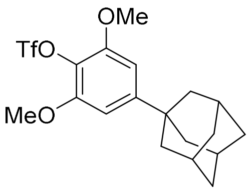

4-(Adamantan-1-yl)-2,6-dimethoxyphenyl trifluoromethanesulfonate (43). To a solution of **42** (4.30 g, 14.9 mmol) in CH_2Cl_2 (83 mL) under an argon atmosphere at room temperature was subsequently added *N*-phenyltrifluoromethanesulfonimide (6.14 g, 17.2 mmol), Et_3N (2.4 mL, 17.2 mmol), and DMAP (421 mg, 3.45 mmol) and the reaction mixture was refluxed for 18 h. After the mixture was cooled to room temperature, a 3 M aqueous solution of NaOH was added, and the organic material was extracted with CH_2Cl_2. The combined organic extracts were washed with brine, dried over $MgSO_4$, filtered, and concentrated under reduced pressure. The residue was purified by silica gel column chromatography with EtOAc/hexane (1/9) as eluent to afford **43** (5.94 g, 95%) as a white solid. Mp 151–152 ºC. IR (thin film, cm^{-1}) 2905, 2849, 1602, 1410, 1250, 1226, 1201, 1133, 889. ^1H NMR (500 MHz, $CDCl_3$) δ 6.62 (s, 2H, Ar-H), 3.89 (s, 6H, 2 × OCH_3), 2.12–2.11 (m, 3H, Ad-H), 1.91–1.90 (m, 6H, Ad-H), 1.83–1.74 (m, 6H, Ad-H). ^{13}C NMR (126 MHz, $CDCl_3$) δ 152.7, 151.9, 126.0, 118.8 (q, $^1J_{CF}$ = 320.6 Hz), 101.9, 56.2, 43.2, 37.0, 36.7, 29.0; ^{19}F NMR (470 MHz, $CDCl_3$) δ –73.8. Mass spectrum (ESI) m/z (relative intensity) 421 (M^+ + H, 20), 273 (M^+ + 2H –CF_3SO_3, 100). LC/MS analysis (Waters MicroMass ZQ system) showed retention time 5.9 min for the title compound.

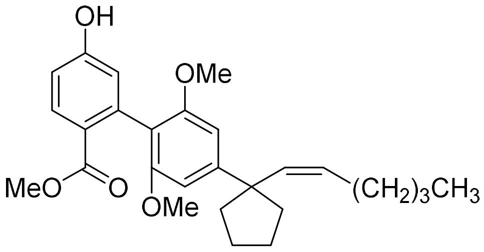

Methyl (Z)-4'-(1-(hex-1-en-1-yl)cyclopentyl)-5-hydroxy-2',6'-dimethoxy-[1,1'-biphenyl]-2-carboxylate (45). The synthesis was carried out as described for **15**, using boronic acid **21** (153 mg, 0.46 mmol), **44** (91 mg, 0.39 mmol), Cs_2CO_3 (520 mg, 1.6 mmol) and $Pd(PPh_3)_4$ (46 mg, 0.04 mmol) in DME/H_2O (5:1, 1.0 mL of DME and 0.2 mL of H_2O). The reaction was completed in 45 min and the crude oil obtained after work up was purified by flash column chromatography on silica gel (10-80% Et_2O/hexane) to give **45** (140 mg, 82 % yield) as a yellow oil. IR (thin film, cm^{-1}) 3087, 2978, 2911, 2854, 1723 (CO), 1575, 1435, 1122, 1021, 773; ^1H NMR (500 MHz, $CDCl_3$) δ 7.89 (d, *J* = 8.5 Hz, 1H, Ar-H), 6.80 (dd, *J* = 8.5, 2.6 Hz, 1H, Ar-H), 6.73 (d, *J* = 2.6 Hz, 1H, Ar-H), 6.62 (s, 2H, Ar-H), 5.74 (d, *J* = 11.3 Hz, 1H), 5.37 (brs, 1H, OH), 5.34 (dt, *J* = 11.3, 7.4 Hz, 1H), 3.67 (s, 6H, -OCH_3), 3.54 (s, 3H, -$COOCH_3$), 2.12 – 2.03 (m, 2H), 2.03 – 1.95 (m, 2H), 1.87 – 1.71 (m, 6H), 1.19 – 1.45 (m, 4H), 0.80 (t, *J* = 7.0 Hz, 3H). Mass spectrum (ESI) m/z (relative intensity) 439 (M^+ + H, 100).

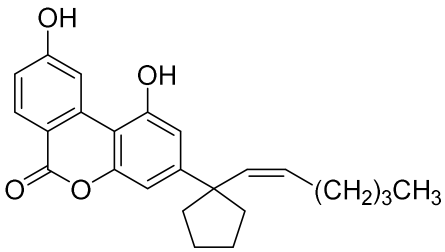

(Z)-3-(1-(Hex-1-en-1-yl)cyclopentyl)-1,9-dihydroxy-6H-benzo[c]chromen-6-one (46). The synthesis was carried out as described for **1a**, using **45** (118 mg, 0.27 mmol), 9-iodo-9-BBN (0.82 mL of a 1.0 M in hexane, 0.82 mmol) in anhydrous CH_2Cl_2 (3.4 mL). The reaction was quenched by ethanolamine (0.5 mL) and the crude obtained after work up was purified by flash column chromatography on silica gel (30-80% Et_2O in hexane) to give **46** (66 mg, 65 % yield) as a white solid. Mp 134-136 ºC. IR (thin film, cm^{-1}) 3269 (OH), 2955, 2873, 1679 (CO), 1604, 1397, 1102, 1027, 750; ^{1}H NMR (500 MHz, $CDCl_3$) δ 8.45 (d, J = 1.7 Hz, 1H, Ar-H), 8.29 (d, J = 8.3 Hz, 1H, Ar-H), 6.96 (dd, J = 8.3, 1.7 Hz, 1H, Ar-H), 6.94 (d, J = 1.4 Hz, 1H, Ar-H), 6.70 (d, J = 1.4 Hz, 1H, Ar-H), 5.70 (d, J = 11.3 Hz, 1H), 5.32 (dt, J = 11.3, 7.4 Hz, 1H), 2.05-1.90 (m, 4H), 1.81 – 1.66 (m, 6H), 1.14-1.06 (m, 4H), 0.69 (t, J = 6.8 Hz, 3H). Mass spectrum (ESI) m/z (relative intensity) 379 (M^+ + H, 100). LC/MS analysis (Waters MicroMass ZQ system) showed retention time 5.2 min for the title compound.

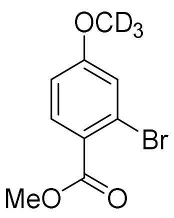

Methyl 2-bromo-4-(methoxy-*d₃*)benzoate (47). To a solution of methyl 2-bromo-4-hydroxybenzoate (**44**, 1.0 g, 4.35 mmol) in anhydrous DMF (24 mL) under an argon atmosphere was added K_2CO_3 (1.44 g, 10.44 mmol), and the mixture was stirred at room temperature for 20 minutes. The mixture was cooled at 0 ºC and a solution of CD_3I (756 mg, 5.22 mmol) in 1.3 mL of dry DMF was added dropwise. After 5 minutes stirring at 0 ºC, the mixture was stirred at room temperature for 2.5 h. The reaction was quenched with H_2O at 0 ºC and then Et_2O was added. The water phase was extracted with Et_2O and the combined organic phase were washed with brine and dried over $MgSO_4$. The solvent was removed, and the crude product was purified by flash column chromatography on silica gel (10% EtOAc/hexane) to give **47** (930 mg, 86 % yield) as colorless oil. IR (thin film, cm^{-1}) 1725 (CO), 1595, 1489, 1257, 1241, 1099, 999, 768. ^{1}H NMR ($CDCl_3$, 500 MHz) δ 7.84 (d, J = 8.0 Hz, 1H, Ar-H), 7.17 (d, J = 2.0 Hz, 1H, Ar-H), 6.84 (dd, J = 8.0, 2.0 Hz, 1H, Ar-H), 3.88 (s, 3H, $-OCH_3$). ^{13}C NMR ($CDCl_3$, 100 MHz) δ 165.9, 162.3, 133.2, 123.6, 123.4, 119.8, 113.0, 52.1.

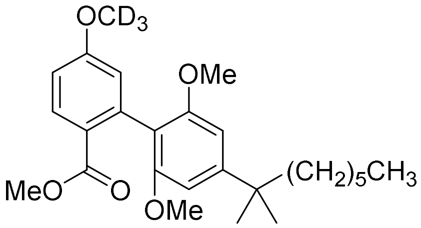

Methyl 2',6'-dimethoxy-5-(methoxy-*d₃*)-4'-(2-methyloctan-2-yl)-[1,1'-biphenyl]-2-carboxylate (48). The synthesis was carried out as described for **15**, using boronic acid **13** (1.04 g, 3.38 mmol), **47** (699 mg, 2.82 mmol), Cs_2CO_3 (3.67 g, 11.27 mmol) and $Pd(PPh_3)_4$ (325 mg, 0.28 mmol) in DME/H_2O (5:1, 8 mL of DME and 1.6 mL of H_2O). The reaction was completed in 45 min and the crude oil obtained after work up was purified by flash

column chromatography on silica gel (10-30% EtOAc/hexane) to give **48** (984 mg, 81 % yield) as a yellow oil. IR (thin film, cm^{-1}) 2930, 2073 (CD), 1727 (CO), 1601, 1576, 1406, 1224, 1126, 1104, 745. ^{1}H-NMR ($CDCl_3$, 500 MHz) δ 7.93 (d, *J* = 8.0 Hz, 1H, Ar-H), 6.86 (dd, *J* = 8.0, 2.1 Hz, 1H, Ar-H), 6.81 (d, *J* = 2.1 Hz, 1H, Ar-H), 6.56 (s, 2H, Ar-H), 3.68 (s, 6H, $-OCH_3$), 3.53 (s, 3H, $-COOCH_3$), 1.62-1.59 (m, 2H, $-CH_2-$), 1.32 (s, 6H, $-CH_3$), 1.26-1.22 (m, 6H, $-CH_2-$), 1.11 (br s, 2H, $-CH_2-$), 0.84 (t, *J* = 7.0 Hz, 3H, $-CH_3$). ^{13}C-NMR ($CDCl_3$, 100 MHz) δ 167.9, 161.7, 156.6, 151.0, 137.5, 131.9, 124.1, 117.7, 116.7, 112.4, 102.2, 55.9, 51.2, 44.7, 38.3, 31.8, 30.0, 29.0, 24.7, 22.6, 14.0.

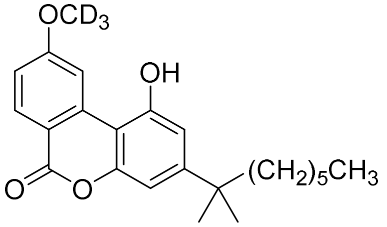

1-Hydroxy-9-(methoxy-*d*$_3$)-3-(2-methyloctan-2-yl)-6H-benzo[c]chromen-6-one (49). The synthesis was carried out as described for **1a**, using **48** (24 mg, 0.056 mmol), 9-iodo-9-BBN (0.23 mL of a 1.0 M in hexane, 0.23 mmol) in anhydrous CH_2Cl_2 (1 mL). The reaction was quenched by ethanolamine (0.3 mL) and the crude obtained after work up was purified by flash column chromatography on silica gel (CH_2Cl_2/Et_2O/hexane, 2:2:6) to give **49** (16 mg, 75 % yield) as a white solid. Mp 150−152 ºC. IR (thin film, cm^{-1}) 3421 (OH), 2931, 2073 (CD), 1676 (CO), 1626, 1607, 1402, 1303, 1111, 722. ^{1}H-NMR ($CDCl_3$, 500 MHz) δ 8.48 (d, *J* = 2.0 Hz, 1H, Ar-H), 8.34 (d, *J* = 8.0 Hz, 1H, Ar-H), 7.04 (dd, *J* = 8.0, 2.0 Hz, 1H, Ar-H), 6.93 (s, 1H, Ar-H), 6.66 (s, 1H, Ar-H), 5.75 (brs, 1H, -OH), 1.58-1.55 (m, 2H, $-CH_2-$), 1.27 (s, 6H, $-CH_3$), 1.23-1.17 (m, 6H, $-CH_2-$), 1.06-1.00 (m, 2H, $-CH_2-$), 0.81 (t, *J* = 7.1 Hz, 3H, $-CH_3$). ^{13}C-NMR ($CDCl_3$, 100 MHz) δ 164.8, 161.7, 153.9, 153.1, 152.9, 136.8, 132.2, 114.9, 113.6, 110.8, 109.8, 108.0, 104.8, 44.3, 37.9, 31.7, 29.9, 28.6, 24.6, 22.6, 14.0. HRMS (ESI) for $C_{23}H_{26}D_3O_4$: calculated 372.2254; found 372.2249. Mass spectrum (ESI) m/z (relative intensity) 372 (M^+ + H, 100). LC/MS analysis (Waters MicroMass ZQ system) showed retention time 5.5 min for the title compound.

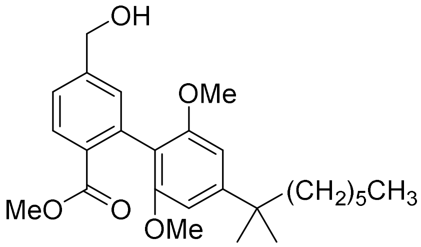

Methyl 5-(hydroxymethyl)-2',6'-dimethoxy-4'-(2-methyloctan-2-yl)-[1,1'-biphenyl]-2-carboxylate (50). The synthesis was carried out as described for **15**, using boronic acid **13** (754 mg, 2.45 mmol), **36** (580 mg, 2.36 mmol), Cs_2CO_3 (2.66 g, 8.16 mmol) and $Pd(PPh_3)_4$ (462 mg, 0.4 mmol) in DME/H_2O (5:1, 5 mL of DME and 1 mL of H_2O). The reaction was completed in 45 min and the crude oil obtained after work up was purified by flash column chromatography on silica gel (10-40% EtOAc/hexane) to give **50** (810 mg, 80 % yield) as a yellow oil. IR (thin film, cm^{-1}) 3235 (OH), 2916, 2895, 1698 (CO), 1613, 1403, 1105, 1045, 757; ^{1}H NMR (500 MHz, $CDCl_3$) δ 7.93 (d, *J* = 8.0 Hz, 1H, Ar-H), 7.37 (d, *J* = 8.0 Hz, 1H, Ar-H), 7.33 (s, 1H, Ar-H), 6.57 (s, 2H, Ar-OH), 4.76 (d, *J* = 4.3 Hz, 2H), 3.69 (s, 6H, $-OCH_3$), 3.58 (s, 3H, $-COOCH_3$), 1.65 – 1.58 (m, 2H), 1.33 (s, 6H, $-CH_3$), 1.29-1.18 (m, 6H), 1.15 – 1.08 (m, 2H), 0.86 (t, *J* = 6.9 Hz, 3H). ^{13}C NMR (100 MHz, $CDCl_3$) δ 168.2, 156.5, 151.1, 143.9, 135.4, 131.1, 130.7, 130.0, 125.0, 116.1, 102.1, 64.9, 55.8, 51.4, 44.7, 38.3, 31.8, 30.0, 28.9, 24.7, 22.6, 14.0.

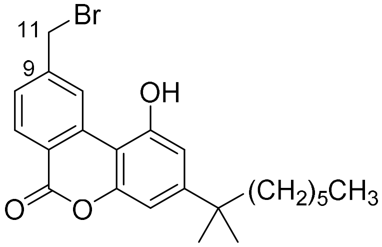

9-(Bromomethyl)-1-hydroxy-3-(2-methyloctan-2-yl)-6H-benzo[c]chromen-6-one (51). To a stirring solution of **50** (806 mg, 1.88 mmol) in dry CH_2Cl_2 at −78 °C was added dropwise BBr_3 (7.8 mL of a 1.0 M solution in hexane, 7.8 mmol). The temperature was slowly warmed to room temperature and the reaction mixture was stirred overnight. Then the reaction was cooled back to 0 °C and water was added. The organic phase was removed under reduced pressure and the residue water phase was extracted by EtOAc. The combined organic extracts were washed with brine, dried over $MgSO_4$ and concentrated under reduced pressure. Purification by flash column chromatography on silica gel (10-40% EtOAc/hexane) gave **51** (550 mg, 68 % yield) as a white solid. Mp 197-201 ºC (decomposed). IR (thin film, cm^{-1}) 3325 (OH), 2927, 2856, 1699 (CO), 1611, 1402, 1262, 1092, 749; ^1H NMR (500 MHz, $CDCl_3$) δ 8.99 (s, 1H, Ar-H), 8.38 (d, *J* = 8.1 Hz, 1H, Ar-H), 7.54 (d, *J* = 8.1 Hz, 1H, Ar-H), 6.95 (d, *J* = 1.5 Hz, 1H, Ar-H), 6.67 (d, *J* = 1.5 Hz, 1H, Ar-H), 5.66 (s, 1H, OH), 4.60 (s, 2H, CH_2Br), 1.61 – 1.55 (m, 2H), 1.28 (s, 6H, $-CH_3$), 1.26 – 1.14 (m, 6H), 1.08 – 0.99 (m, 2H), 0.82 (t, *J* = 6.9 Hz, 3H). ^{13}C NMR (100 MHz, $CDCl_3$) δ 161.5, 154.0, 153.5, 152.6, 144.6, 135.2, 130.7, 128.2, 127.6, 120.1, 110.1, 107.9, 104.3, 44.3, 38.0, 32.7, 31.7, 29.9, 28.6, 24.6, 22.6, 14.0. Mass spectrum (ESI) m/z (relative intensity) 431 (M^+ + H, 80), 433 (M^++H +2, 100). LC/MS analysis (Waters MicroMass ZQ system) showed retention time 5.7 min for the title compound.

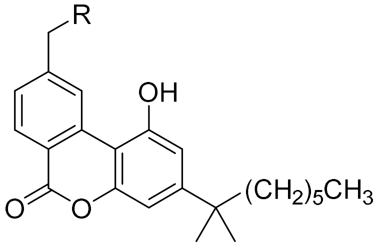

52a R = OMe
52b R = OEt
52c R = O*i*-Pr
52d R = N_3

1-Hydroxy-9-(methoxymethyl)-3-(2-methyloctan-2-yl)-6H-benzo[c]chromen-6-one (52a). Argon was bubbled through a mixture of **51** (17 mg, 0.039 mmol) and $FeSO_4 \cdot 7H_2O$ (44 mg, 0.16 mmol) in CH_2Cl_2/MeOH (4:1, 0.8 mL of CH_2Cl_2 and 0.2 mL of MeOH) for 10 min. Then the reaction mixture was microwaved at 130 °C for 60 min in a Biotage apparatus. Then the mixture was cooled to room temperature and filtered through a short celite pad. The filtrate was concentrated and Et_2O was added. The ether solution was washed with water and brine, dried over $MgSO_4$ and concentrated under reduced pressure. Purification by flash column chromatography on silica gel (10-30% EtOAc/hexane) gave **52a** (15 mg, 99 % yield) as a yellow oil. IR (thin film, cm^{-1}) 3306 (OH), 2939, 2850, 1730 (CO), 1586, 1435, 1176, 1021, 785, 724; ^1H NMR (500 MHz, $CDCl_3$) δ 9.05 (s, 1H, Ar-H), 8.41 (d, *J* = 8.1 Hz, 1H, Ar-H), 7.50 (d, *J* = 8.1 Hz, 1H, Ar-H), 6.93 (d, *J* = 1.6 Hz, 1H, Ar-H), 6.91 (brs, 1H, OH), 6.90 (s, 1H, Ar-H), 6.74 (d, *J* = 1.6 Hz, 1H, Ar-H), 4.70 (s, 2H), 3.50 (s, 3H, $-OCH_3$), 1.61 – 1.54 (m, 2H), 1.28 (s, 6H, $-CH_3$), 1.26 – 1.12 (m, 6H), 1.07 – 0.98 (m, 2H), 0.82 (t, *J* = 7.0 Hz, 3H). ^{13}C NMR (100 MHz, $CDCl_3$) δ 161.8, 154.3, 153.1, 152.6, 145.1, 135.0, 130.3, 126.5, 125.8, 119.8, 109.9, 107.7, 104.6, 74.5, 58.4, 44.3, 37.9, 31.7, 29.9, 28.6, 24.6, 22.6, 14.0. HRMS (ESI) for $C_{24}H_{31}O_4$: calculated 383.2222; found 383.2213. Mass spectrum (ESI) m/z (relative intensity) 383 (M^+ + H, 100). LC/MS analysis (Waters MicroMass ZQ system) showed retention time 5.7 min for the title compound.

9-(Ethoxymethyl)-1-hydroxy-3-(2-methyloctan-2-yl)-6H-benzo[c]chromen-6-one (52b). Argon was bubbled through a mixture of **51** (20 mg, 0.046 mmol) and $FeSO_4 \cdot 7H_2O$ (52 mg, 0.19 mmol) in CH_2Cl_2/EtOH (3:1, 0.9 mL of CH_2Cl_2 and 0.3 mL of EtOH) for 10 min. Then the reaction mixture was microwaved at 130 °C for 60 min in a Biotage apparatus. Then the mixture was cooled to room temperature and filtered through a short celite pad. The filtrate was concentrated and Et_2O was added. The ether solution was washed with water and brine, dried over $MgSO_4$ and concentrated under reduced pressure. Purification by flash column chromatography on silica gel (10-30% EtOAc/hexane) gave **52b** (18 mg, 98 % yield) as a yellow oil. IR (thin film, cm^{-1}) 3300 (OH), 2953, 2934, 2865,1711 (CO), 1699, 1591, 1466, 1378, 1054, 910, 740, 688; ^1H NMR (500 MHz, $CDCl_3$) δ 9.01 (s, 1H, Ar-H), 8.39 (d, J = 8.1 Hz, 1H, Ar-H), 7.51 (d, J = 8.1 Hz, 1H, Ar-H), 6.94 (d, J = 1.6 Hz, 1H, Ar-H), 6.69 (d, J = 1.6 Hz, 1H, Ar-H), 6.26 (s, 1H, OH), 4.72 (s, 2H), 3.65 (q, J = 7.0 Hz, 2H), 1.63 – 1.55 (m, 2H), 1.31 (t, J = 7.0 Hz, 3H), 1.29 (s, 6H, -CH_3), 1.27 – 1.14 (m, 6H), 1.09 – 1.08 (m, 2H), 0.83 (t, J = 7.0 Hz, 3H). ^{13}C NMR (100 MHz, $CDCl_3$) δ 161.6, 154.0, 152.9, 152.6, 145.7, 134.8, 130.3, 126.5, 125.6, 119.7, 109.8, 107.9, 104.7, 72.4, 66.2, 44.3, 37.9, 31.7, 29.9, 28.6, 24.6, 22.6, 15.2, 14.0. HRMS (ESI) for $C_{25}H_{33}O_4$: calculated 397.2379; found 397.2374. Mass spectrum (ESI) m/z (relative intensity) 397 (M^+ + H, 100). LC/MS analysis (Waters MicroMass ZQ system) showed retention time 5.7 min for the title compound.

1-Hydroxy-9-(isopropoxymethyl)-3-(2-methyloctan-2-yl)-6H-benzo[c]chromen-6-one (52c). Argon was bubbled through a mixture of **51** (20 mg, 0.046 mmol) and $FeSO_4 \cdot 7H_2O$ (52 mg, 0.19 mmol) in CH_2Cl_2/ propan-2-ol (3:1, 0.9 mL of CH_2Cl_2 and 0.3 mL of propan-2-ol) for 10 min. Then the reaction mixture was microwaved at 130 °C for 60 min in a Biotage apparatus. Then the mixture was cooled to room temperature and filtered through a short celite pad. The filtrate was concentrated and Et_2O was added. The ethereal solution was washed with water and brine, dried over $MgSO_4$ and concentrated under reduced pressure. Purification by flash column chromatography on silica gel (10-30% EtOAc/hexane) gave **52c** (18 mg, 97 % yield) as a yellow oil. IR (thin film, cm^{-1}) 3308 (OH), 2962, 2927, 2857, 1694 (CO), 1613, 1405, 1087, 845, 743; ^1H NMR (500 MHz, $CDCl_3$) δ 9.19 (s, 1H, Ar-H), 8.37 (d, J = 8.1 Hz, 1H, Ar-H), 7.74 (brs, 1H, OH), 7.45 (d, J = 8.1 Hz, 1H, Ar-H), 6.90 (d, J = 1.4 Hz, 1H, Ar-H), 6.78 (s, 1H, Ar-H), 4.81 (s, 2H), 3.82 (septet, J = 6.1 Hz, 1H), 1.62 – 1.54 (m, 2H), 1.33 (d, J = 6.1 Hz, 6H, -$CH(CH_3)_2$), 1.28 (s, 6H, -CH_3), 1.26 – 1.13 (m, 6H), 1.09 – 0.99 (m, 2H), 0.82 (t, J = 7.0 Hz, 3H). ^{13}C NMR (100 MHz, $CDCl_3$) δ 161.9, 154.8, 152.9, 152.6, 145.8, 135.2, 130.2, 126.2, 125.6, 119.6, 109.8, 107.3, 104.6, 71.8, 69.7, 44.3, 37.9, 31.7, 29.9, 28.6, 24.6, 22.6, 22.1, 14.0. HRMS (ESI) for $C_{26}H_{35}O_4$: calculated 411.2535; found 411.2528. Mass spectrum (ESI) m/z (relative intensity) 411 (M^+ + H, 100). LC/MS analysis (Waters MicroMass ZQ system) showed retention time 5.8 min for the title compound.

9-(Azidomethyl)-1-hydroxy-3-(2-methyloctan-2-yl)-6H-benzo[c]chromen-6-one (52d). To a stirring solution of **51** (30 mg, 0.07 mmol) in dry CH_2Cl_2 (1.4 mL) under argon atmosphere was added n-$Bu_4N^+N_3^-$ (79 mg, 0.28 mmol) at room temperature and the reaction mixture was stirred for 2 days. The mixture was then quenched by the addition of water, extracted with Et_2O and the combined organic extracts were washed with brine, dried over $MgSO_4$ and concentrated under reduced pressure. Purification by flash column chromatography on silica gel (15-30 % EtOAc/hexane) gave **52d** (26 mg, 96 % yield) as a white solid. Mp 137-141 °C. IR (thin film, cm^{-1}) 3285 (OH), 2926, 2135 (N_3), 1698 (CO), 1621, 1404, 1103, 1054, 846, 745; ^1H NMR (500 MHz, $CDCl_3$) δ 8.94 (s, 1H, Ar-H), 8.42 (d, J = 8.1 Hz, 1H, Ar-H), 7.47 (d, J = 8.1 Hz, 1H, Ar-H), 6.96 (d, J = 1.6 Hz, 1H, Ar-H), 6.69 (d, J = 1.6 Hz, 1H, Ar-H), 5.64 (s, 1H, OH), 4.53 (s, 2H, CH_2N_3), 1.62 – 1.55 (m, 2H), 1.29 (s, 6H, -CH_3), 1.27 – 1.12 (m, 6H), 1.07 – 1.00 (m, 2H), 0.82 (t, J = 6.9 Hz, 3H). ^{13}C NMR (100 MHz, $CDCl_3$) δ 161.2, 153.7, 153.5, 152.7, 142.3, 135.1, 130.7, 127.0, 126.4, 120.4, 110.0, 108.2, 104.4, 54.8, 44.3, 38.0, 31.7, 29.9, 28.6, 24.6, 22.5, 13.9. HRMS (ESI) for $C_{23}H_{28}N_3O_3$: calculated 394.2131; found 394.2124. Mass spectrum (ESI) m/z (relative intensity) 394 (M^+ + H, 100). LC/MS analysis (Waters MicroMass ZQ system) showed retention time 5.7 min for the title compound.

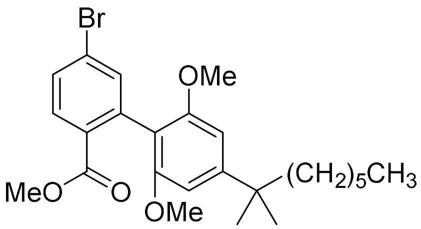

Methyl 5-bromo-2',6'-dimethoxy-4'-(2-methyloctan-2-yl)-[1,1'-biphenyl]-2-carboxylate (54). The synthesis was carried out as described for **15**, using boronic acid **13** (262 mg, 0.85 mmol), **53** (290 mg, 0.85 mmol), Cs_2CO_3 (1.1 g, 3.4 mmol) and $Pd(PPh_3)_4$ (104 mg, 0.09 mmol) in DME/H_2O (5:1, 3.5 mL of DME and 0.7 mL of H_2O). The reaction was completed in 45 min and the crude oil obtained after work up was purified by flash column chromatography on silica gel (10-30% EtOAc/hexane) to give **54** (250 mg, 62 % yield) as a yellow oil. IR (thin film, cm^{-1}) 2954, 2930, 2858, 1734 (CO), 1609, 1575, 1453, 1409, 1284, 1238, 1124, 1096, 1017, 829. ^{1}H NMR (500 MHz, $CDCl_3$) δ 7.78 (d, J = 8.3 Hz, 1H, Ar-H), 7.53–7.46 (m, 2H, Ar-H), 6.56 (s, 2H, Ar-H), 3.70 (s, 6H, 2 × OCH_3), 3.58 (s, 3H, $COOCH_3$), 1.66–1.58 (m, 2H, CH_2), 1.33 (s, 6H, 2 × CH_3), 1.29–1.20 (m, 6H, 3 × CH_2), 1.16–1.06 (m, 2H, CH_2), 0.86 (t, J = 6.8 Hz, 3H, CH_3). ^{13}C NMR (126 MHz, $CDCl_3$) δ 167.7, 156.4, 151.6, 137.1, 135.6, 131.1, 130.9, 129.8, 125.6, 114.9, 102.0, 55.7, 51.6, 44.7, 38.4, 31.9, 30.0, 29.0, 24.7, 22.7, 14.2. Mass spectrum (ESI) m/z (relative intensity) 477 (M^+ + H, 75), 445 (M^+ – OMe, 100), 155 (100). LC/MS analysis (Waters MicroMass ZQ system) showed retention time 6.2 min for the title compound.

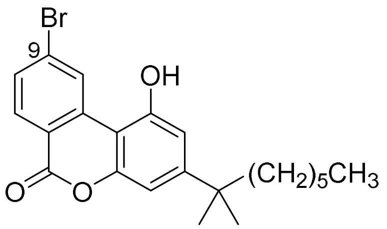

9-Bromo-1-hydroxy-3-(2-methyloctan-2-yl)-6*H*-benzo[*c*]chromen-6-one (55). The synthesis was carried out as described for **1a**, using **54** (205 mg, 0.43 mmol), 9-iodo-9-BBN (1.4 mL of a 1.0 M in hexane, 1.4 mmol) in anhydrous CH_2Cl_2 (5 mL). The reaction was quenched by ethanolamine (0.5 mL) and the crude obtained after work up was purified by flash column chromatography on silica gel (CH_2Cl_2/Et_2O/hexane, 2:2:6) to give **55** (130 mg, 73 % yield) as a white solid. Mp 232–233 ºC. IR (thin film, cm^{-1}) 3291 (br, OH), 2957, 2928, 2855, 1693 (CO), 1624, 1594, 1392, 1276, 1261, 1109, 1087. ^{1}H NMR (500 MHz, DMSO-d_6) δ 11.03 (s, 1H, OH), 9.18 (d, J = 2.0 Hz, 1H, Ar-H), 8.10 (d, J = 8.4 Hz, 1H, Ar-H), 7.73 (dd, J = 8.4, 2.0 Hz, 1H, Ar-H), 6.87 (d, J = 1.8 Hz, 1H, Ar-H), 6.80 (d, J = 1.8 Hz, 1H, Ar-H), 1.57–1.50 (m, 2H, CH_2), 1.23 (s, 6H, 2 × CH_3), 1.19–1.11 (m, 6H, 3 × CH_2), 1.03–0.94 (m, 2H, CH_2), 0.77 (t, J = 6.8 Hz, 3H, CH_3). ^{13}C NMR (126 MHz, DMSO-d_6) δ 160.5, 156.5, 153.7, 152.7, 136.6, 131.9, 130.9, 129.8, 129.4, 119.4, 110.1, 105.9, 103.0, 44.0, 38.1, 31.6, 29.8, 28.8, 24.6, 22.5, 14.3. Mass spectrum (ESI) m/z (relative intensity) 417 (M^+ + H, 100), 131 (85). LC/MS analysis (Waters MicroMass ZQ system) showed retention time 5.8 min for the title compound.

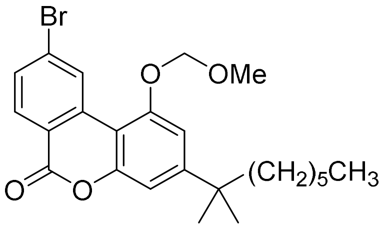

9-Bromo-1-(methoxymethoxy)-3-(2-methyloctan-2-yl)-6*H*-benzo[*c*]chromen-6-one (56). To a solution of **55** (125 mg, 0.3 mmol) in CH_2Cl_2 (6.0 mL) under an argon atmosphere at 0 ºC was added *N*,*N*-diisopropylethylamine (520 µL, 3.0 mmol), and the mixture was stirred at 0 ºC for 5 min. Chloromethyl methyl ether (120 µL, 1.6 mmol) was added at 0 ºC, and the reaction mixture was stirred at 0 ºC for an additional 30 min and then at room temperature for 1 h. The reaction mixture was quenched with saturated aqueous $NaHCO_3$, and the organic material was extracted with CH_2Cl_2. The combined organic extracts were washed with brine, dried over $MgSO_4$, filtered, and concentrated under reduced pressure. The residue was purified by silica gel column chromatography with EtOAc/hexane (0/1 → 1/9) as eluent to afford **56** (127 mg, 92%) as a white solid. Mp 68−69 ºC. IR (thin film, cm^{-1}) 2956, 2928, 2857, 1732 (CO), 1616, 1592, 1385, 1157, 1074, 1043, 1010, 965, 926. ^1H NMR (500 MHz, $CDCl_3$) δ 9.16 (d, *J* = 1.9 Hz, 1H, Ar-H), 8.26 (d, *J* = 8.5 Hz, 1H, Ar-H), 7.65 (dd, *J* = 8.5, 1.9 Hz, 1H, Ar-H), 7.07 (d, *J* = 1.8 Hz, 1H, Ar-H), 7.03 (d, *J* = 1.8 Hz, 1H, Ar-H), 5.44 (s, 2H, OCH_2O), 3.60 (s, 3H, OCH_3), 1.64–1.58 (m, 2H, CH_2), 1.31 (s, 6H, 2 × CH_3), 1.27–1.17 (m, 6H, 3 × CH_2), 1.10–1.02 (m, 2H, CH_2), 0.84 (t, *J* = 6.8 Hz, 3H, CH_3). ^{13}C NMR (126 MHz, $CDCl_3$) δ 160.9, 155.5, 154.1, 152.4, 135.9, 131.6, 130.8, 130.2, 129.9, 119.5, 109.0, 108.4, 105.1, 95.2, 56.7, 44.2, 38.3, 31.7, 29.9, 28.7, 24.6, 22.6, 14.1. Mass spectrum (ESI) m/z (relative intensity) 461 (M^+ + H, 65), 201 (100). LC/MS analysis (Waters MicroMass ZQ system) showed retention time 6.3 min for the title compound.

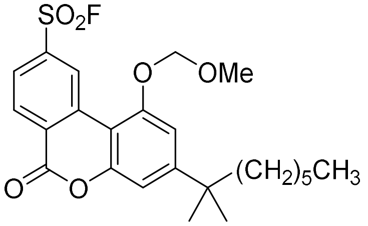

1-(Methoxymethoxy)-3-(2-methyloctan-2-yl)-6-oxo-6*H*-benzo[*c*]chromene-9-sulfonyl fluoride (57). To a mixture of **56** (92 mg, 0.2 mmol), DABSO (52 mg, 0.22 mmol), and $PdCl_2(Amphos)_2$ (16 mg, 0.02 mmol) under an argon atmosphere in a resealable tube at room temperature was added anhydrous Et_3N (60 mg, 0.6 mmol) and anhydrous isopropanol (1.0 mL). The resealable tube was sealed, and the reaction mixture was stirred at 75 ºC for 24 h. The reaction mixture was cooled to room temperature, NFSI (94 mg, 0.3 mmol) was added in one portion, and the reaction mixture was stirred at room temperature for 3 h. Solvent was removed under reduced pressure, and the mixture was diluted with EtOAc and then filtered through a Celite pad. The filtrate was washed with saturated aqueous $Na_2S_2O_3$ and brine, dried over $MgSO_4$, filtered, and concentrated under reduced pressure. The residue was purified by silica gel column chromatography with EtOAc/hexane (0/1 → 1/19) as eluent to afford **57** (66 mg, 71%) as a white solid. Mp 57−58 ºC. IR (thin film, cm^{-1}) 2963, 2930, 2860, 1742 (CO), 1618, 1416, 1276, 1261, 1211, 1079, 1049. ^1H NMR (500 MHz, $CDCl_3$) δ 9.72 (d, *J* = 1.8 Hz, 1H, Ar-H), 8.63 (d, *J* = 8.3 Hz, 1H, Ar-H), 8.07 (dd, *J* = 8.3, 1.8 Hz, 1H, Ar-H), 7.11 (d, *J* = 1.8 Hz, 1H, Ar-H), 7.06 (d, *J* = 1.8 Hz, 1H, Ar-H), 5.48 (s, 2H, OCH_2O), 3.61 (s, 3H, OCH_3), 1.67–1.59 (m, 2H, CH_2), 1.33 (s, 6H, 2 × CH_3), 1.27–1.17 (m, 6H, 3 × CH_2), 1.11–1.01 (m, 2H, CH_2), 0.83 (t, *J* = 6.7 Hz, 3H, CH_3). ^{13}C NMR (126 MHz, $CDCl_3$) δ 159.6, 155.6, 155.6, 152.4, 138.2 (d, $^2J_{CF}$ = 25.3 Hz), 135.8, 131.7, 127.6, 125.7, 125.6, 109.0, 108.5, 104.4, 95.2, 56.9, 44.1, 38.5, 31.7, 29.8, 28.6, 24.6, 22.6, 14.0; ^{19}F NMR (470 MHz, $CDCl_3$) δ 65.2.

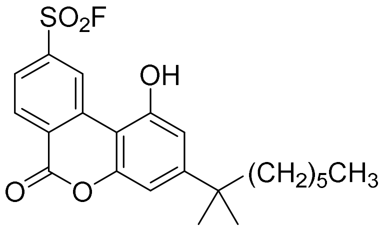

1-Hydroxy-3-(2-methyloctan-2-yl)-6-oxo-6*H*-benzo[*c*]chromene-9-sulfonyl fluoride (58). To a mixture of **57** (40 mg, 0.086 mmol) and ethanol (396 mg, 8.60 mmol) in CH_3CN (10 mL) at room temperature was added

$Sc(OTf)_3$ (30 mg, 0.061 mmol). After being flushed with argon, the reaction mixture was refluxed until most of the starting material was consumed as judged by TLC (ca. 8 h). CH_3CN was removed under reduced pressure, and water (ca. 0.5 mL) was added and stirred for 5 min. The organic material was extracted with EtOAc, and the combined organic extracts were concentrated under reduced pressure. The residue was purified by silica gel column chromatography with EtOAc/hexane (0/1 → 1/9) as eluent to afford **58** (33 mg, 91%) as a pale yellow solid. Mp 216–217 ºC. IR (thin film, cm^{-1}) 3373 (br, OH), 2968, 2933, 2859, 1707 (CO), 1630, 1585, 1472, 1418, 1397, 1300, 1213, 1118, 1093, 1045. ^1H NMR (500 MHz, CD_3CN) δ 9.71 (d, J = 1.8 Hz, 1H, Ar-H), 8.57 (d, J = 8.5 Hz, 1H, Ar-H), 8.55 (s, 1H, OH), 8.13 (dd, J = 8.5, 1.8 Hz, 1H, Ar-H), 6.99 (d, J = 1.8 Hz, 1H, Ar-H), 6.96 (d, J = 1.8 Hz, 1H, Ar-H), 1.70–1.63 (m, 2H, CH_2), 1.33 (s, 6H, 2 × CH_3), 1.30–1.20 (m, 6H, 3 × CH_2), 1.15–1.05 (m, 2H, CH_2), 0.86 (t, J = 6.7 Hz, 3H, CH_3). ^{13}C NMR (100 MHz, CD_3CN) δ 160.7, 156.6, 156.3, 153.8, 138.4 (d, $^2J_{CF}$ = 25.0 Hz), 137.0, 132.4, 128.3, 127.1, 126.9, 111.2, 107.9, 103.8, 44.7, 39.0, 32.5, 30.6, 28.9, 25.4, 23.3, 14.3; ^{19}F NMR (376 MHz, CD_3CN) δ 64.5. HRMS (ESI) for $C_{22}H_{26}O_5SF$: calculated 421.1485; found 421.1503.

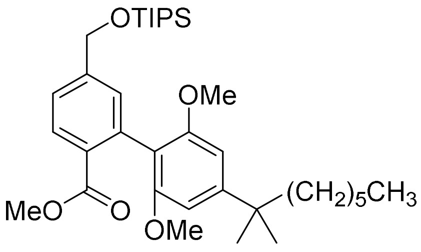

Methyl 2',6'-dimethoxy-4'-(2-methyloctan-2-yl)-5-(((triisopropylsilyl)oxy)methyl)-[1,1'-biphenyl]-2-carboxylate (59). The synthesis was carried out as described for **15**, using boronic acid **13** (501 mg, 1.63 mmol), **37** (548 mg, 1.36 mmol), Cs_2CO_3 (1.63 g, 5 mmol) and $Pd(PPh_3)_4$ (162 mg, 0.14 mmol) in DME/H_2O (5:1, 5 mL of DME and 1 mL of H_2O). The reaction was completed in 45 min and the crude oil obtained after work up was purified by flash column chromatography on silica gel (10-25% EtOAc/hexane) to give **59** (684 mg, 86 % yield) as a yellow oil. IR (thin film, cm^{-1}) 2925, 2863, 1707 (CO), 1609, 1576, 1461, 1283, 1239, 1126, 881, 806; ^1H NMR (500 MHz, $CDCl_3$) δ 7.89 (d, J = 8.0 Hz, 1H, Ar-H), 7.36 (d, J = 8.0 Hz, 1H, Ar-H), 7.28 (s, 1H, Ar-H), 6.55 (s, 2H, Ar-H), 4.86 (s, 2H), 3.66 (s, 6H, $-OCH_3$), 3.55 (s, 3H, $-COOCH_3$), 1.64 – 1.57 (m, 2H), 1.32 (s, 6H, $-CH_3$), 1.27 – 1.11 (m, 11H), 1.07 (d, J = 6.7 Hz, 18H), 0.84 (t, J = 6.9 Hz, 3H). ^{13}C NMR (100 MHz, $CDCl_3$) δ 168.5, 156.6, 150.8, 144.7, 134.8, 130.3, 129.9, 129.7, 124.1, 116.5, 102.2, 64.7, 55.8, 51.3, 44.7, 38.2, 31.8, 30.0, 28.9, 24.7, 22.6, 18.0, 14.0, 12.0.

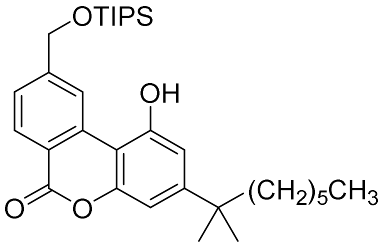

1-Hydroxy-3-(2-methyloctan-2-yl)-9-(((triisopropylsilyl)oxy)methyl)-6H-benzo[c]chromen-6-one (60). The synthesis was carried out as described for **1a**, using **59** (462 mg, 0.8 mmol), 9-iodo-9-BBN (2.6 mL of a 1.0 M in hexane, 2.6 mmol) in anhydrous CH_2Cl_2 (8 mL). The reaction was quenched by ethanolamine (1 mL) and the crude obtained after work up was purified by flash column chromatography on silica gel (CH_2Cl_2/Et_2O/hexane, 2:2:6) to give **60** (252 mg, 60 % yield) as a white solid. IR (thin film, cm^{-1}) 3332 (OH), 2927, 2866, 1722 (CO), 1708, 1270, 1074,883, 688; ^1H NMR (500 MHz, CDCl3) δ 9.00 (s, 1H, Ar-H), 8.36 (d, J = 8.1 Hz, 1H, Ar-H), 7.47 (d, J = 8.1 Hz, 1H, Ar-H), 6.95 (d, J = 1.4 Hz, 1H, Ar-H), 6.66 (d, J = 1.4 Hz, 1H, Ar-H), 5.58 (s, 1H, OH), 5.00 (s, 2H), 1.57 (m, 2H), 1.28 (s, 6H, $-CH_3$), 1.26 – 1.14 (m, 11H), 1.11 (d, J = 6.7 Hz, 18H), 0.82 (t, J = 6.9

Hz, 3H). ^{13}C NMR (100 MHz, $CDCl_3$) δ 161.4, 153.8, 153.3, 152.6, 144.7, 135.3, 130.6, 127.3, 127.1, 120.5, 110.1, 107.8, 104.1, 65.7, 44.3, 38.0, 32.6, 31.7, 29.9, 28.6, 24.6, 22.6, 18.0, 14.0.

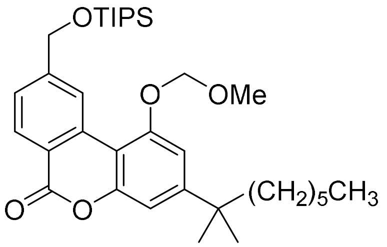

1-(Methoxymethoxy)-3-(2-methyloctan-2-yl)-9-(((triisopropylsilyl)oxy)methyl)-6H-benzo[c]chromen-6-one (61). The synthesis was carried out as described for **59**, using **60** (250 mg, 0.47 mmol), *N,N*-diisopropylethylamine (250 µL, 1.43 mmol), chloromethyl methyl ether (110 µL, 1.43 mmol) in anhydrous CH_2Cl_2 (4.8 mL). The crude oil obtained after work up was purified by flash column chromatography on silica gel (10% MeOH/CH_2Cl_2) to give title compound (265 mg, 98 % yield) as a colorless oil. IR (thin film, cm^{-1}) 2928, 2865, 1731 (CO), 1614, 1265, 1099, 1052, 967, 741, 701, 682; ^{1}H NMR (500 MHz, $CDCl_3$) δ 8.97 (d, *J* = 0.8 Hz, 1H, Ar-H), 8.38 (d, *J* = 8.1 Hz, 1H, Ar-H), 7.48 (dd, *J* = 8.1, 1.8 Hz, 1H, Ar-H), 7.11 (d, *J* = 1.8 Hz, 1H, Ar-H), 7.02 (d, *J* = 1.8 Hz, 1H, Ar-H), 5.40 (s, 2H), 4.98 (s, 2H), 3.53 (s, 3H, -OCH_3), 1.62 – 1.58 (m, 2H), 1.30 (s, 6H), 1.26 – 1.15 (m, 6H), 1.12 (s, 10H), 1.11 (s, 8H), 1.08 – 1.02 (m, 2H), 0.82 (t, *J* = 7.0 Hz, 3H). ^{13}C NMR (100 MHz, $CDCl_3$) δ 161.6, 155.7, 153.0, 152.3, 148.8, 134.7, 130.2, 125.3, 123.8, 119.6, 109.1, 108.7, 106.5, 95.0, 65.2, 56.3, 44.2, 38.2, 31.7, 29.9, 28.7, 24.7, 22.6, 18.1, 14.0, 12.0.

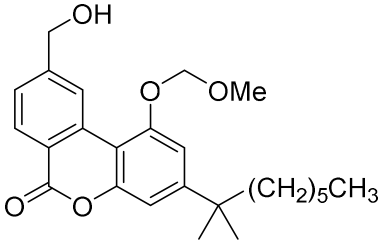

9-(Hydroxymethyl)-1-(methoxymethoxy)-3-(2-methyloctan-2-yl)-6H-benzo[c]chromen-6-one (62). To a solution of 1-(methoxymethoxy)-3-(2-methyloctan-2-yl)-9-(((triisopropylsilyl)oxy)methyl)-6H-benzo[c]chromen-6-one (53 mg, 0.09 mmol) in anhydrous THF (2.5 mL) at 0 ºC under an argon atmosphere was added TBAF (0.11 mL, 1M in THF, 0.11 mmol). The reaction was kept at 0 ºC for 10 min and then at room temperature for 1.5 h. The mixture was quenched by the addition of saturated aqueous NH_4Cl, extracted with Et_2O and the combined organic extracts were washed with brine, dried over $MgSO_4$ and concentrated under reduced pressure. Purification by flash column chromatography on silica gel (20-40 % EtOAc/hexane) gave **62** (37 mg, 97 % yield) as a yellow oil. IR (thin film, cm^{-1}) 3440 (OH), 2927, 1706 (CO), 1612, 1266, 1054, 750; ^{1}H NMR (500 MHz, $CDCl_3$) δ 8.98 (s, 1H), 8.39 (d, *J* = 8.1 Hz, 1H, Ar-H), 7.51 (d, *J* = 8.1 Hz, 1H, Ar-H), 7.03 (d, *J* = 7.8 Hz, 2H, Ar-H), 5.41 (s, 2H), 5.28 (s, 1H), 4.88 (s, 2H), 3.57 (s, 3H, -OCH_3), 1.65 – 1.53 (m, 2H), 1.29 (s, 6H, -CH_3), 1.26 – 1.13 (m, 6H), 1.05 (m, 2H), 0.82 (t, *J* = 6.8 Hz, 3H). ^{13}C NMR (100 MHz, $CDCl_3$) δ 161.5, 155.6, 153.3, 152.4, 147.8, 134.9, 130.6, 125.9, 124.7, 120.1, 109.1, 108.5, 106.3, 95.3, 65.2, 56.7, 44.2, 38.3, 31.7, 29.9, 28.7, 24.6, 17.7, 14.0.

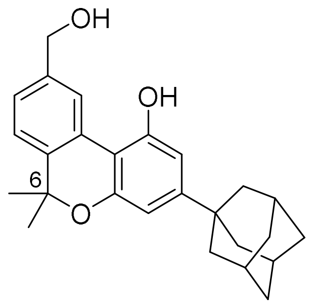

3-(Adamantan-1-yl)-9-(hydroxymethyl)-6,6-dimethyl-6*H*-benzo[*c*]chromen-1-ol (63).[6] To a solution of **39** (400 mg, 0.75 mmol) in THF (25 mL) under an argon atmosphere at room temperature was slowly added a 3.0 M solution of methylmagnesium bromide in Et_2O (2.5 mL, 7.5 mmol). The reaction mixture was stirred at room temperature for 30 min and then refluxed for an additional 1.5 h. After the mixture was cooled to room temperature, saturated aqueous NH_4Cl (ca. 20 mL) was added, and the organic material was extracted with Et_2O. The combined organic extracts were washed with brine, dried over $MgSO_4$, filtered, and concentrated under reduced pressure to give the crude intermediate as a white foam that was used without further purification in the subsequent cyclization. The crude was dissolved in $CHCl_3$ (10 mL) at room temperature, and *p*-TsOH·H_2O (43 mg, 0.225 mmol) was added. After being flushed with argon, the reaction mixture was stirred at room temperature for 12 h. Water was added, and the organic material was extracted with $CHCl_3$. The combined organic extracts were washed with saturated aqueous $NaHCO_3$ and water, dried over $MgSO_4$, filtered, and concentrated under reduced pressure. The residue was purified by silica gel column chromatography with EtOAc/hexane (1/10 → 1/1) as eluent to afford **63** (190 mg, 65%) as a white solid. Mp 118–119 ºC. IR (thin film, cm^{-1}) 3348 (br, OH), 2904, 2849, 1621, 1584, 1407, 1264, 1055. ^1H NMR (500 MHz, CD_3OD) δ 8.56 (s, 1H, Ar-H), 7.22–7.19 (m, 2H, Ar-H), 6.56 (d, J = 1.9 Hz, 1H, Ar-H), 6.45 (d, J = 1.9 Hz, 1H, Ar-H), 4.61 (s, 2H, CH_2), 2.05–2.03 (m, 3H, Ad-H), 1.89–1.88 (m, 6H, Ad-H), 1.81–1.73 (m, 6H, Ad-H), 1.54 (s, 6H, 2 × CH_3). ^{13}C NMR (126 MHz, CD_3OD) δ 156.3, 155.4, 154.2, 141.0, 139.7, 129.6, 126.5, 126.3, 123.4, 109.3, 107.2, 106.9, 78.0, 65.5, 44.1, 37.9, 37.1, 30.3, 27.5. HRMS (ESI) for $C_{26}H_{31}O_3$: calculated 391.2273; found 391.2266. Mass spectrum (ESI) m/z (relative intensity) 391 (M^+ + H, 100). LC/MS analysis (Waters MicroMass ZQ system) showed retention time 5.2 min for the title compound.

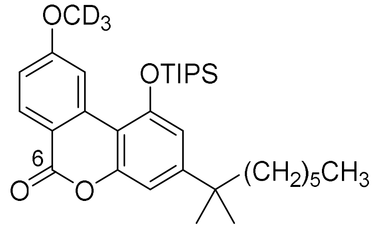

9-(Methoxy-d₃)-3-(2-methyloctan-2-yl)-1-((triisopropylsilyl)oxy)-6H-benzo[c]chromen-6-one (64). To a solution of **49** (156 mg, 0.42 mmol) in anhydrous CH_2Cl_2 (0.7 mL) under an argon atmosphere were added sequentially, 2,6-lutidine (400 mg, 3.6 mmol), and triisopropylsilyl trifluoromethanesulfonate (740 mg, 2.4 mmol) at 0 ºC. Following the addition, the reaction mixture was gradually warmed to room temperature and the stirring was continued at that temperature for 2 h and then quenched by the addition of saturated aqueous NH_4Cl and extracted with diethyl ether. The organic phase was dried ($MgSO_4$) and evaporated under reduced pressure. The residue was purified by silica gel column chromatography with EtOAc/hexane (1/10 → 2/10) as eluent to afford 197 mg (89% yield) of **64** as colorless viscous oil. IR (thin film, cm^{-1}) 2943, 2646, 2074 (CD), 1713 (CO), 1607, 1465, 1399, 1270, 1110, 883; ^1H-NMR ($CDCl_3$, 500 MHz) δ 8.49 (s, 1H, Ar-H), 8.35 (d, J = 7.2 Hz, 1H, Ar-H), 7.02 (d, J = 7.2 Hz, 1H, Ar-H), 9.92 (s, 1H, Ar-H), 6.76 (s 1H, Ar-H), 1.60-1.57 (m, 2H, -CH_2-), 1.48-1.42 (m, 3H, -CH-), 1.28 (s, 6H, -CH_3), 1.26-1.15 (m and d overlapping, 22H, 2x-CH_2- and -CH($C\underline{H}_3$)$_2$, especially 1.17, d, J = 7.1 Hz, -CH($C\underline{H}_3$)$_2$), 1.05-0.99 (m, 4H, -CH_2-), 0.81 (t, J = 6.9 Hz, 3H, -CH_3).

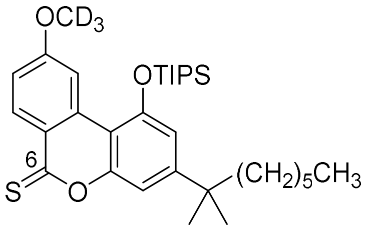

9-(Methoxy-d3)-3-(2-methyloctan-2-yl)-1-((triisopropylsilyl)oxy)-6H-benzo[c]chromene-6-thione (65). To a stirring solution of **64** (148 mg, 0.28 mmol) in anhydrous toluene (2 mL) under an argon atmosphere was added Lawesson reagent (226 mg, 0.56 mmol). The reaction mixture was refluxed at 120 ºC for 24 hours and then cooled to room temperature. Then the mixture was diluted with 1 mL solution of benzene/hexane (60:40) and stirred for another 30 minutes. Solid materials were filtered off and the filtrate was concentrated under reduced pressure. The residue was purified by silica gel column chromatography with EtOAc/hexane (1/10 $\rightarrow$ 2/10) as eluent to afford 115 mg (76% yield) of **65** as a yellow viscous oil. IR (thin film, cm^{-1}) 2928, 2868, 2073 (CD), 1605, 1463, 1397, 1279 and 1264 (CS), 1180, 1015, 883; ^{1}H-NMR ($CDCl_3$, 500 MHz) δ 8.89 (d, J = 7.2 Hz, 1H, Ar-H), 8.49 (s, 1H, Ar-H), 7.12 (s, 1H, Ar-H), 7.04 (d, J = 7.2 Hz, 1H, Ar-H), 6.81 (s, 1H, Ar-H), 1.59-1.55 (m, 2H, $-CH_2-$), 1.47-1.42 (m, 3H, -CH-), 1.26 (s, 6H, $-CH_3$), 1.23-1.15 (m and d overlapping, 22H, 2x$-CH_2-$ and $-CH(C\underline{H}_3)_2$, especially 1.18, d, J = 7.1 Hz, $-CH(C\underline{H}_3)_2$), 1.03-0.95 (m, 4H, $-CH_2-$), 0.81 (t, J = 6.9 Hz, 3H, $-CH_3$).

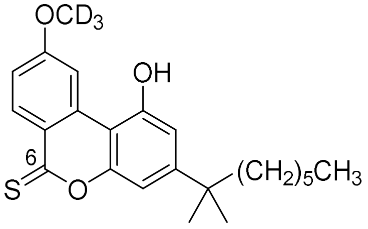

1-Hydroxy-9-(methoxy-d3)-3-(2-methyloctan-2-yl)-6H-benzo[c]chromene-6-thione (66). To a solution of **65** (27 mg, 0.05 mmol) in anhydrous THF (1.25 mL) at -40 °C, under an argon atmosphere, was added tetra-*n*-butylammonium fluoride (0.06 mL, 0.06 mmol, 1M solution in anhydrous THF). The reaction mixture was stirred for 30 min at the same temperature, and then quenched using a saturated aqueous NH_4Cl solution. Extractive isolation with diethyl ether, and purification by silica gel column chromatography with CH_2Cl_2/Et_2O/hexane (2:2:6) as eluent gave **66** (16 mg, 82% yield) as a yellow solid. Mp 120-126 ºC. IR (thin film, cm^{-1}) 3061 (OH), 2931, 2071 (CD), 1686, 1604, 1405, 1274, 1101, 740. ^{1}H-NMR ($CDCl_3$, 500 MHz) δ 8.88 (d, J = 7.2 Hz, 1H, Ar-H), 8.46 (d, J = 1.9 Hz, 1H, Ar-H), 7.14 (d, J = 1.4 Hz, 1H, Ar-H), 7.06 (dd, J = 7.2, 1.9 Hz, 1H, Ar-H), 6.70 (d, J = 1.4 Hz, 1H, Ar-H), 5.63 (brs, 1H, -OH), 1.61-1.58 (m, 2H, $-CH_2-$), 1.30 (s, 6H, $-CH_3$), 1.26-1.20 (m, 6H, $-CH_2-$), 1.05 (br s, 2H, $-CH_2-$), 0.84 (t, J = 6.9 Hz, 3H, $-CH_3$). ^{13}C-NMR ($CDCl_3$, 100 MHz) δ 199.5, 165.2, 154.4, 153.6, 153.4, 136.4, 132.3, 131.2, 123.4, 116.2, 110.4, 109.6, 108.2, 44.2, 38.0, 31.7, 29.9, 28.5, 24.6, 22.5, 13.9. HRMS (ESI) for $C_{23}H_{26}D_3O_3S$: calculated 388.2026; found 388.2025. Mass spectrum (ESI) m/z (relative intensity) 388 (M^+ + H, 100). LC/MS analysis (Waters MicroMass ZQ system) showed retention time 5.9 min for the title compound.

References

1. Papahatjis, D. P.; Nikas, S. P.; Kourouli, T.; Chari, R.; Xu, W.; Pertwee, R. G.; Makriyannis, A. *J. Med. Chem.* **2003**, *46*, 3221–3229.
2. Nikas, S. P.; Alapafuja, S. O.; Papanastasiou, I.; Paronis, C. A.; Shukla, V. G.; Papahatjis, D. P.; Bowman, A. L.; Halikhedkar, A.; Han, X. W.; Makriyannis, A. *J. Med. Chem.* **2010**, *53*, 6996–7010.
3. Makriyannis, A.; D'Souza, M. R.; Bajaj, S.; Nikas, S. P.; Thakur, G. A. Preparation of 2-cycloalkyl-resorcinol compounds as cannabinergic ligands. WO2014062965A1, 2014.
4. Ogawa, G.; Tius, M. A.; Zhou, H.; Nikas, S. P.; Halikhedkar, A.; Mallipeddi, S.; Makriyannis, A. *J. Med. Chem.* **2015**, *58*, 3104–3116.
5. Lu, D.; Meng, Z. X.; Thakur, G. A.; Fan, P. S.; Steed, J.; Tartal, C. L.; Hurst, D. P.; Reggio, P. H.; Deschamps, J. R.; Parrish, D. A.; George, C.; Jarbe, T. U. C.; Lamb, R. J.; Makriyannis, A. *J. Med. Chem.* **2005**, *48*, 4576–4585.
6. Thakur, G. A.; Bajaj, S.; Paronis, C.; Peng, Y.; Bowman, A. L.; Barak, L. S.; Caron, M. G.; Parrish, D.; Deschamps, J. R.; Makriyannis, A. *J. Med. Chem.* **2013**, *56*, 3904–3921.

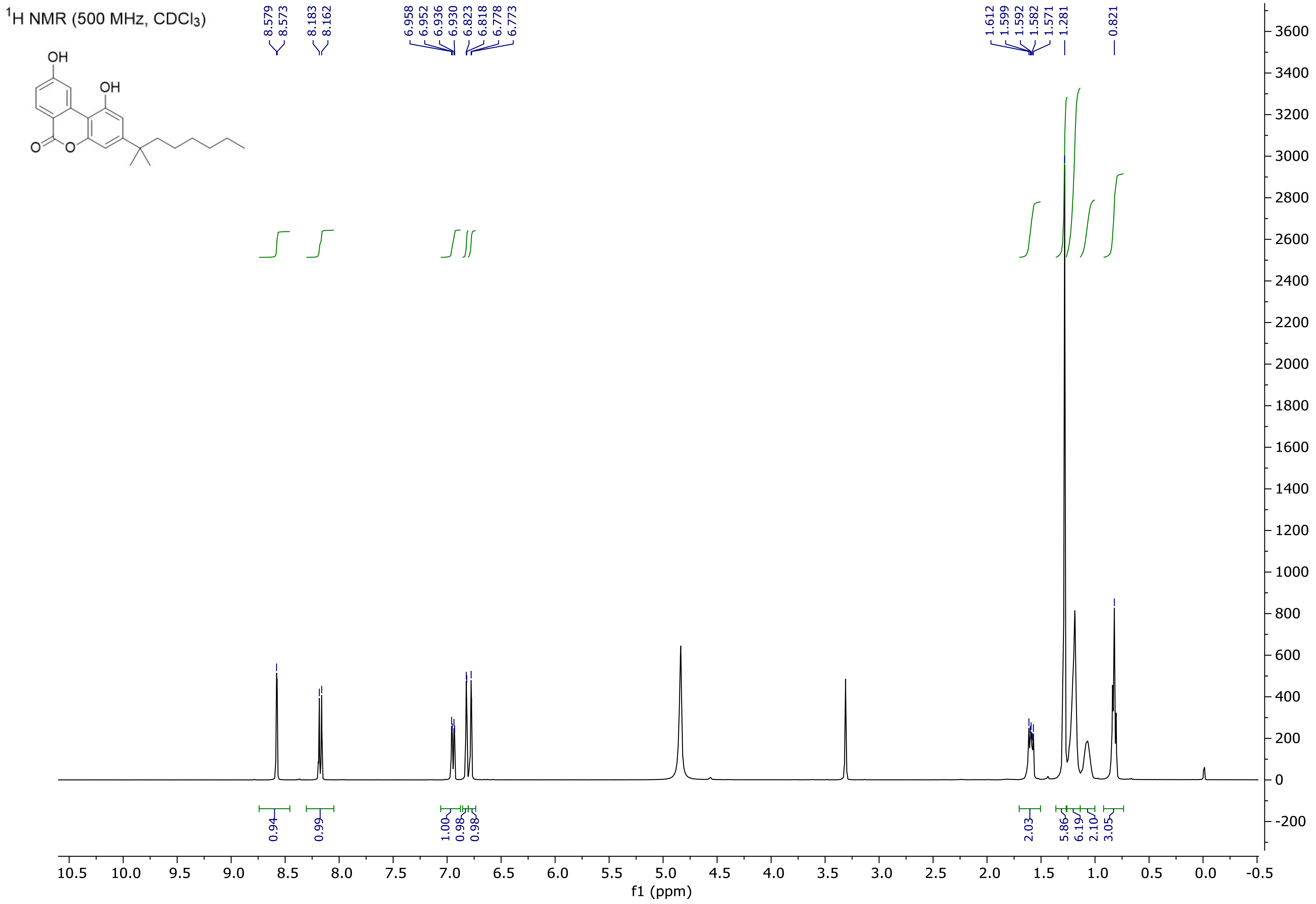

^{1}H NMR (500 MHz, CDCl3)
OH
OH
O
O
8.579
8.573
8.183
8.162
6.958
6.952
6.936
6.930
6.823
6.818
6.778
6.773
1.612
1.599
1.592
1.582
1.571
1.281
0.821
0.94
0.99
1.00
0.98
0.98
2.03
5.86
6.19
2.10
3.05
f1 (ppm)

^{13}C NMR (100 MHz, $CDCl_3$)

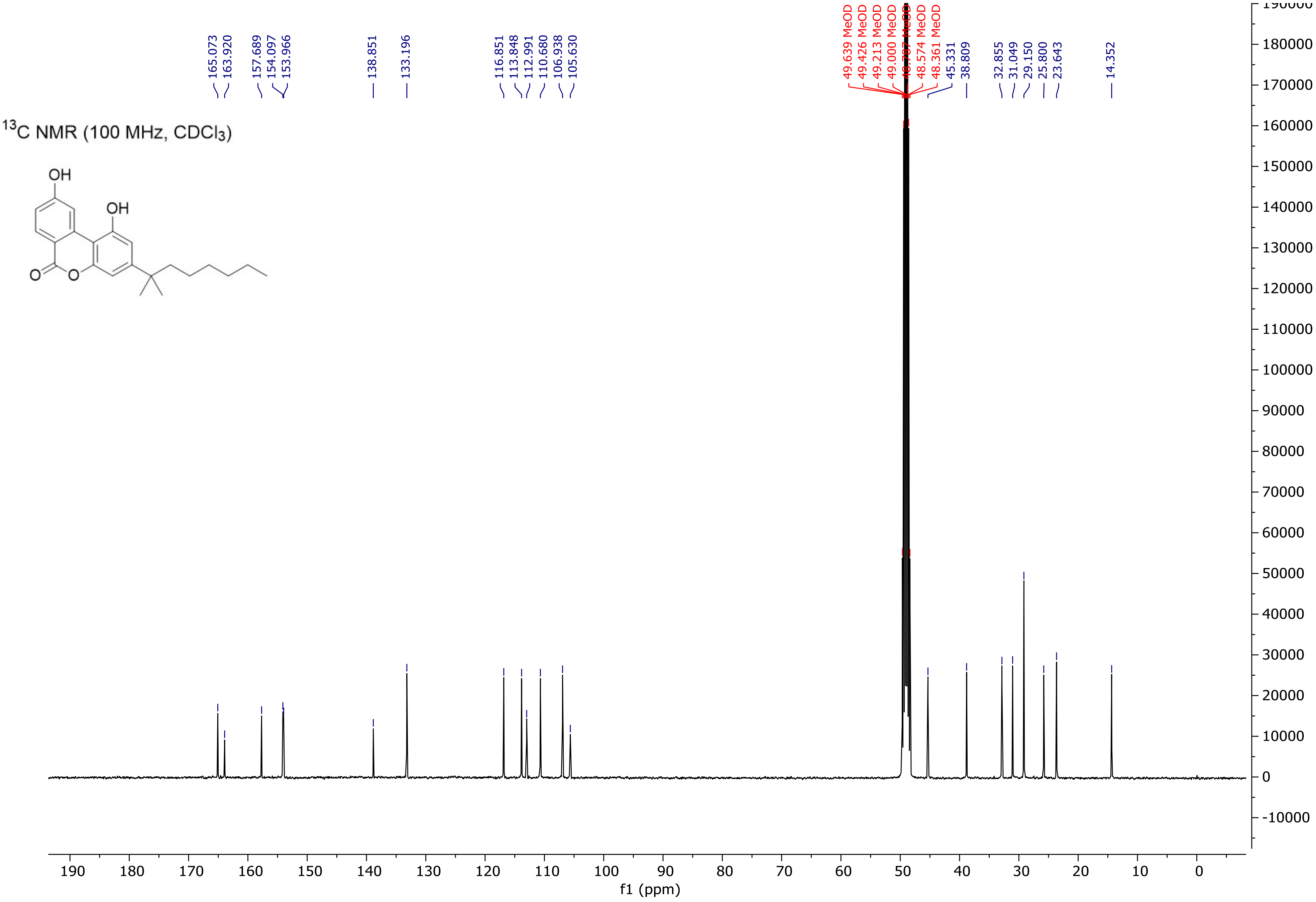

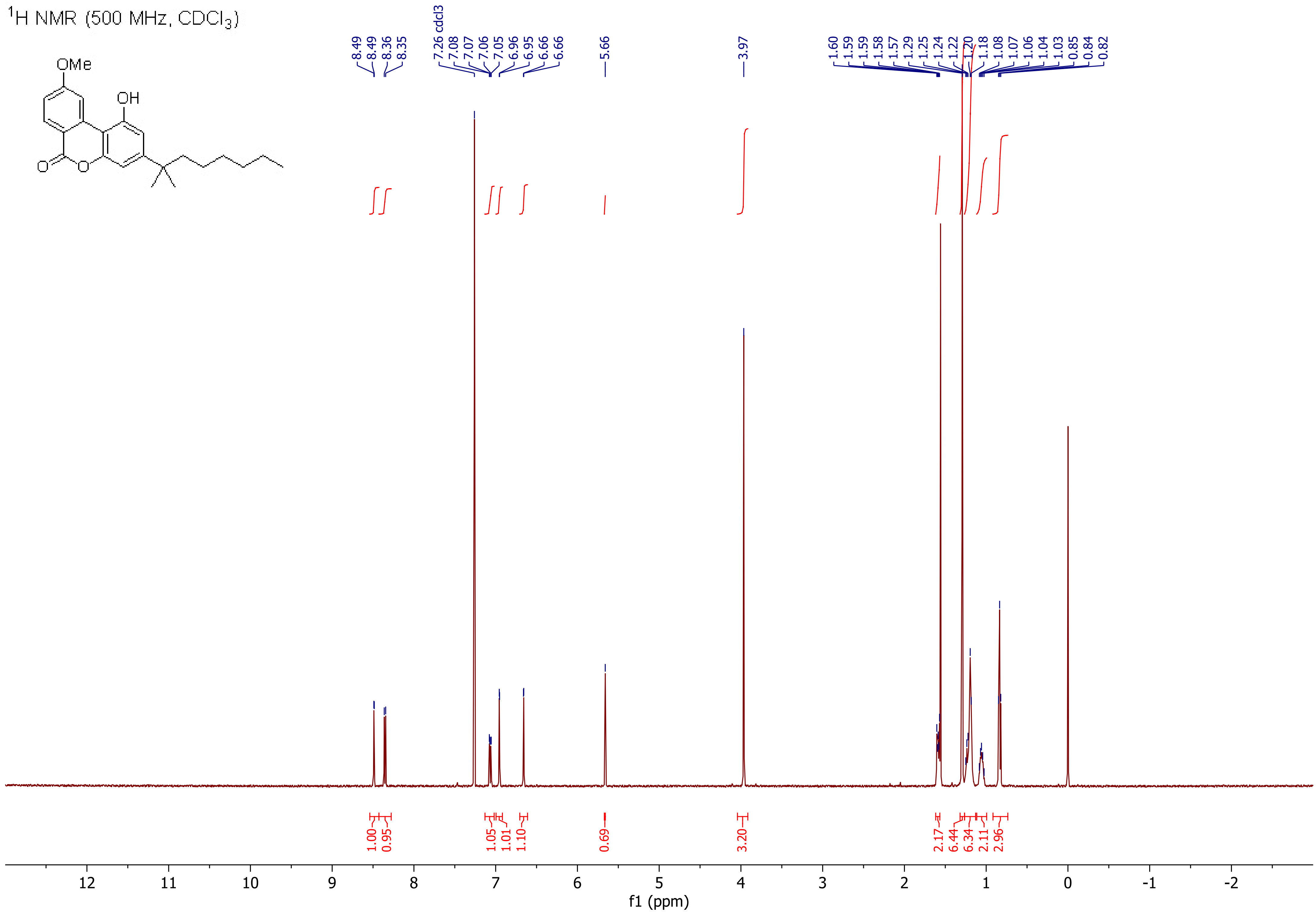

^{1}H NMR (500 MHz, CDCl$_3$)
OMe
OH
O
O
8.49
8.49
8.36
8.35
7.26 cdcl3
7.08
7.07
7.06
7.05
6.96
6.95
6.66
6.66
5.66
3.97
1.60
1.59
1.59
1.58
1.57
1.29
1.25
1.24
1.22
1.20
1.18
1.08
1.07
1.06
1.04
1.03
0.85
0.84
0.82
1.00
0.95
1.05
1.01
1.10
0.69
3.20
2.17
6.44
6.34
2.11
2.96
12
11
10
9
8
7
6
5
4
3
2
1
0
-1
-2
f1 (ppm)

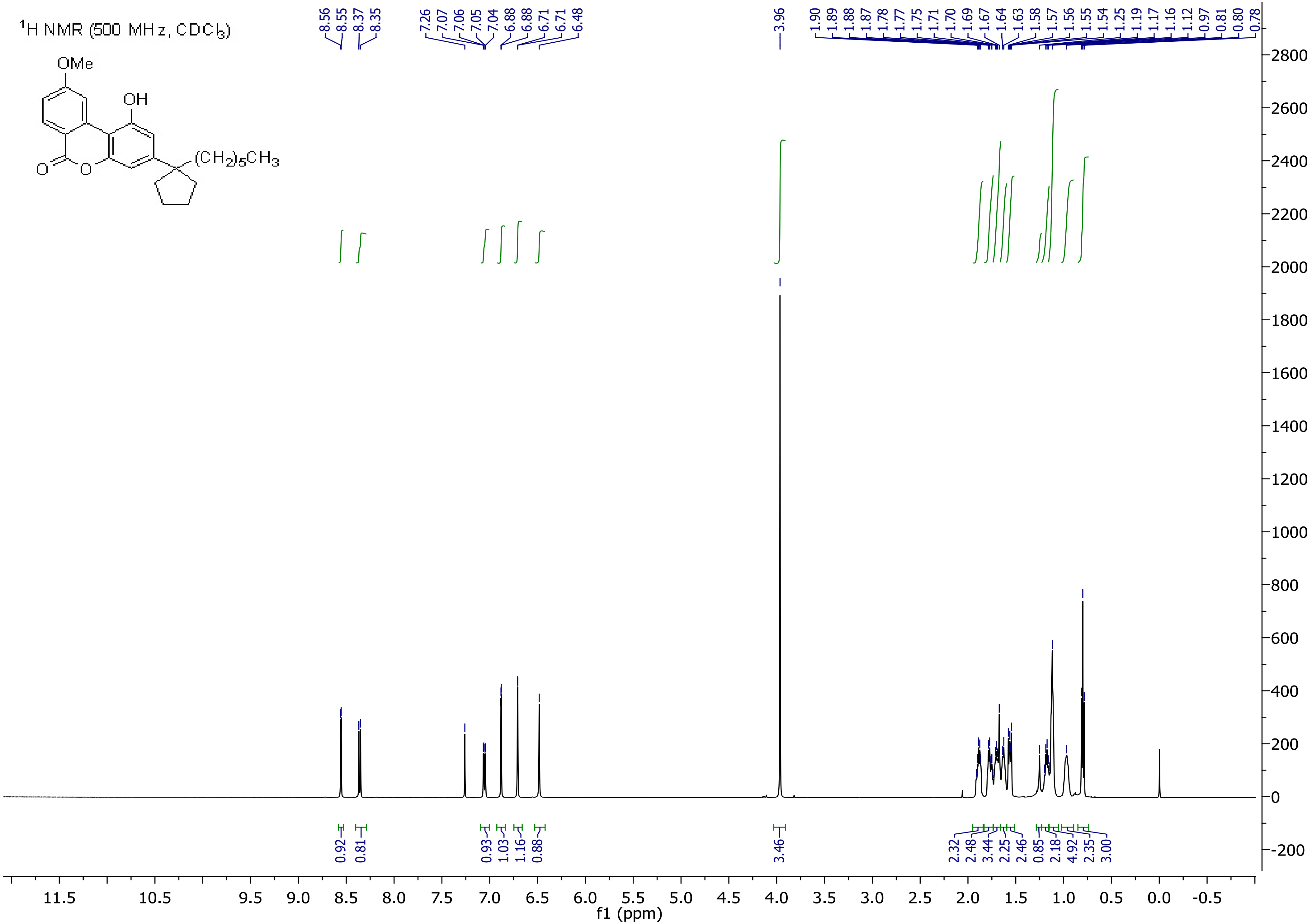

1H NMR (500 MHz, CDCl3)
OMe
OH
O
O
(CH2)5CH3
8.56
8.55
8.37
8.35
7.26
7.07
7.06
7.05
7.04
6.88
6.88
6.71
6.71
6.48
3.96
1.90
1.89
1.88
1.87
1.78
1.77
1.75
1.71
1.70
1.69
1.67
1.64
1.63
1.58
1.57
1.56
1.55
1.54
1.25
1.19
1.17
1.16
1.12
0.97
0.81
0.80
0.78
0.92
0.81
0.93
1.03
1.16
0.88
3.46
2.32
2.48
3.44
2.25
2.46
0.85
2.18
4.92
2.35
3.00
f1 (ppm)

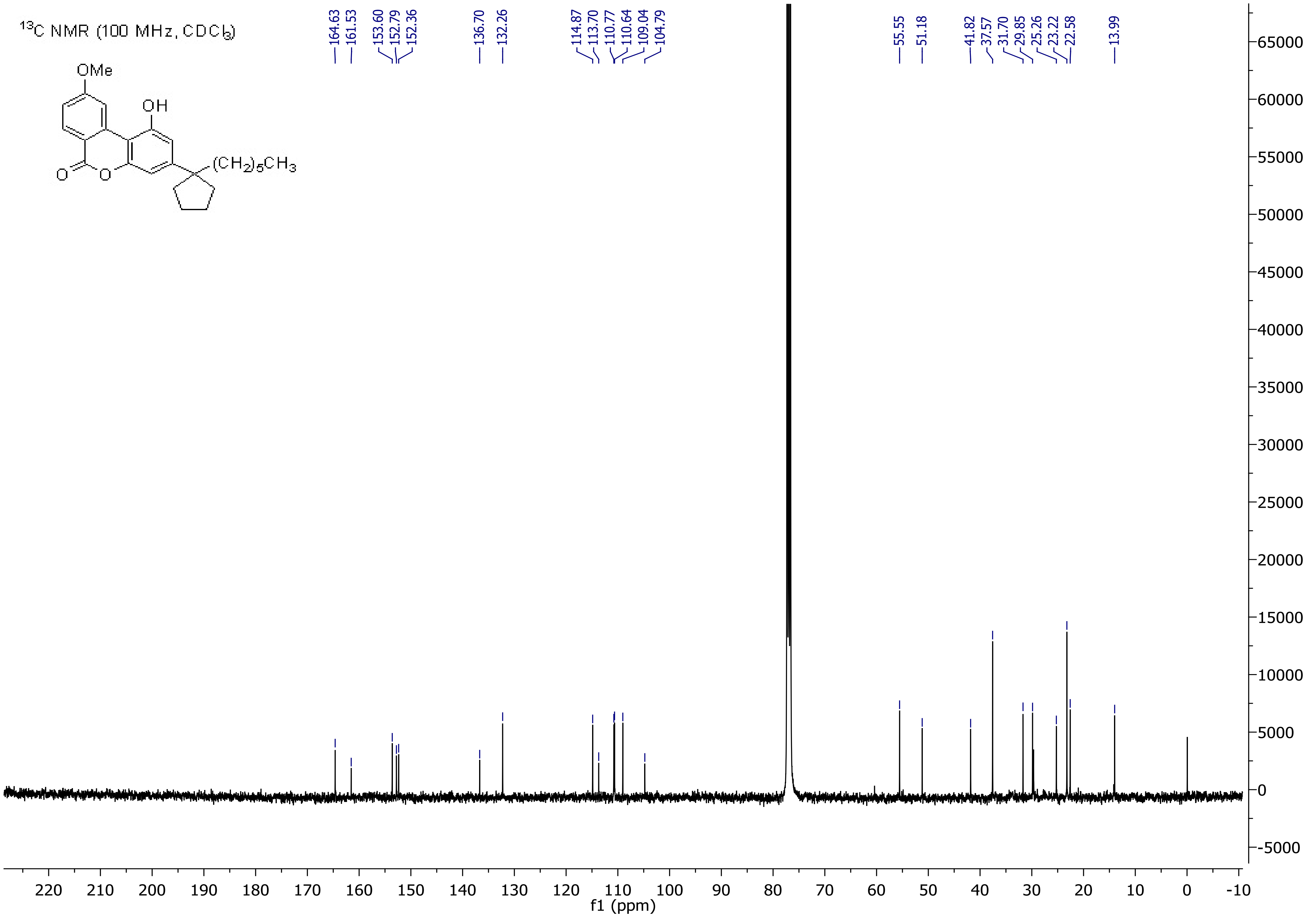

13C NMR (100 MHz, CDCl3)
OMe
OH
O
O
(CH2)5CH3
164.63
161.53
153.60
152.79
152.36
136.70
132.26
114.87
113.70
110.77
110.64
109.04
104.79
55.55
51.18
41.82
37.57
31.70
29.85
25.26
23.22
22.58
13.99
65000
60000
55000
50000
45000
40000
35000
30000
25000
20000
15000
10000
5000
0
-5000
220 210 200 190 180 170 160 150 140 130 120 110 100 90 80 70 60 50 40 30 20 10 0 -10
f1 (ppm)

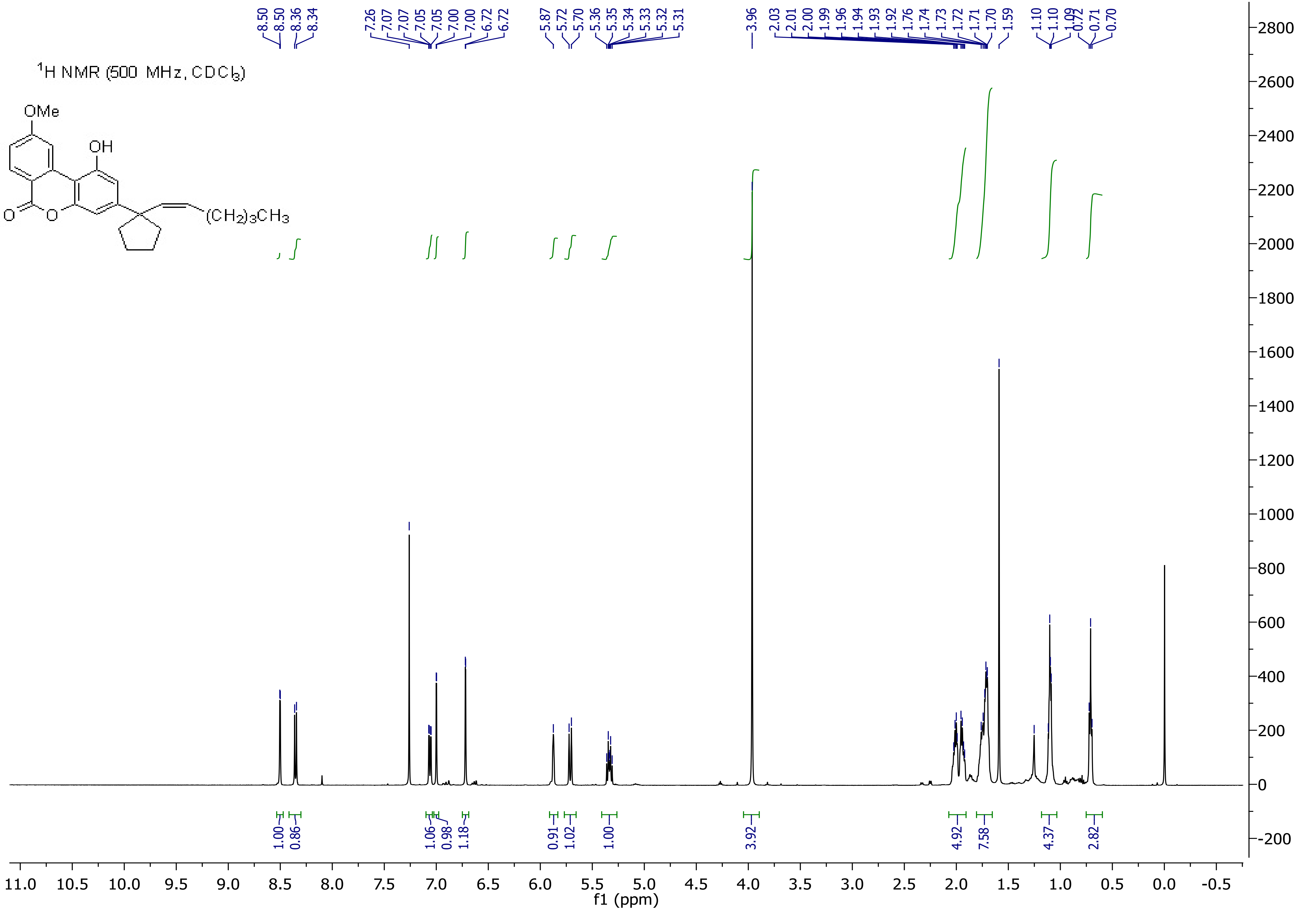

1H NMR (500 MHz, CDCl3)
OMe
OH
O
O
(CH2)3CH3
8.50 8.50 8.36 8.34 7.26 7.07 7.07 7.05 7.05 7.00 7.00 6.72 6.72 5.87 5.72 5.70 5.36 5.35 5.34 5.33 5.32 5.31 3.96 2.03 2.01 2.00 1.99 1.96 1.94 1.93 1.92 1.76 1.74 1.73 1.72 1.71 1.70 1.59 1.10 1.10 1.09 0.72 0.71 0.70
1.00 0.86 1.06 0.98 1.18 0.91 1.02 1.00 3.92 4.92 7.58 4.37 2.82
f1 (ppm)

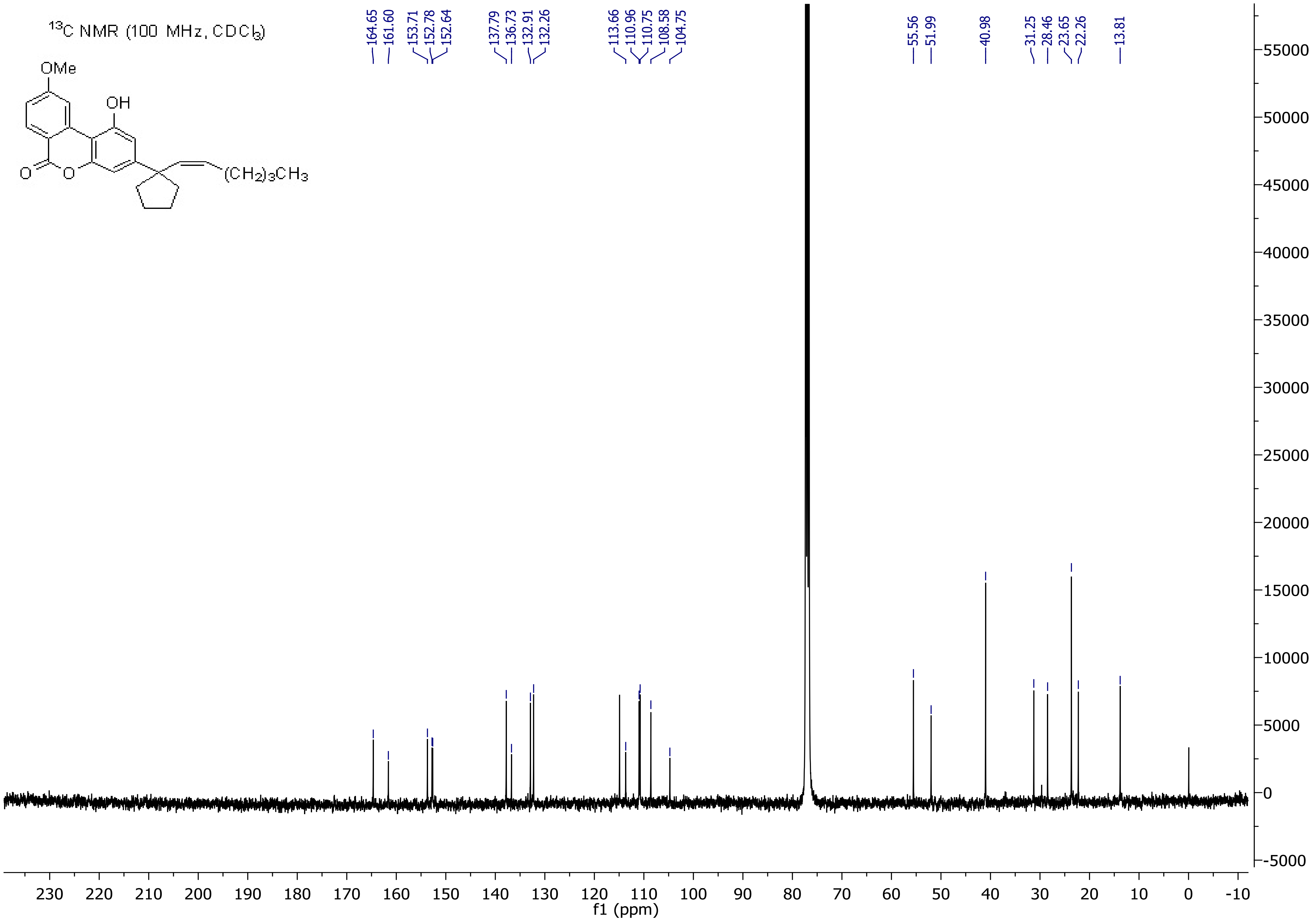

13C NMR (100 MHz, CDCl3)
OMe
OH
O
O
(CH2)3CH3
164.65
161.60
153.71
152.78
152.64
137.79
136.73
132.91
132.26
113.66
110.96
110.75
108.58
104.75
55.56
51.99
40.98
31.25
28.46
23.65
22.26
13.81
f1 (ppm)

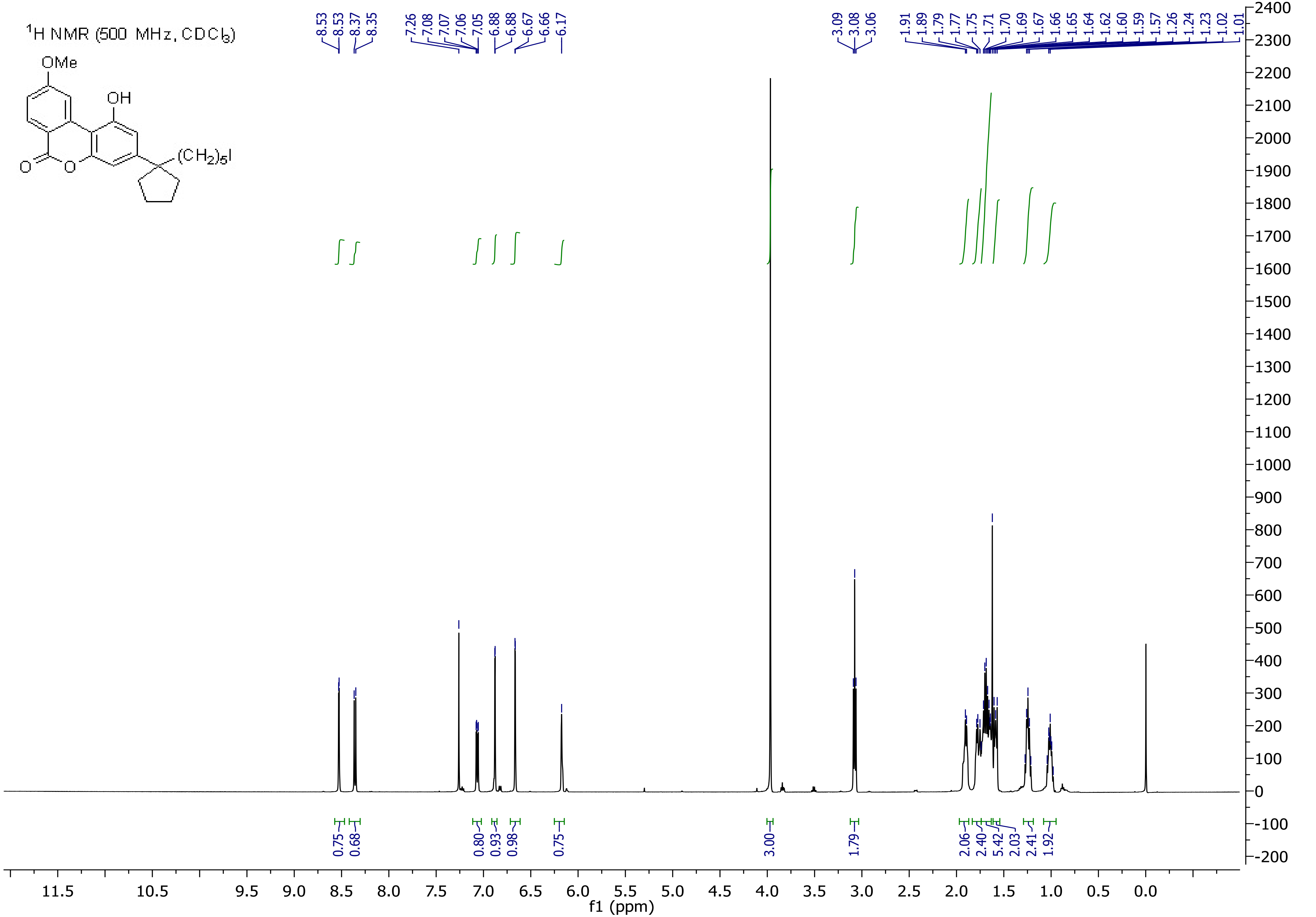

1H NMR (500 MHz, CDCl3)
OMe
OH
(CH2)5I
O
O
8.53
8.53
8.37
8.35
7.26
7.08
7.07
7.06
7.05
6.88
6.88
6.67
6.66
6.17
3.09
3.08
3.06
1.91
1.89
1.79
1.77
1.75
1.71
1.70
1.69
1.67
1.66
1.65
1.64
1.62
1.60
1.59
1.57
1.26
1.24
1.23
1.02
1.01
0.75
0.68
0.80
0.93
0.98
0.75
3.00
1.79
2.06
2.40
5.42
2.03
2.41
1.92
f1 (ppm)

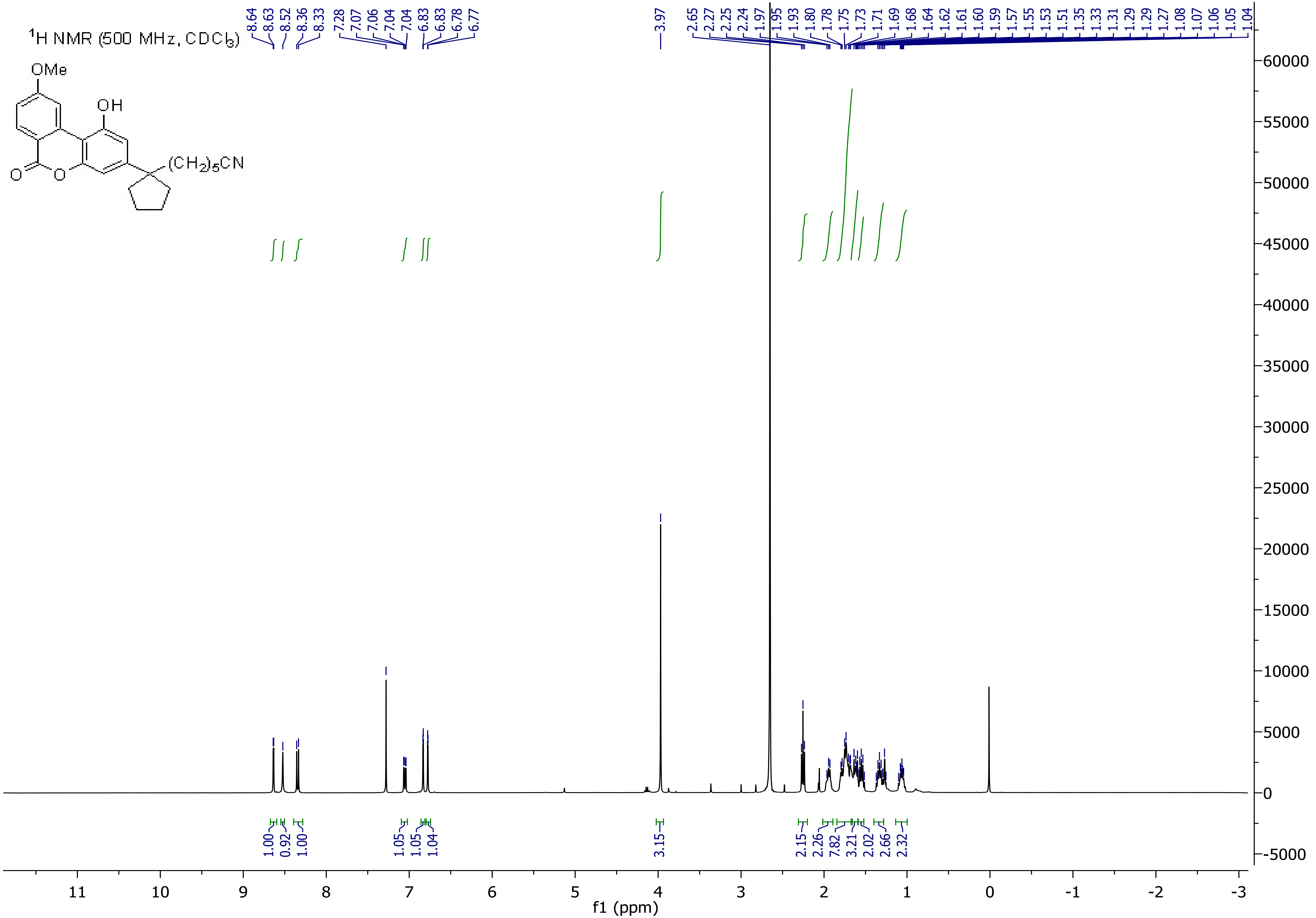

1H NMR (500 MHz, CDCl3)
OMe
OH
(CH2)5CN
O
O
8.64
8.63
8.52
8.36
8.33
7.28
7.07
7.06
7.04
7.04
6.83
6.83
6.78
6.77
3.97
2.65
2.27
2.25
2.24
1.97
1.95
1.93
1.80
1.78
1.75
1.73
1.71
1.69
1.68
1.64
1.62
1.61
1.60
1.59
1.57
1.55
1.53
1.51
1.35
1.33
1.31
1.29
1.29
1.27
1.08
1.07
1.06
1.05
1.04
1.00
0.92
1.00
1.05
1.05
1.04
3.15
2.15
2.26
7.82
3.21
2.02
2.66
2.32
f1 (ppm)

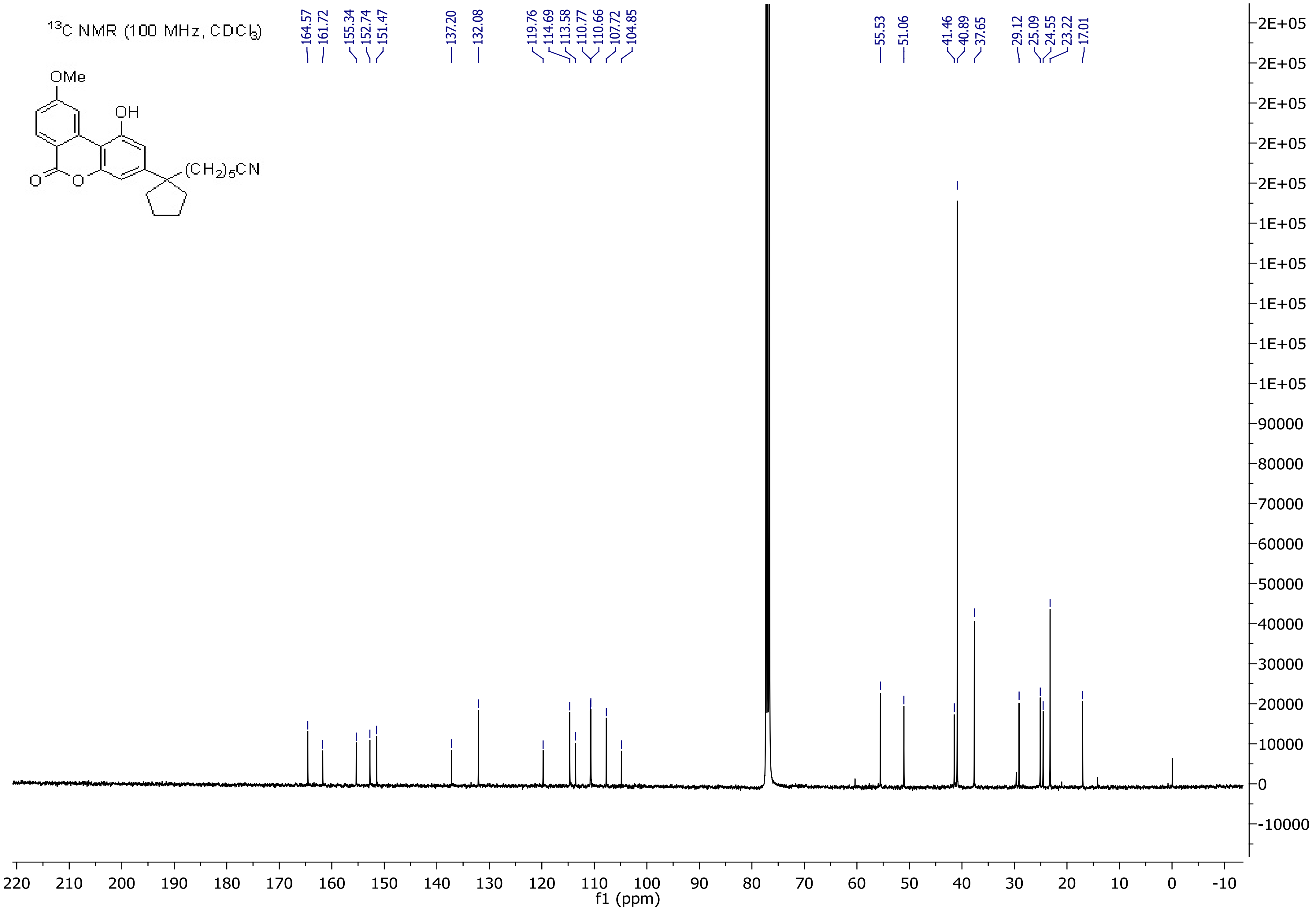

13C NMR (100 MHz, CDCl3)
OMe
OH
(CH2)5CN
O
O
164.57
161.72
155.34
152.74
151.47
137.20
132.08
119.76
114.69
113.58
110.77
110.66
107.72
104.85
55.53
51.06
41.46
40.89
37.65
29.12
25.09
24.55
23.22
17.01
f1 (ppm)

^1H NMR (500 MHz, $CDCl_3$)

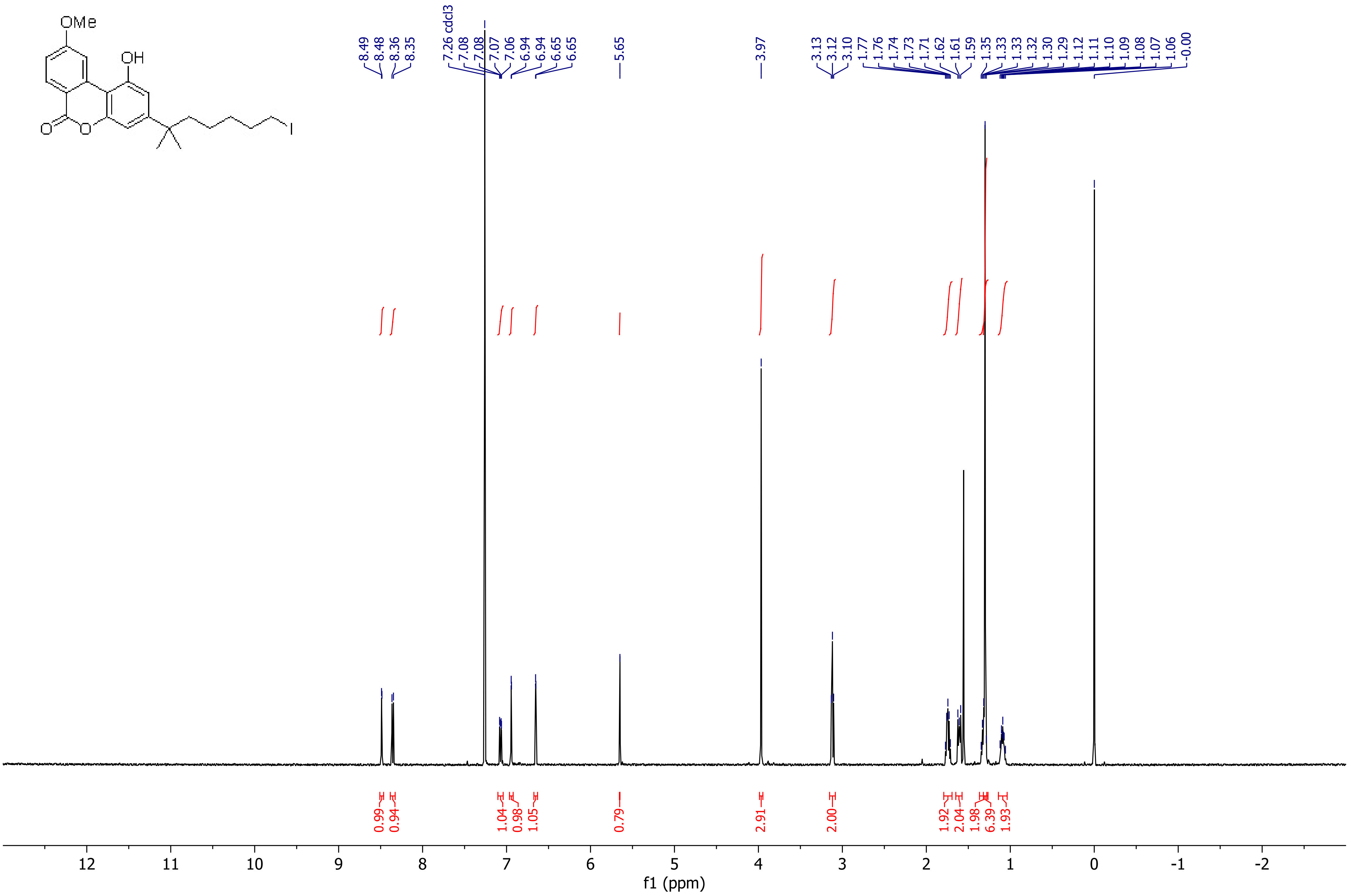

^{13}C NMR (126 MHz, $CDCl_3$)

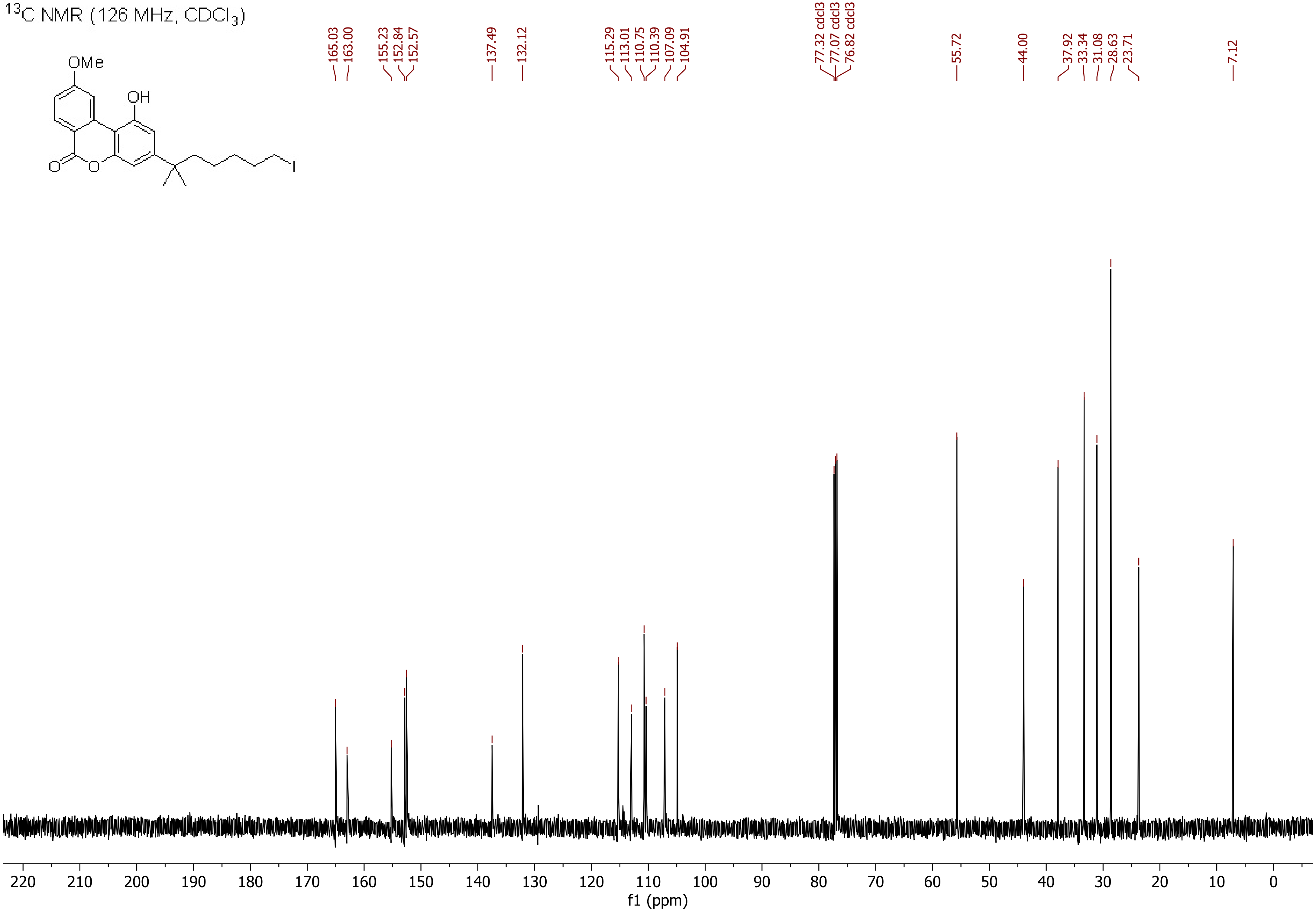

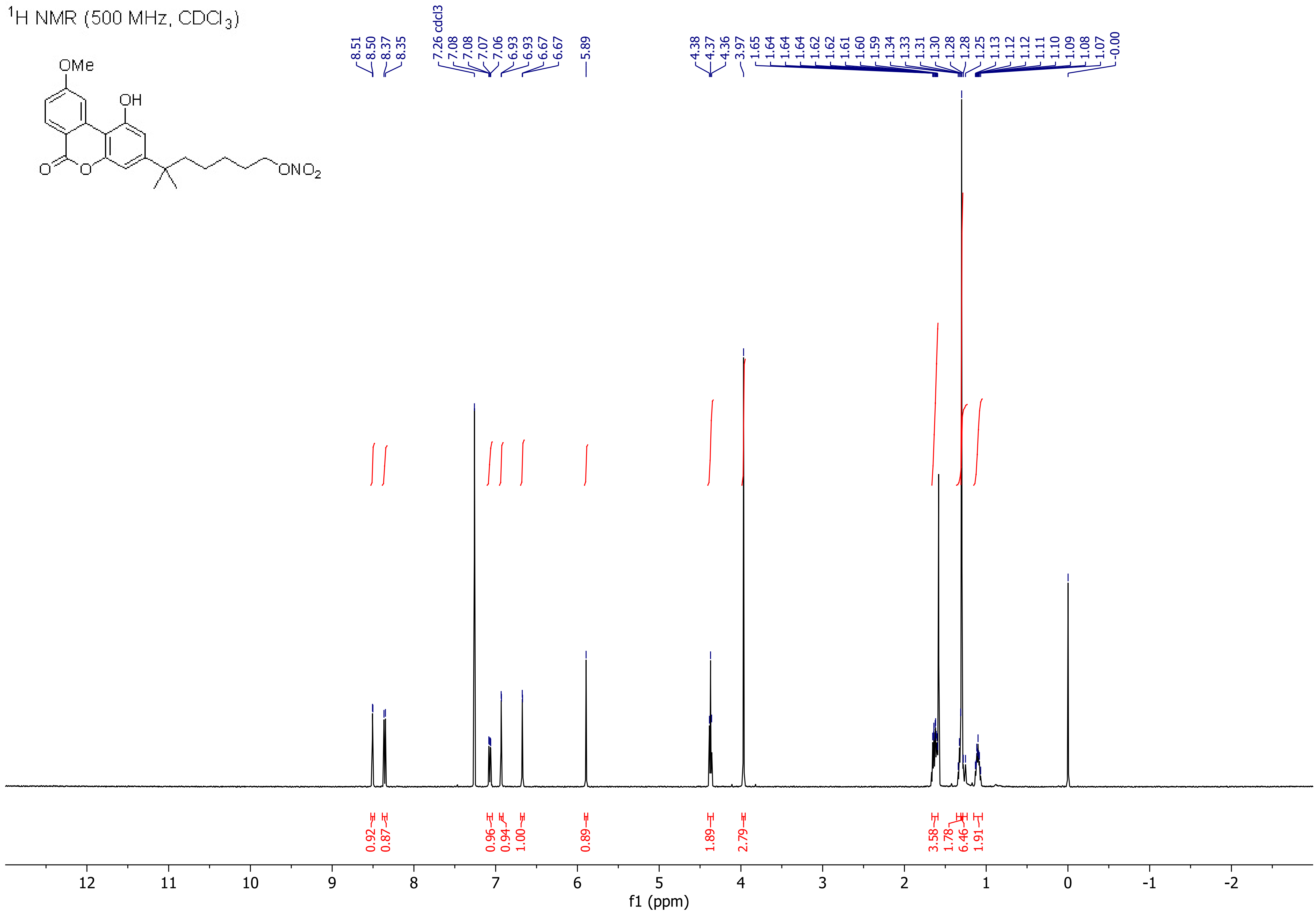

^{1}H NMR (500 MHz, CDCl$_3$)
OMe
OH
ONO$_2$
8.51
8.50
8.37
8.35
7.26 cdcl3
7.08
7.08
7.07
7.06
6.93
6.93
6.67
6.67
5.89
4.38
4.37
4.36
3.97
1.65
1.64
1.64
1.64
1.62
1.62
1.61
1.60
1.59
1.34
1.33
1.31
1.30
1.28
1.28
1.25
1.13
1.12
1.12
1.11
1.10
1.09
1.08
1.07
-0.00
0.92
0.87
0.96
0.94
1.00
0.89
1.89
2.79
3.58
1.78
6.46
1.91
12
11
10
9
8
7
6
5
4
3
2
1
0
-1
-2
f1 (ppm)

^{13}C NMR (126 MHz, $CDCl_3$)

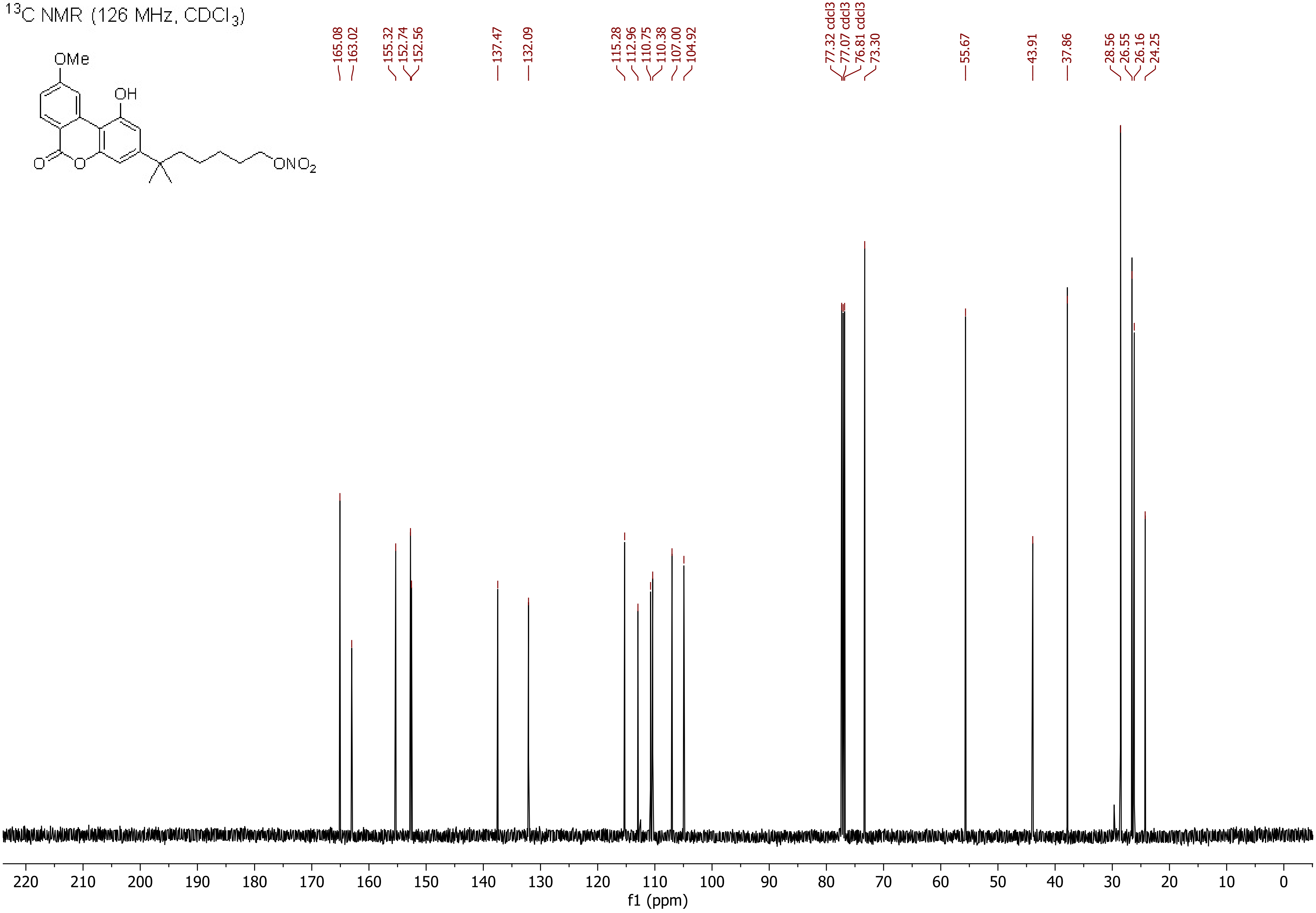

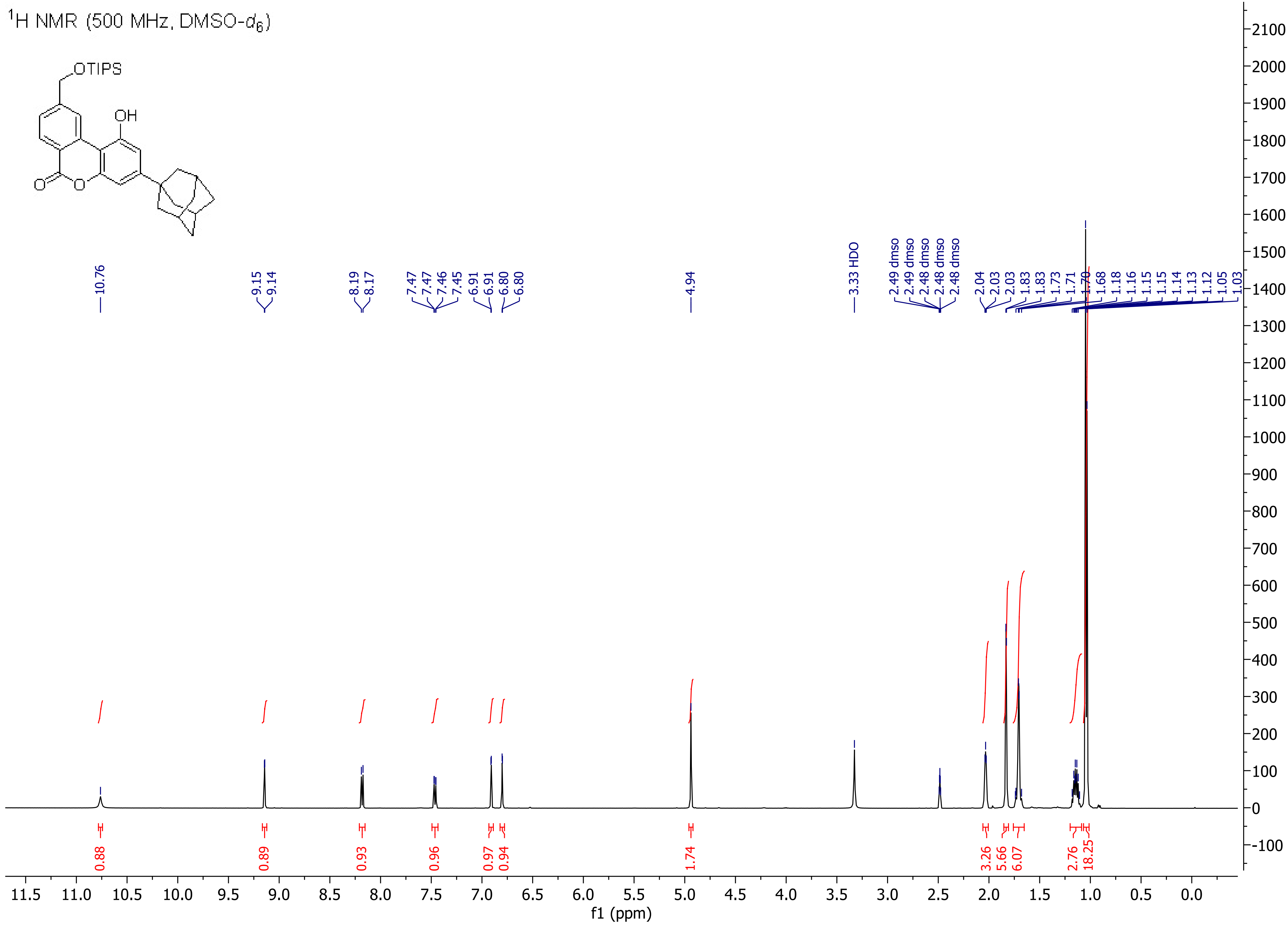

1H NMR (500 MHz, DMSO-d6)
OTIPS
OH
O
O
10.76
9.15
9.14
8.19
8.17
7.47
7.47
7.46
7.45
6.91
6.91
6.80
6.80
4.94
3.33 HDO
2.49 dmso
2.49 dmso
2.48 dmso
2.48 dmso
2.48 dmso
2.04
2.03
2.03
1.83
1.83
1.73
1.71
1.70
1.68
1.18
1.16
1.15
1.15
1.14
1.13
1.12
1.05
1.03
0.88
0.89
0.93
0.96
0.97
0.94
1.74
3.26
5.66
6.07
2.76
18.25
f1 (ppm)

^{13}C NMR (126 MHz, DMSO-d_6)

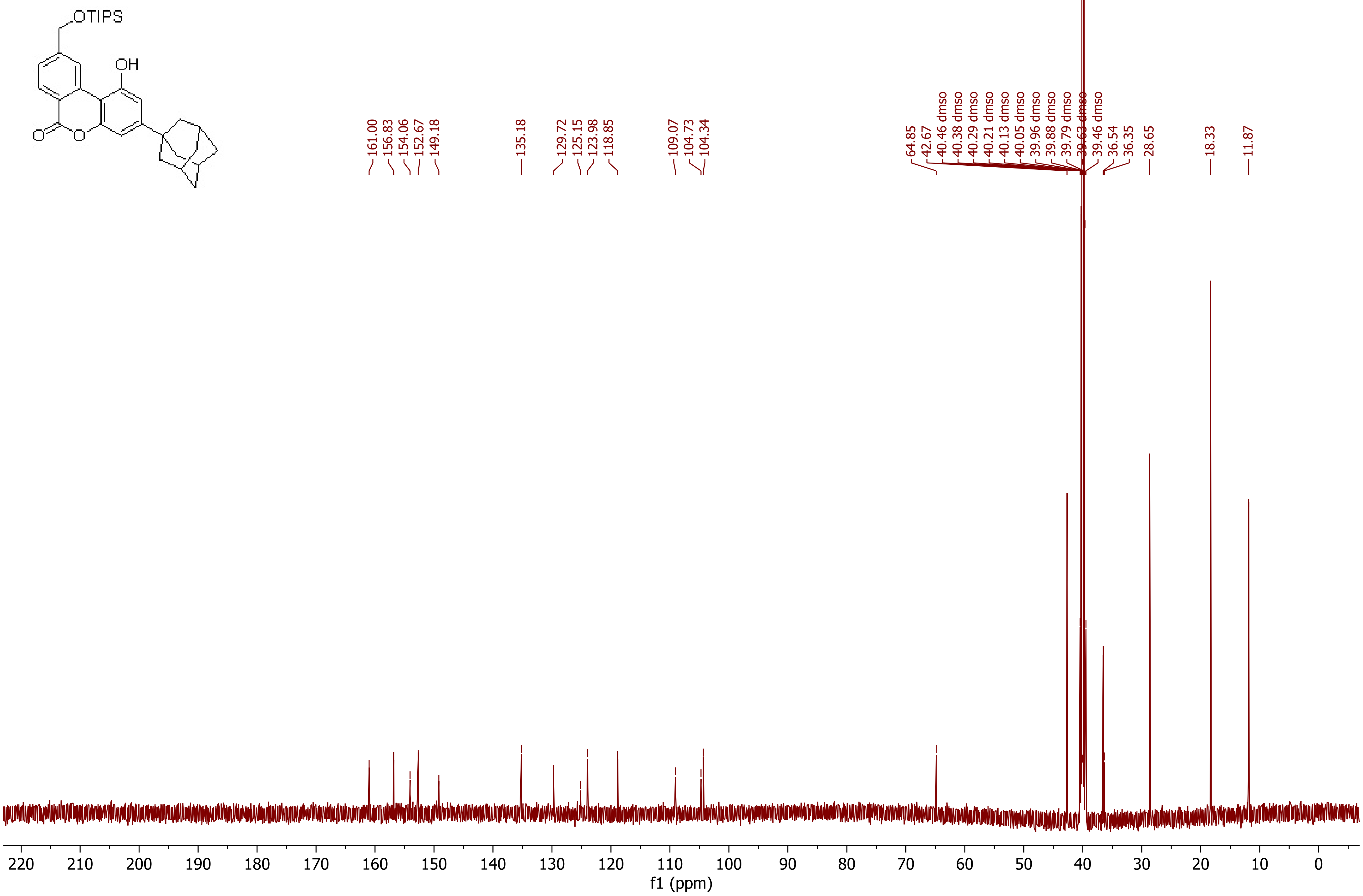

^{1}H NMR (500 MHz, DMSO-d_6)

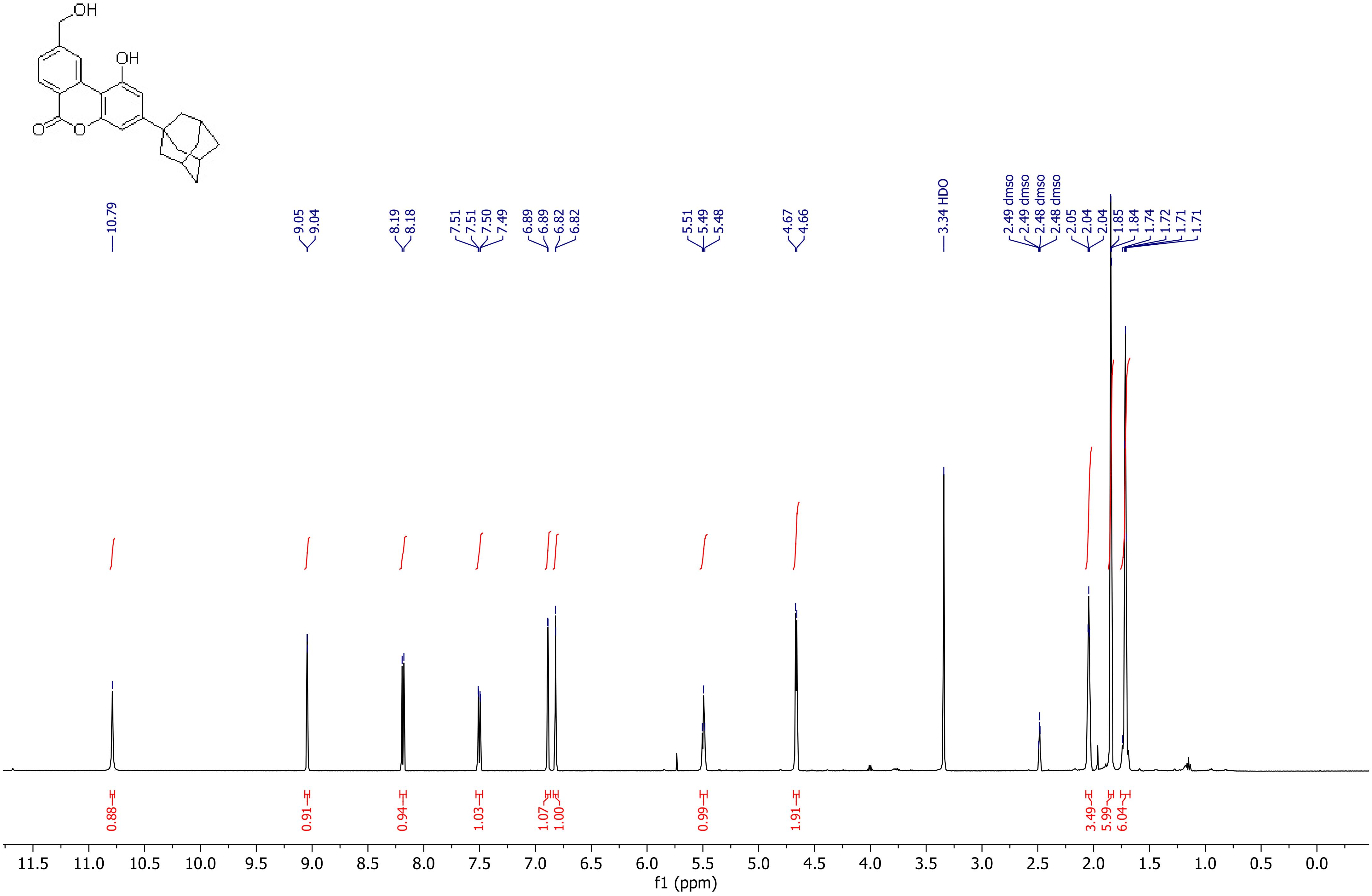

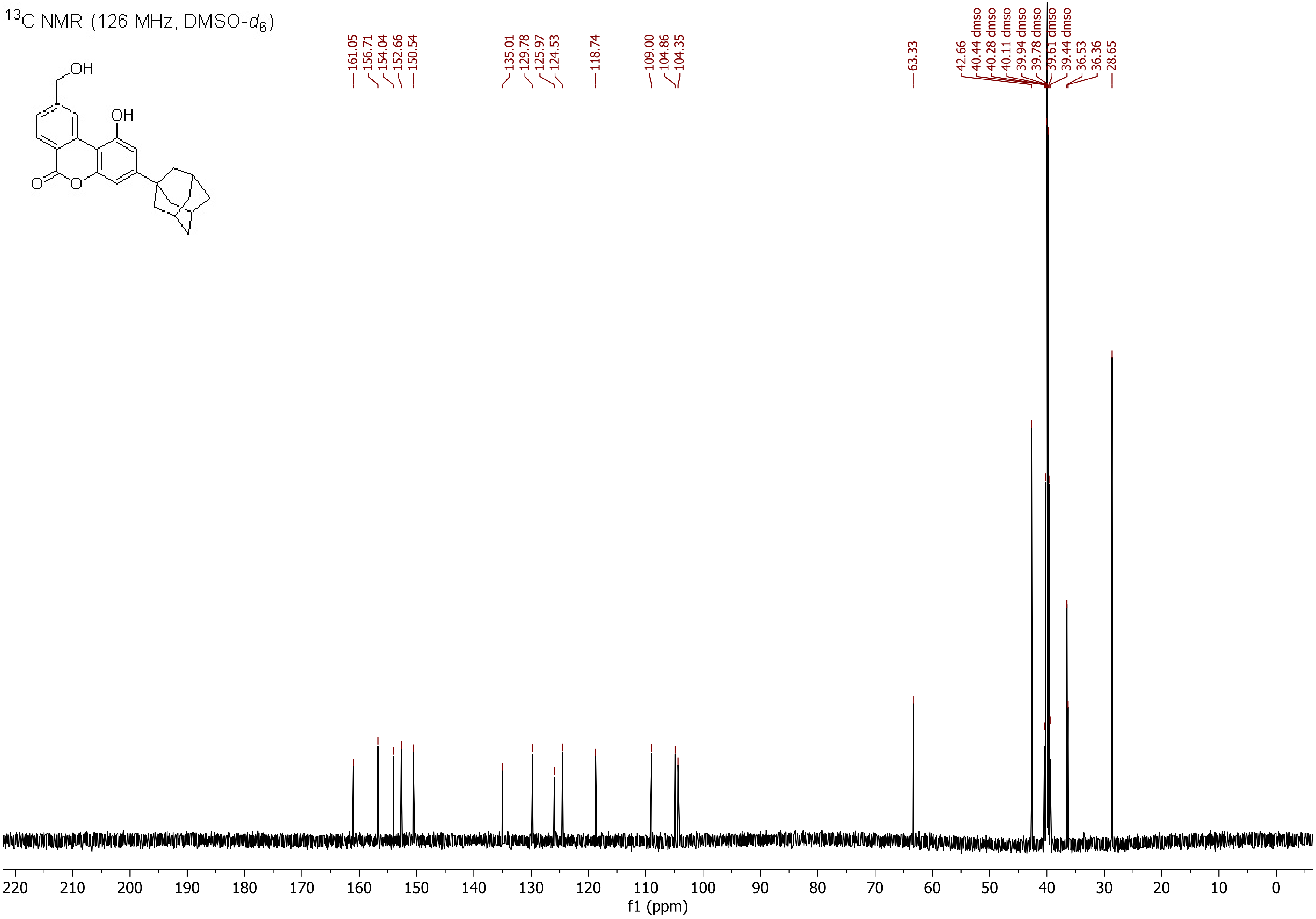

^{13}C NMR (126 MHz, DMSO-d_6)
OH
OH
O
O
161.05
156.71
154.04
152.66
150.54
135.01
129.78
125.97
124.53
118.74
109.00
104.86
104.35
63.33
42.66
40.44 dmso
40.28 dmso
40.11 dmso
39.94 dmso
39.78 dmso
39.61 dmso
39.44 dmso
36.53
36.36
28.65
220 210 200 190 180 170 160 150 140 130 120 110 100 90 80 70 60 50 40 30 20 10 0
f1 (ppm)

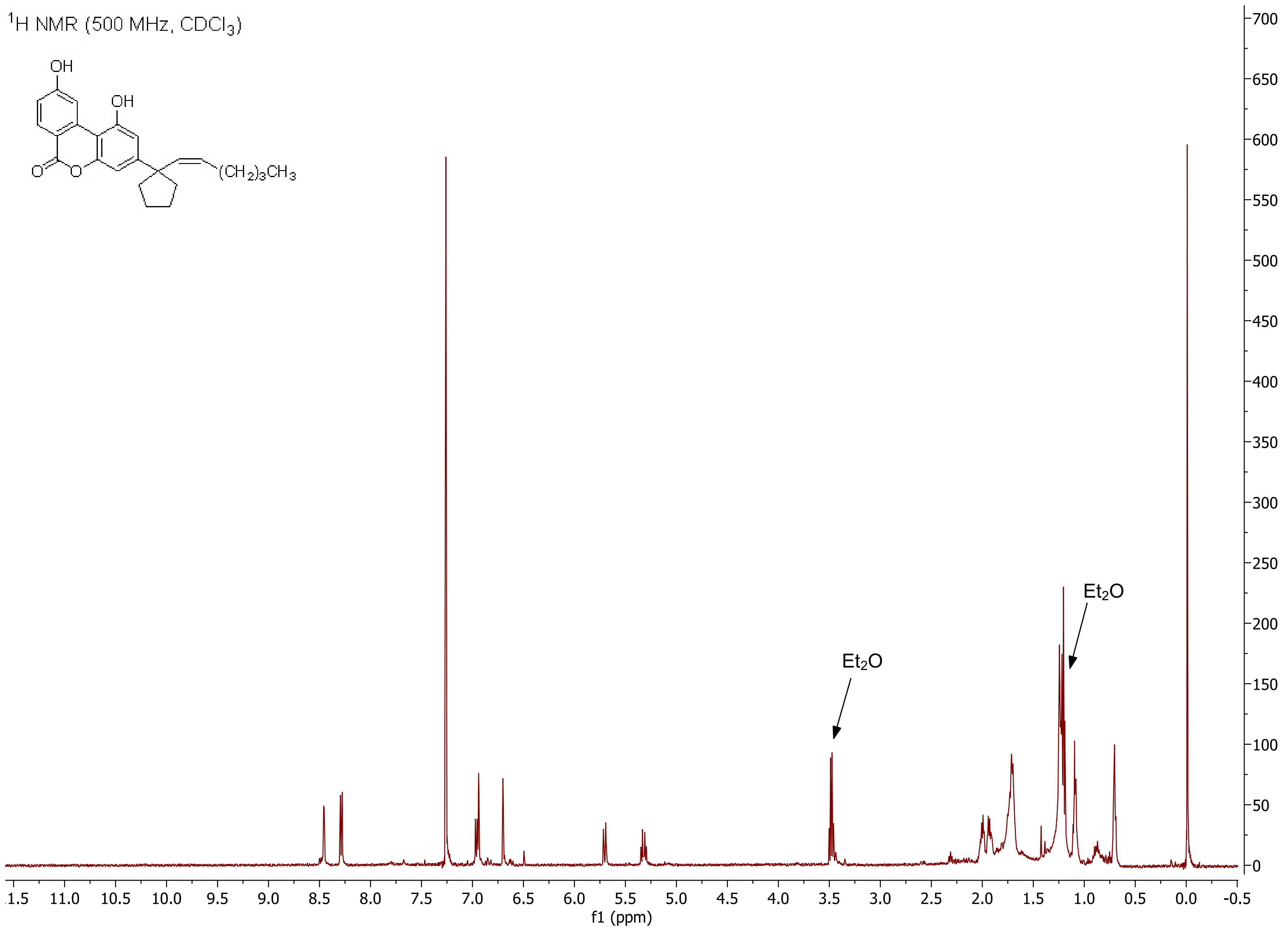

^1H NMR (500 MHz, $CDCl_3$)
OH
OH
O
O
$(CH_2)_3CH_3$
Et_2O
Et_2O
f1 (ppm)

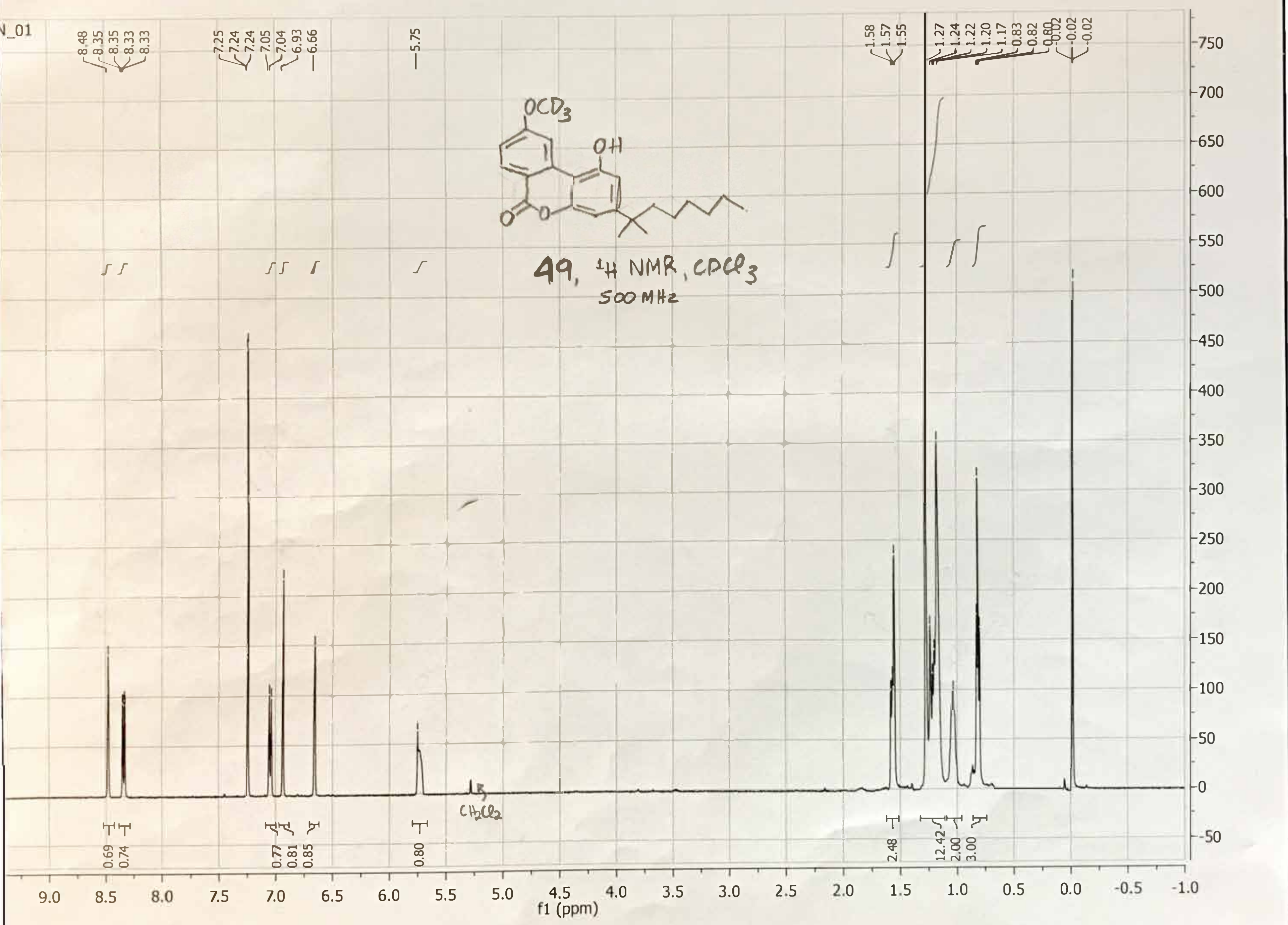
N_01
8.48
8.35
8.35
8.33
8.33
7.25
7.24
7.24
7.05
7.04
6.93
6.66
5.75
1.58
1.57
1.55
1.27
1.24
1.22
1.20
1.17
0.83
0.82
0.80
0.02
-0.02
-0.02
OCD3
OH
49, 1H NMR, CDCl3
500 MHz
CH2Cl2
0.69
0.74
0.77
0.81
0.85
0.80
2.48
12.42
2.00
3.00
f1 (ppm)

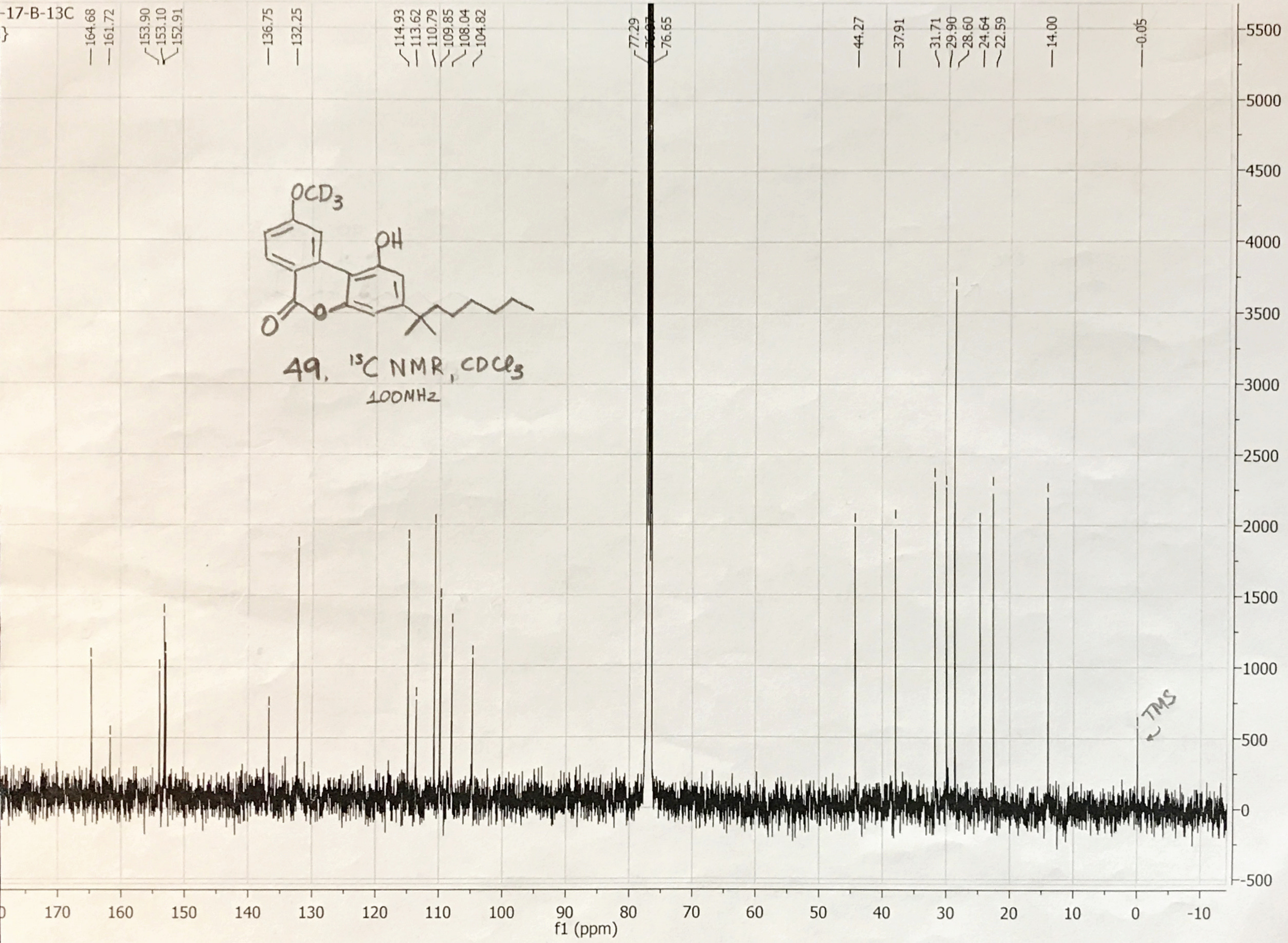
-17-B-13C
164.68
161.72
153.90
153.10
152.91
136.75
132.25
114.93
113.62
110.79
109.85
108.04
104.82
77.29
76.65
44.27
37.91
31.71
29.90
28.60
24.64
22.59
14.00
-0.05
OCD3
OH
O
49. 13C NMR, CDCl3
100MHz
TMS
170
160
150
140
130
120
110
100
90
80
70
60
50
40
30
20
10
0
-10
f1 (ppm)
5500
5000
4500
4000
3500
3000
2500
2000
1500
1000
500
0
-500

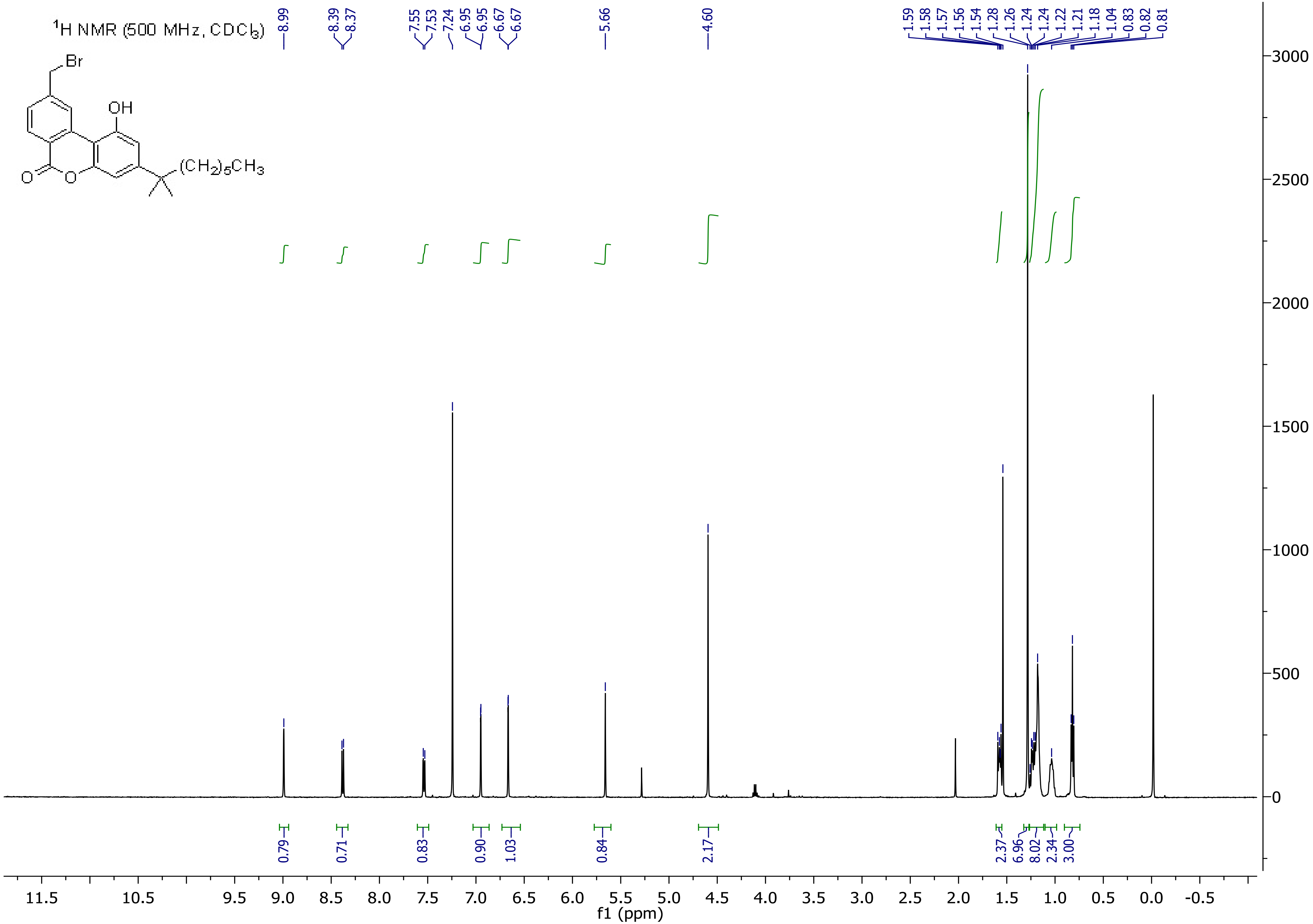

1H NMR (500 MHz, CDCl3)
Br
OH
O
O
(CH2)5CH3
8.99
8.39
8.37
7.55
7.53
7.24
6.95
6.95
6.67
6.67
5.66
4.60
1.59
1.58
1.57
1.56
1.54
1.28
1.26
1.24
1.24
1.22
1.21
1.18
1.04
0.83
0.82
0.81
0.79
0.71
0.83
0.90
1.03
0.84
2.17
2.37
6.96
8.02
2.34
3.00
f1 (ppm)

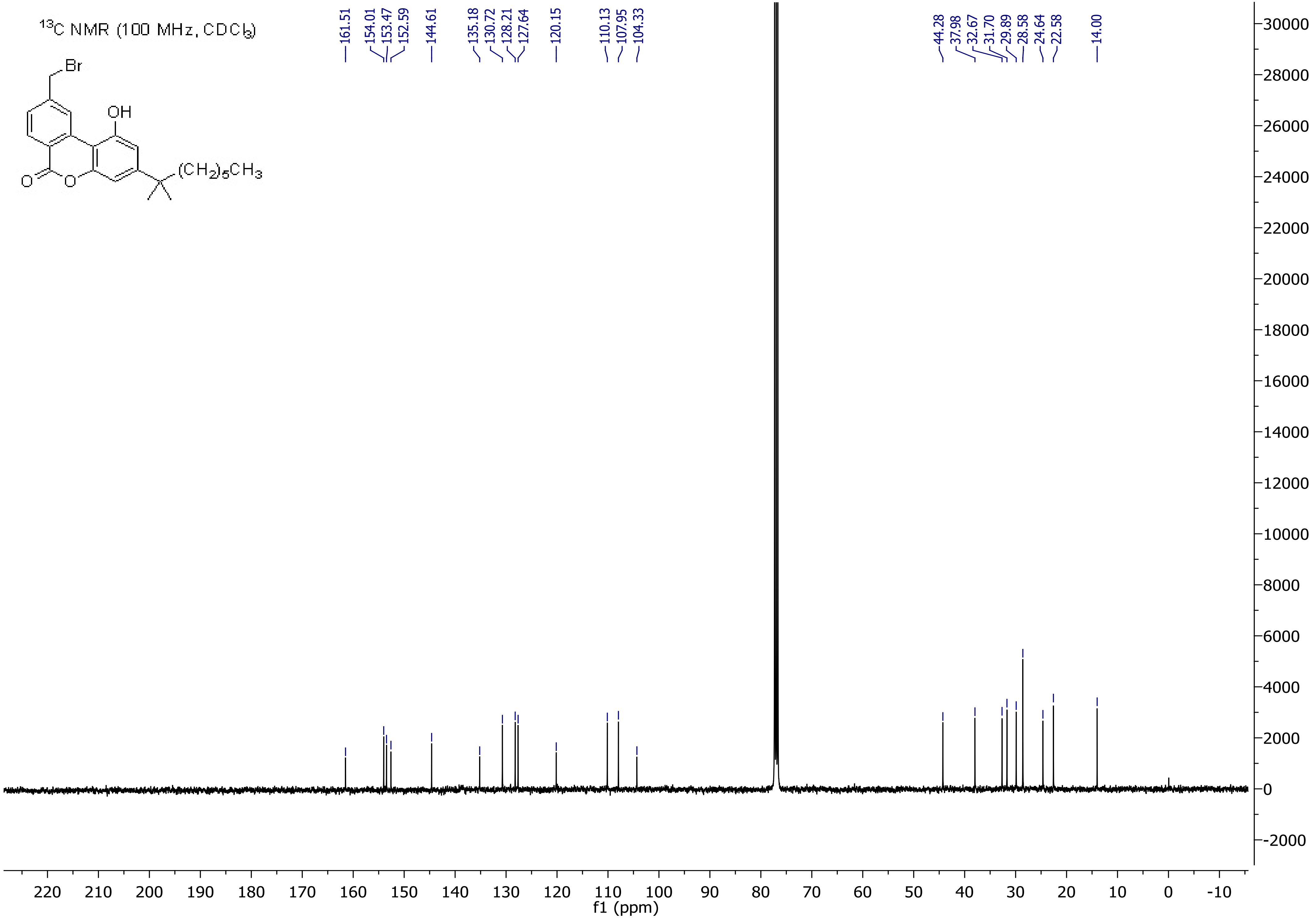

^{13}C NMR (100 MHz, CDCl$_3$)
Br
OH
(CH$_2$)$_5$CH$_3$
O
O
161.51
154.01
153.47
152.59
144.61
135.18
130.72
128.21
127.64
120.15
110.13
107.95
104.33
44.28
37.98
32.67
31.70
29.89
28.58
24.64
22.58
14.00
f1 (ppm)

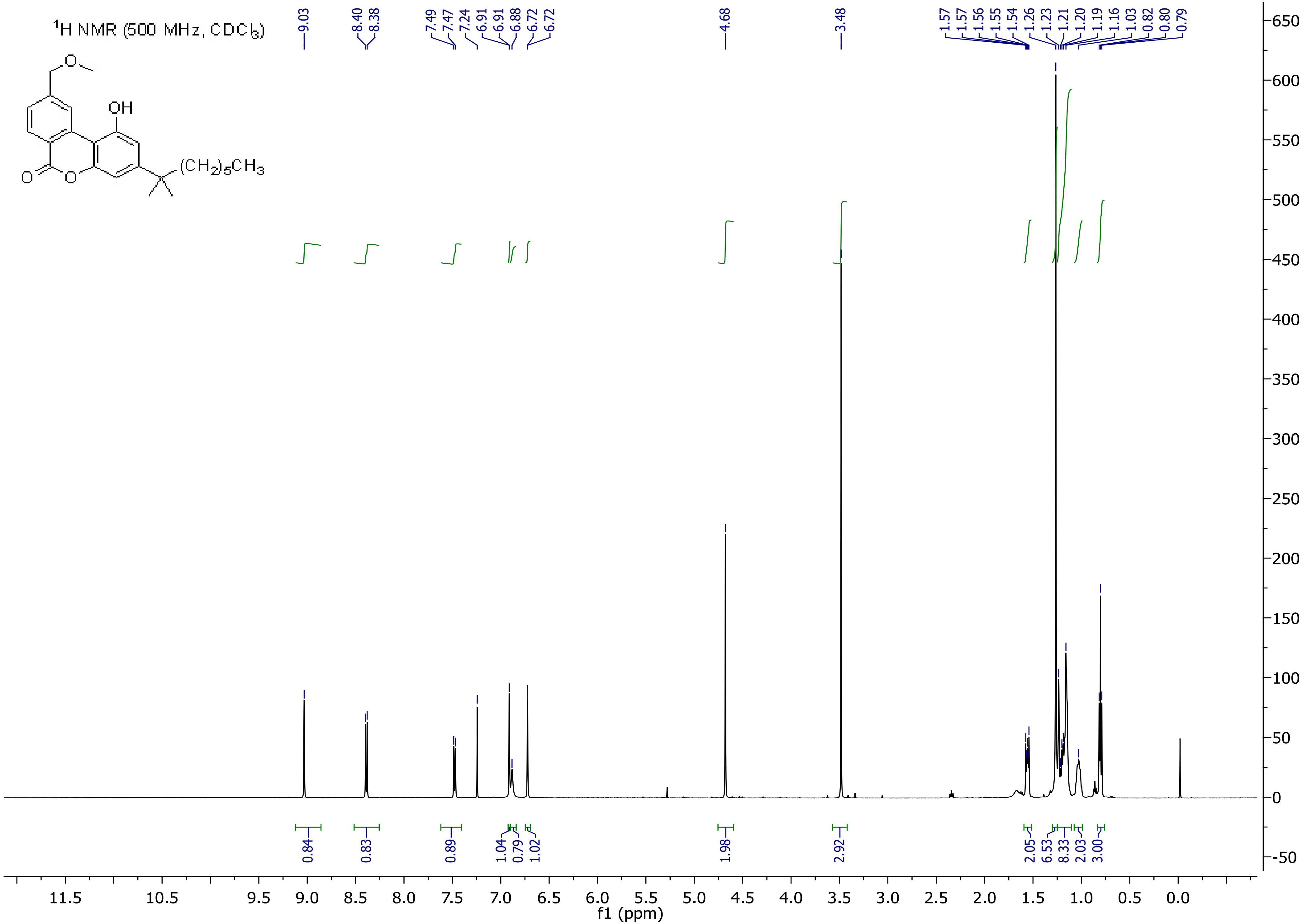

1H NMR (500 MHz, CDCl3)
OH
(CH2)5CH3
9.03
8.40
8.38
7.49
7.47
7.24
6.91
6.91
6.88
6.72
6.72
4.68
3.48
1.57
1.57
1.56
1.55
1.54
1.26
1.23
1.21
1.20
1.19
1.16
1.03
0.82
0.80
0.79
0.84
0.83
0.89
1.04
0.79
1.02
1.98
2.92
2.05
6.53
8.33
2.03
3.00
f1 (ppm)

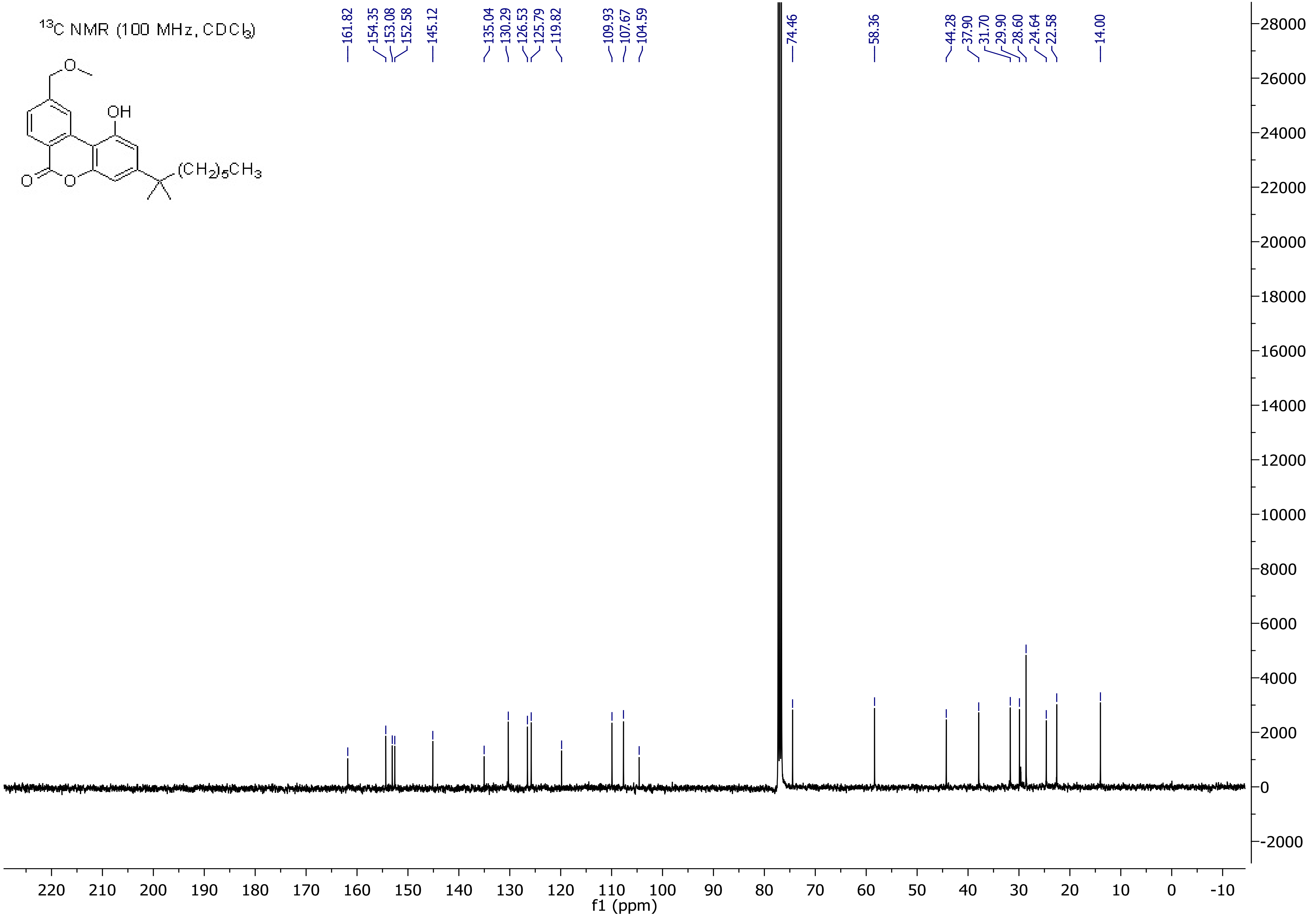

13C NMR (100 MHz, CDCl3)
OH
O
(CH2)5CH3
161.82
154.35
153.08
152.58
145.12
135.04
130.29
126.53
125.79
119.82
109.93
107.67
104.59
74.46
58.36
44.28
37.90
31.70
29.90
28.60
24.64
22.58
14.00
28000
26000
24000
22000
20000
18000
16000
14000
12000
10000
8000
6000
4000
2000
0
-2000
220 210 200 190 180 170 160 150 140 130 120 110 100 90 80 70 60 50 40 30 20 10 0 -10
f1 (ppm)

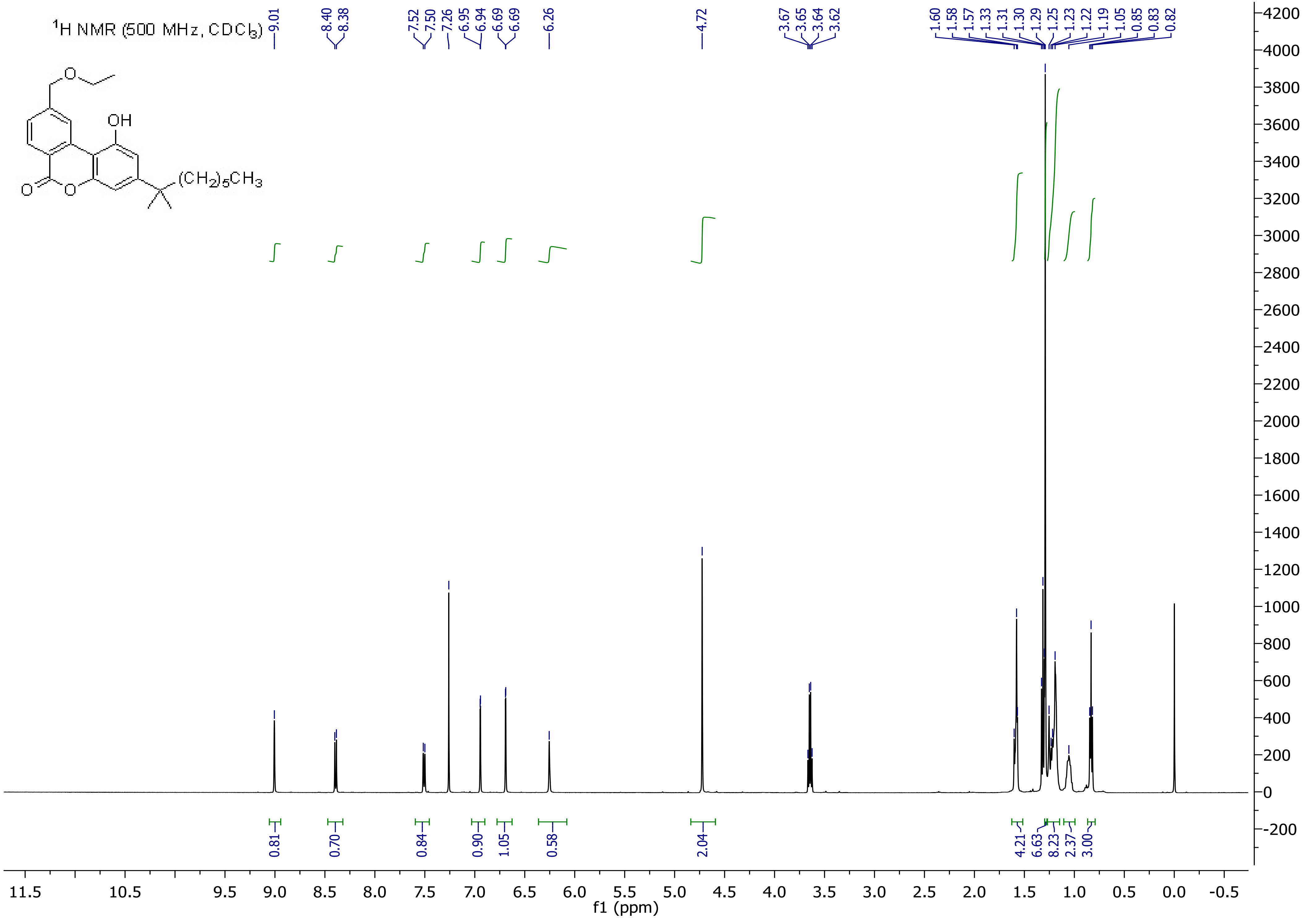

1H NMR (500 MHz, CDCl3)
OH
O
O
O
(CH2)5CH3
9.01
8.40
8.38
7.52
7.50
7.26
6.95
6.94
6.69
6.69
6.26
4.72
3.67
3.65
3.64
3.62
1.60
1.58
1.57
1.33
1.31
1.30
1.29
1.25
1.23
1.22
1.19
1.05
0.85
0.83
0.82
0.81
0.70
0.84
0.90
1.05
0.58
2.04
4.21
6.63
8.23
2.37
3.00
f1 (ppm)

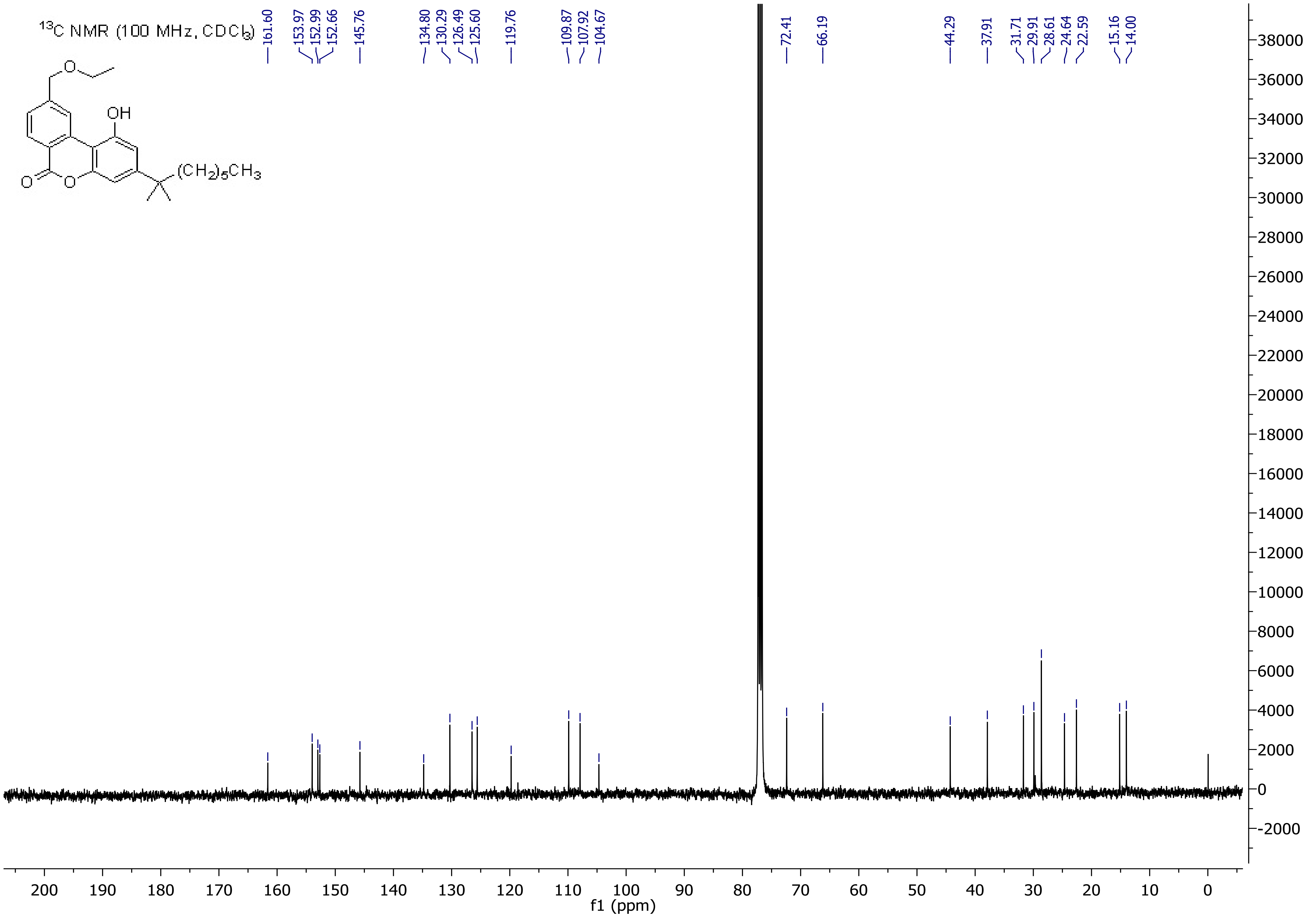

13C NMR (100 MHz, CDCl3)
OH
O
(CH2)5CH3
161.60
153.97
152.99
152.66
145.76
134.80
130.29
126.49
125.60
119.76
109.87
107.92
104.67
72.41
66.19
44.29
37.91
31.71
29.91
28.61
24.64
22.59
15.16
14.00
f1 (ppm)

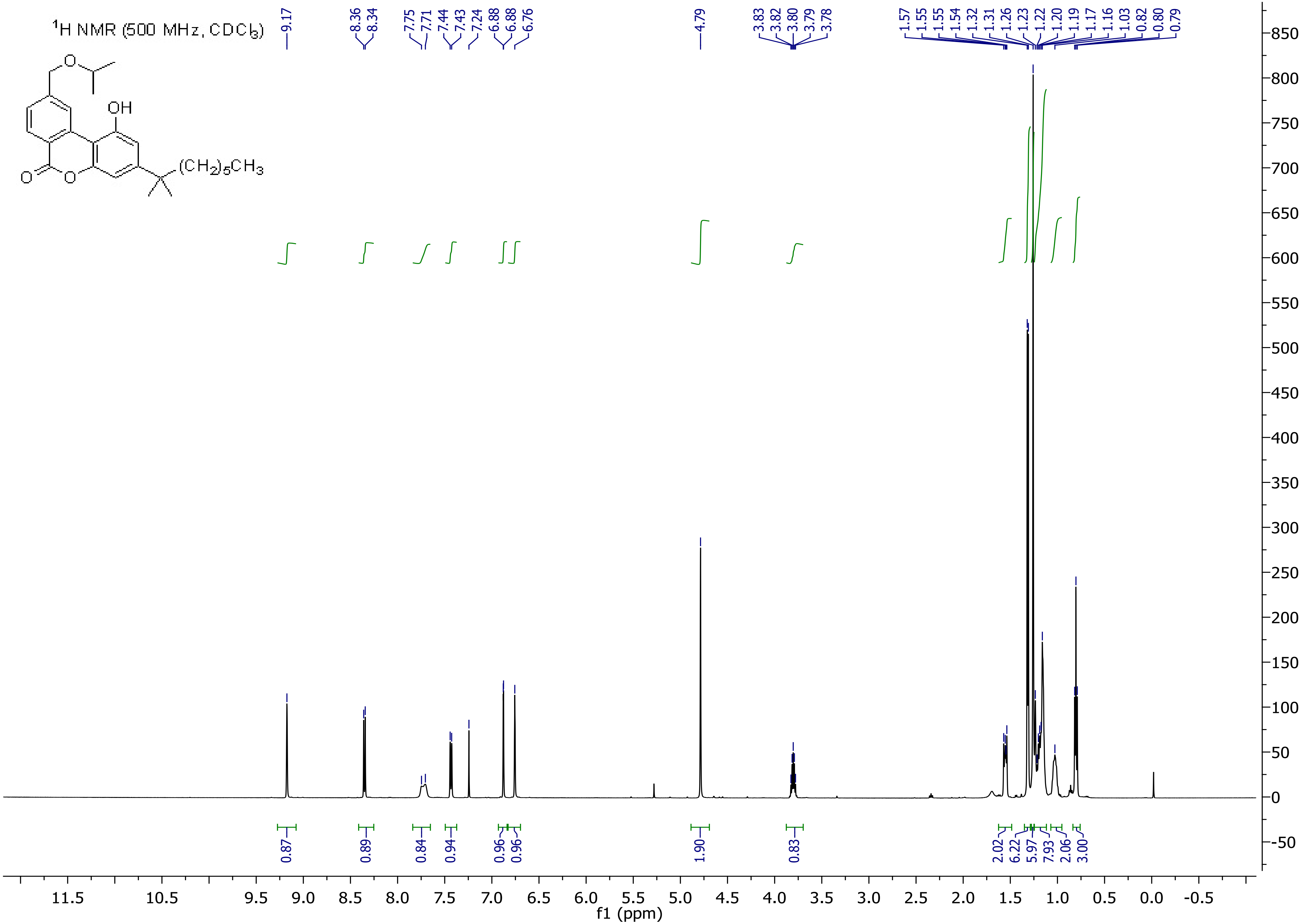

¹H NMR (500 MHz, CDCl3)
OH
(CH2)5CH3
9.17
8.36 8.34
7.75 7.71 7.44 7.43 7.24 6.88 6.88 6.76
4.79
3.83 3.82 3.80 3.79 3.78
1.57 1.55 1.55 1.54 1.32 1.31 1.26 1.23 1.22 1.20 1.19 1.17 1.16 1.03 0.82 0.80 0.79
0.87 0.89 0.84 0.94 0.96 0.96 1.90 0.83 2.02 6.22 5.97 7.93 2.06 3.00
f1 (ppm)

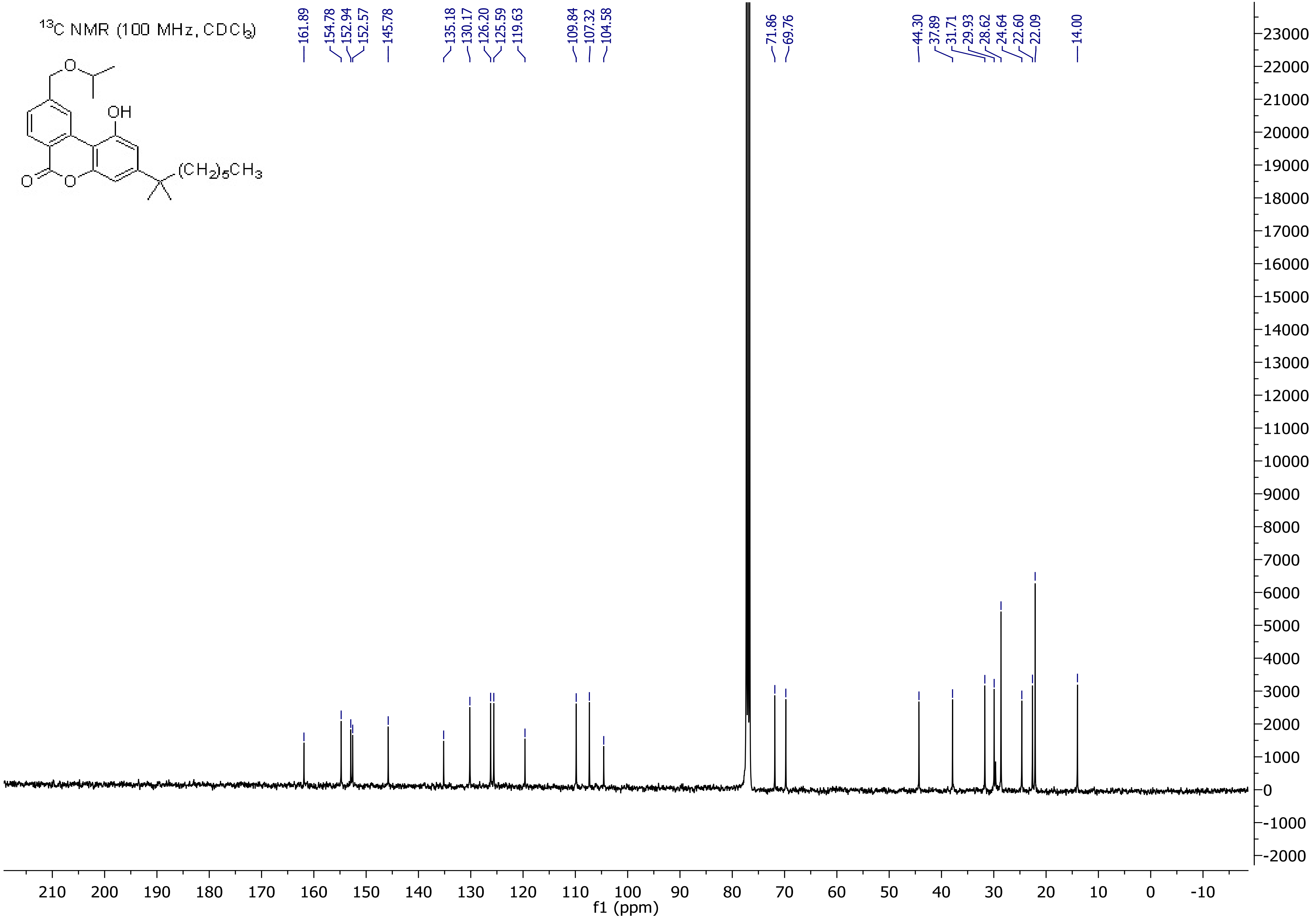

13C NMR (100 MHz, CDCl3)
OH
(CH2)5CH3
161.89
154.78
152.94
152.57
145.78
135.18
130.17
126.20
125.59
119.63
109.84
107.32
104.58
71.86
69.76
44.30
37.89
31.71
29.93
28.62
24.64
22.60
22.09
14.00
f1 (ppm)

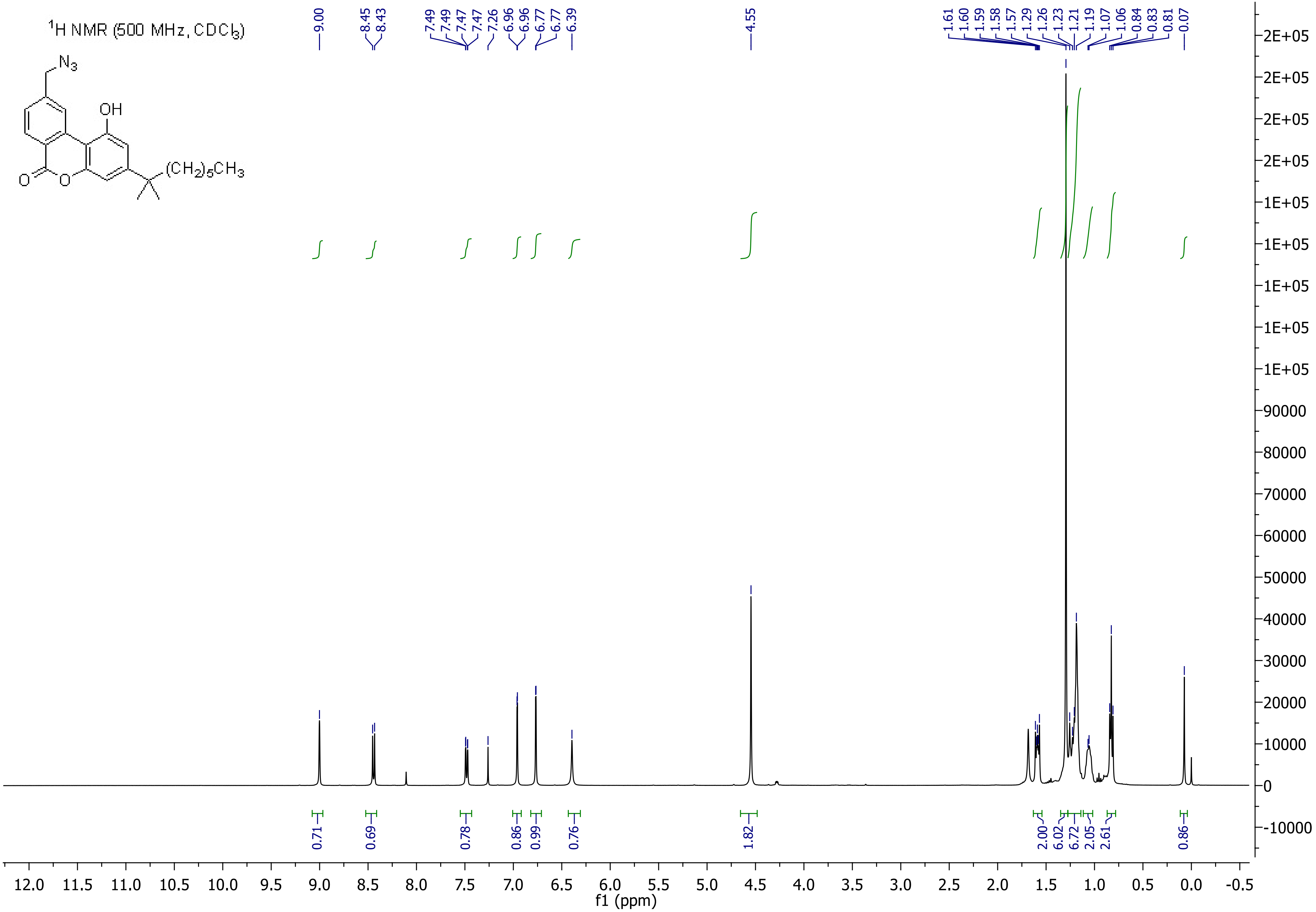

^{1}H NMR (500 MHz, $CDCl_3$)
N3
OH
O
O
(CH2)5CH3
9.00
8.45
8.43
7.49
7.49
7.47
7.47
7.26
6.96
6.96
6.77
6.77
6.39
4.55
1.61
1.60
1.59
1.58
1.57
1.29
1.26
1.23
1.21
1.19
1.07
1.06
0.84
0.83
0.81
0.07
0.71
0.69
0.78
0.86
0.99
0.76
1.82
2.00
6.02
6.72
2.05
2.61
0.86
f1 (ppm)

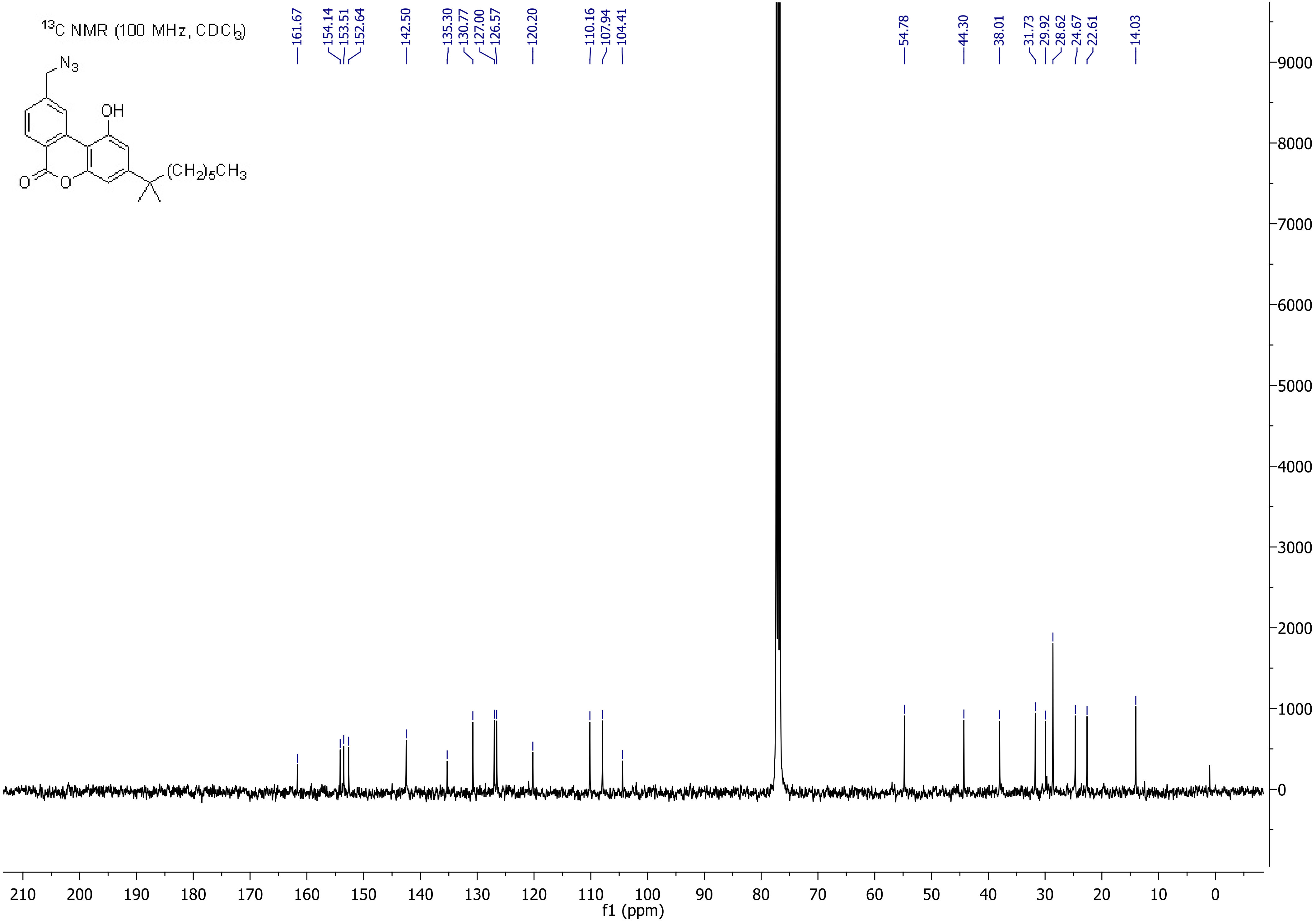

13C NMR (100 MHz, CDCl3)
N3
OH
(CH2)5CH3
O
O
161.67
154.14
153.51
152.64
142.50
135.30
130.77
127.00
126.57
120.20
110.16
107.94
104.41
54.78
44.30
38.01
31.73
29.92
28.62
24.67
22.61
14.03
9000
8000
7000
6000
5000
4000
3000
2000
1000
0
210 200 190 180 170 160 150 140 130 120 110 100 90 80 70 60 50 40 30 20 10 0
f1 (ppm)

^{1}H NMR (500 MHz, $CDCl_3$)

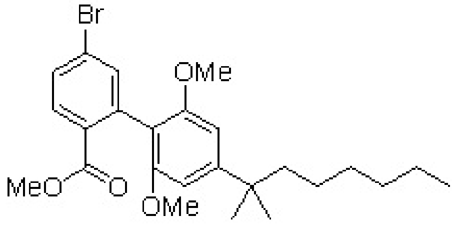

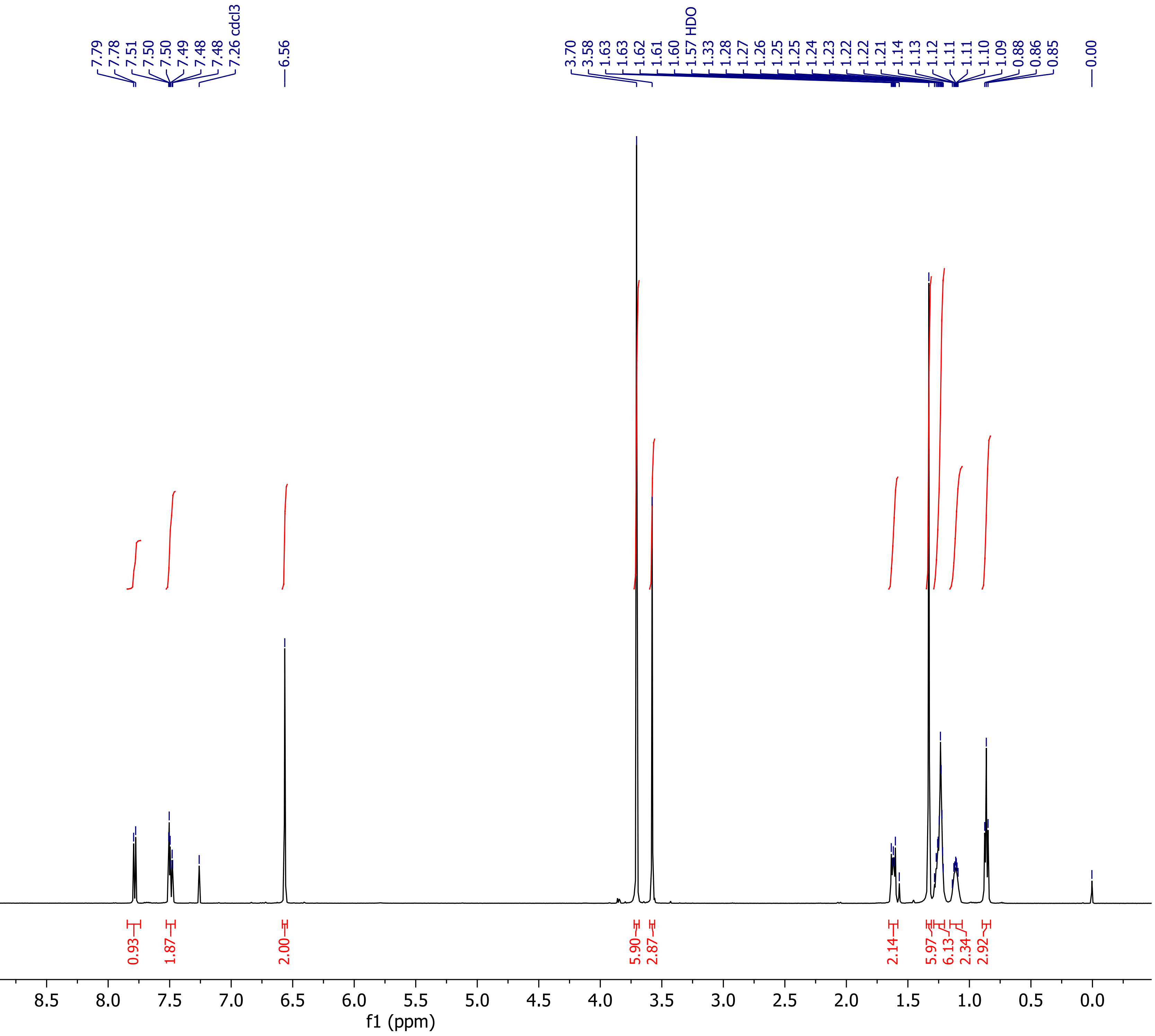

^{13}C NMR (126 MHz, $CDCl_3$)

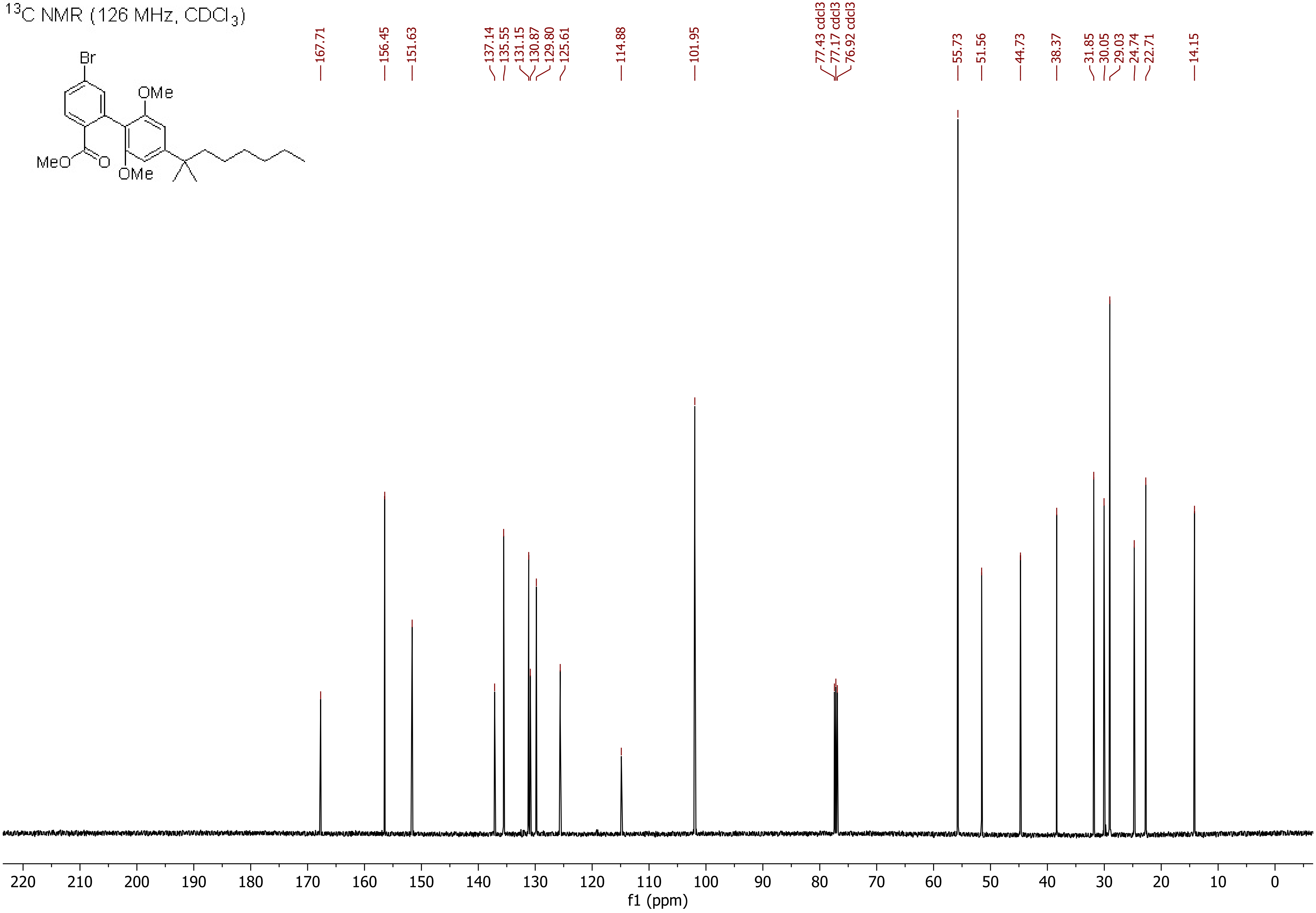

^1H NMR (500 MHz, DMSO-d_6)

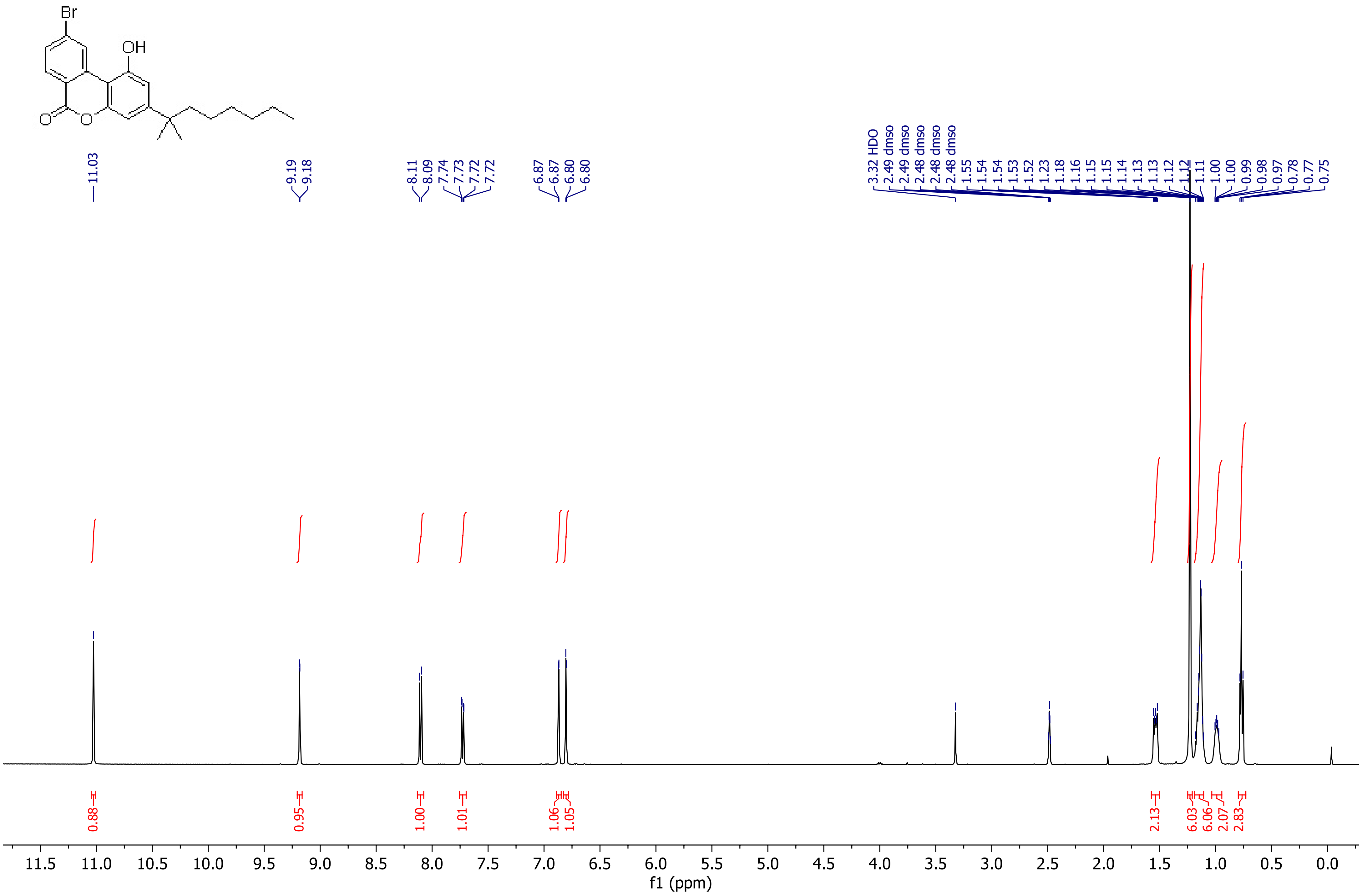

^{13}C NMR (126 MHz, DMSO-d_6)

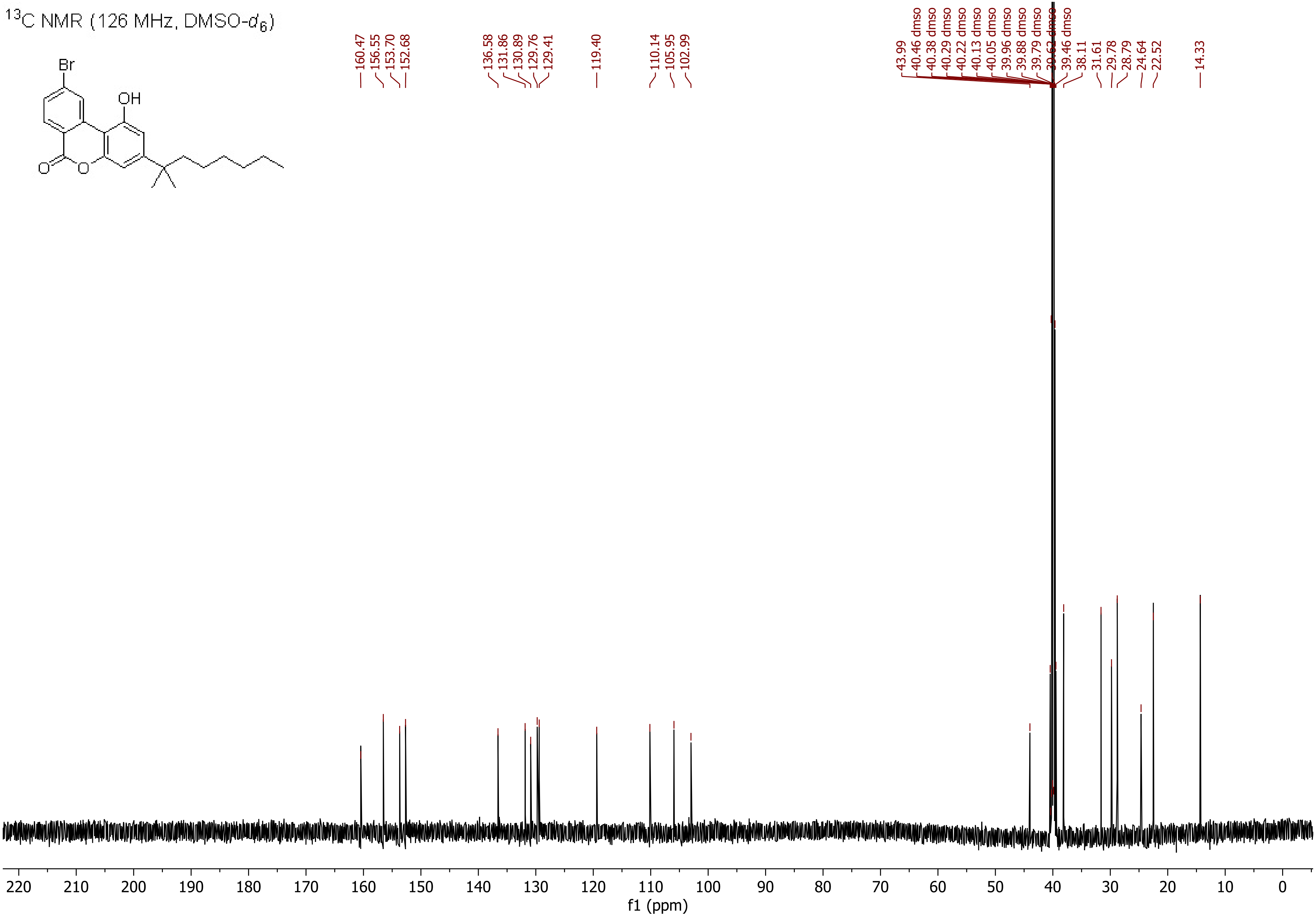

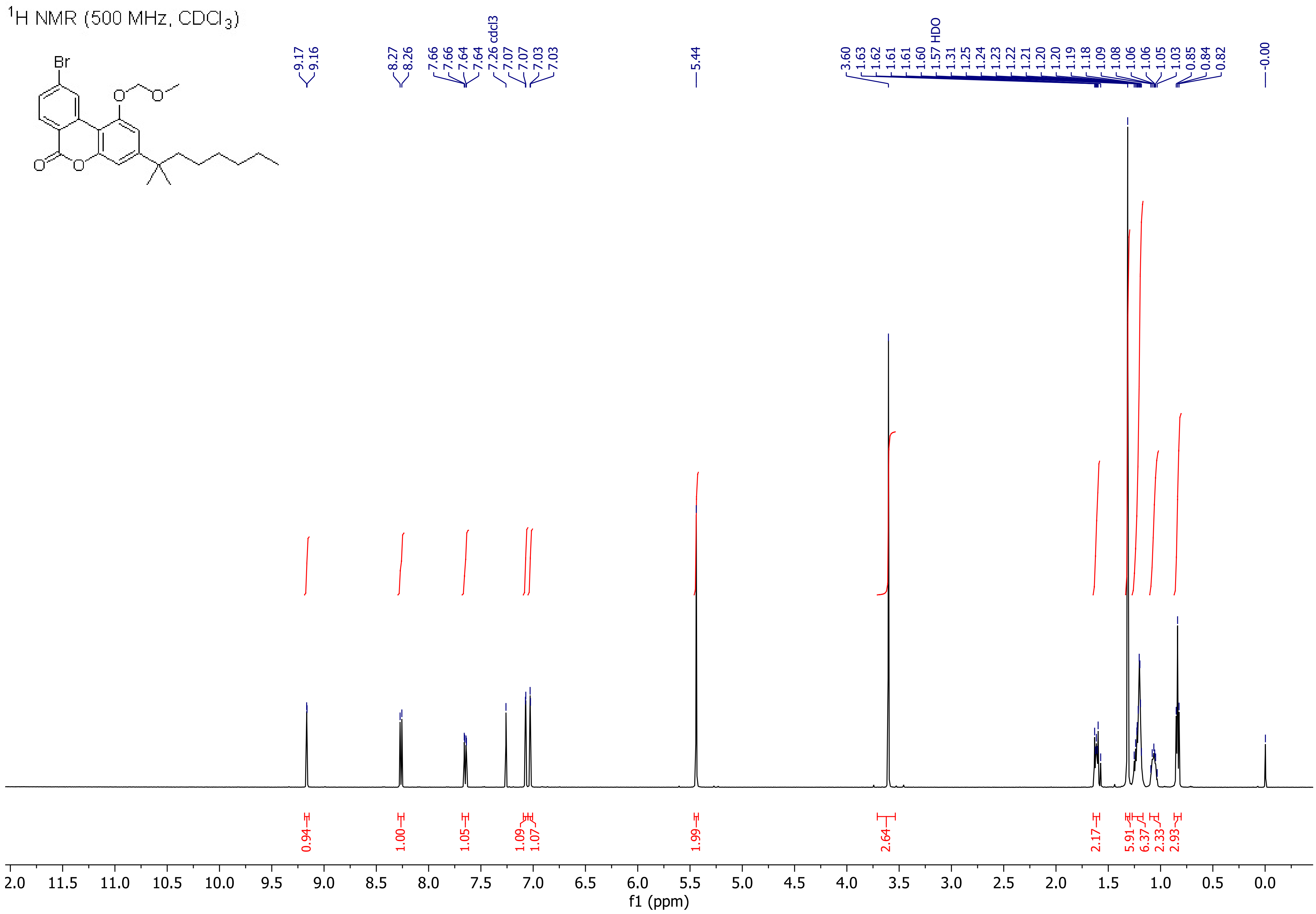

1H NMR (500 MHz, CDCl3)
Br
O
O
O
O
9.17
9.16
8.27
8.26
7.66
7.66
7.64
7.64
7.26 cdcl3
7.07
7.07
7.03
7.03
5.44
3.60
1.63
1.62
1.61
1.61
1.60
1.57 HDO
1.31
1.25
1.24
1.23
1.22
1.21
1.20
1.20
1.19
1.18
1.09
1.08
1.06
1.06
1.05
1.03
0.85
0.84
0.82
-0.00
0.94
1.00
1.05
1.09
1.07
1.99
2.64
2.17
5.91
6.37
2.33
2.93
f1 (ppm)

^{13}C NMR (126 MHz, $CDCl_3$)

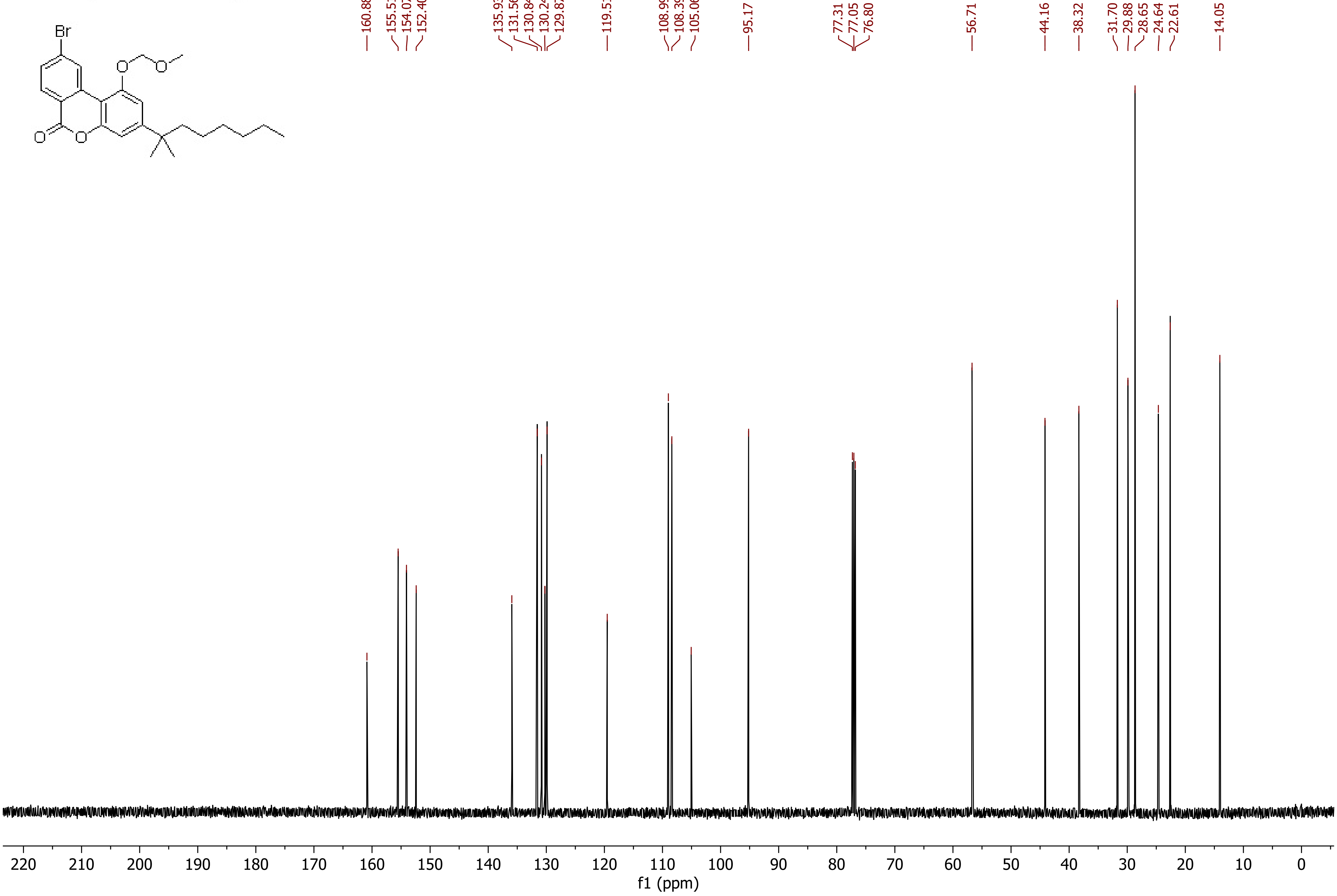

^1H NMR (500 MHz, $CDCl_3$)

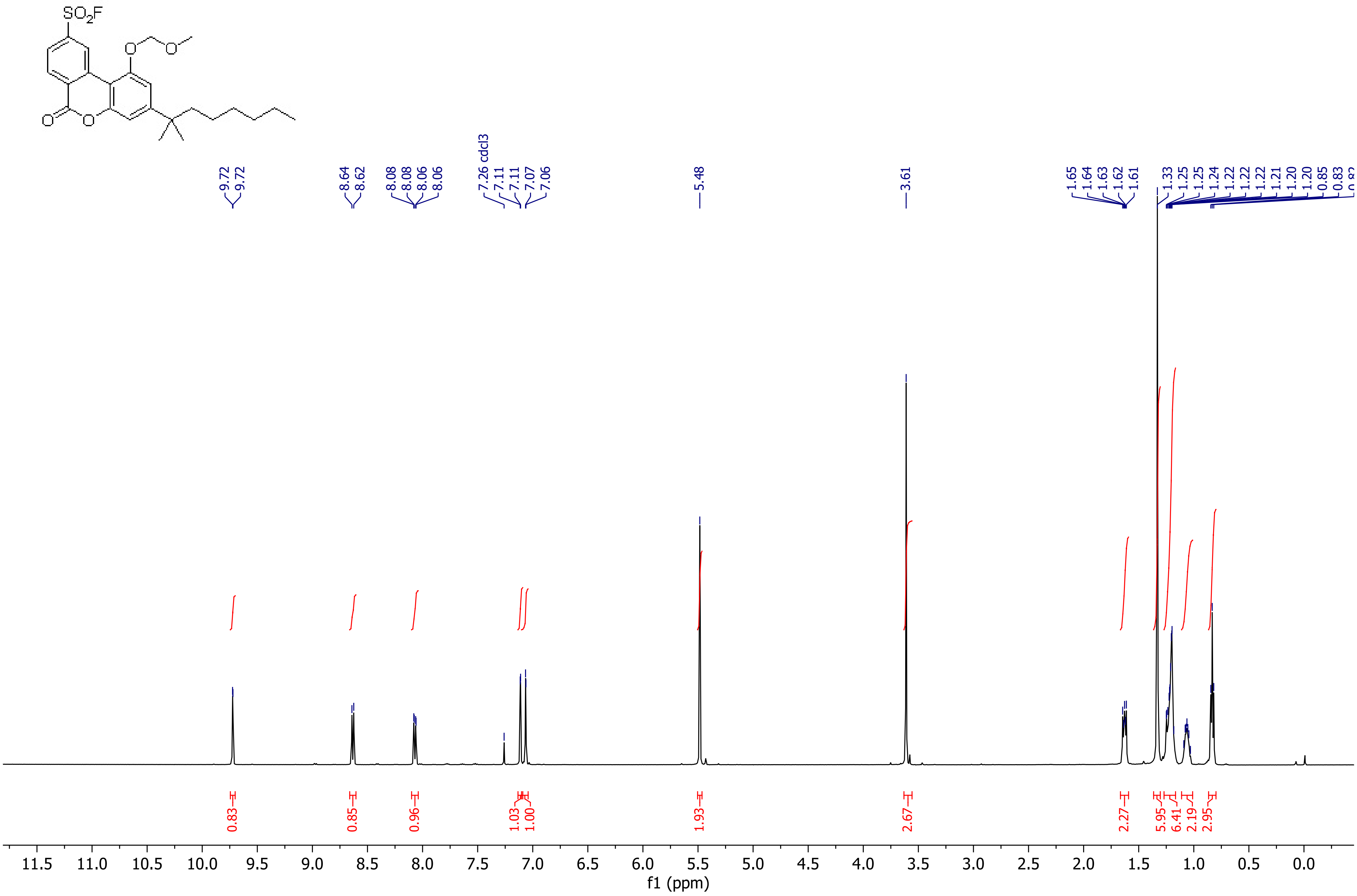

^{13}C NMR (126 MHz, $CDCl_3$)

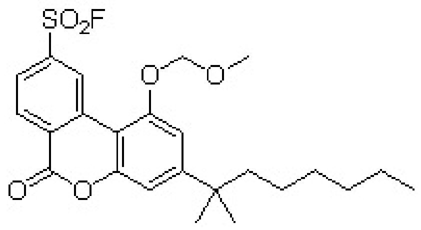

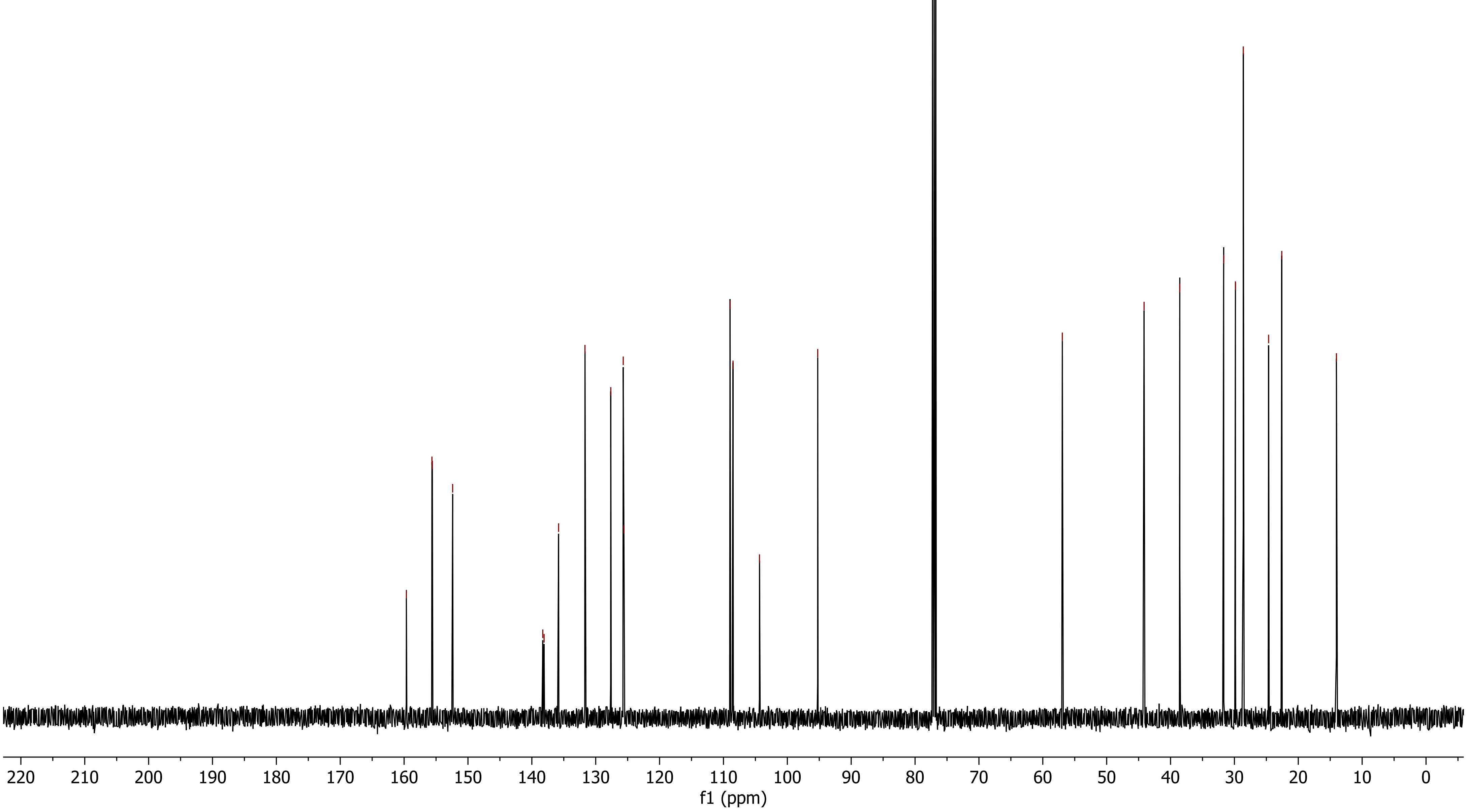

^{19}F NMR (470 MHz, $CDCl_3$, C_6H_5F reference standard)

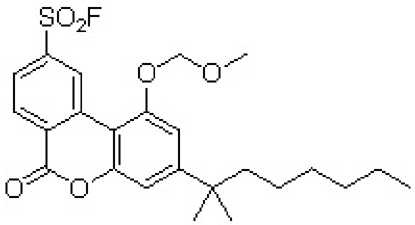

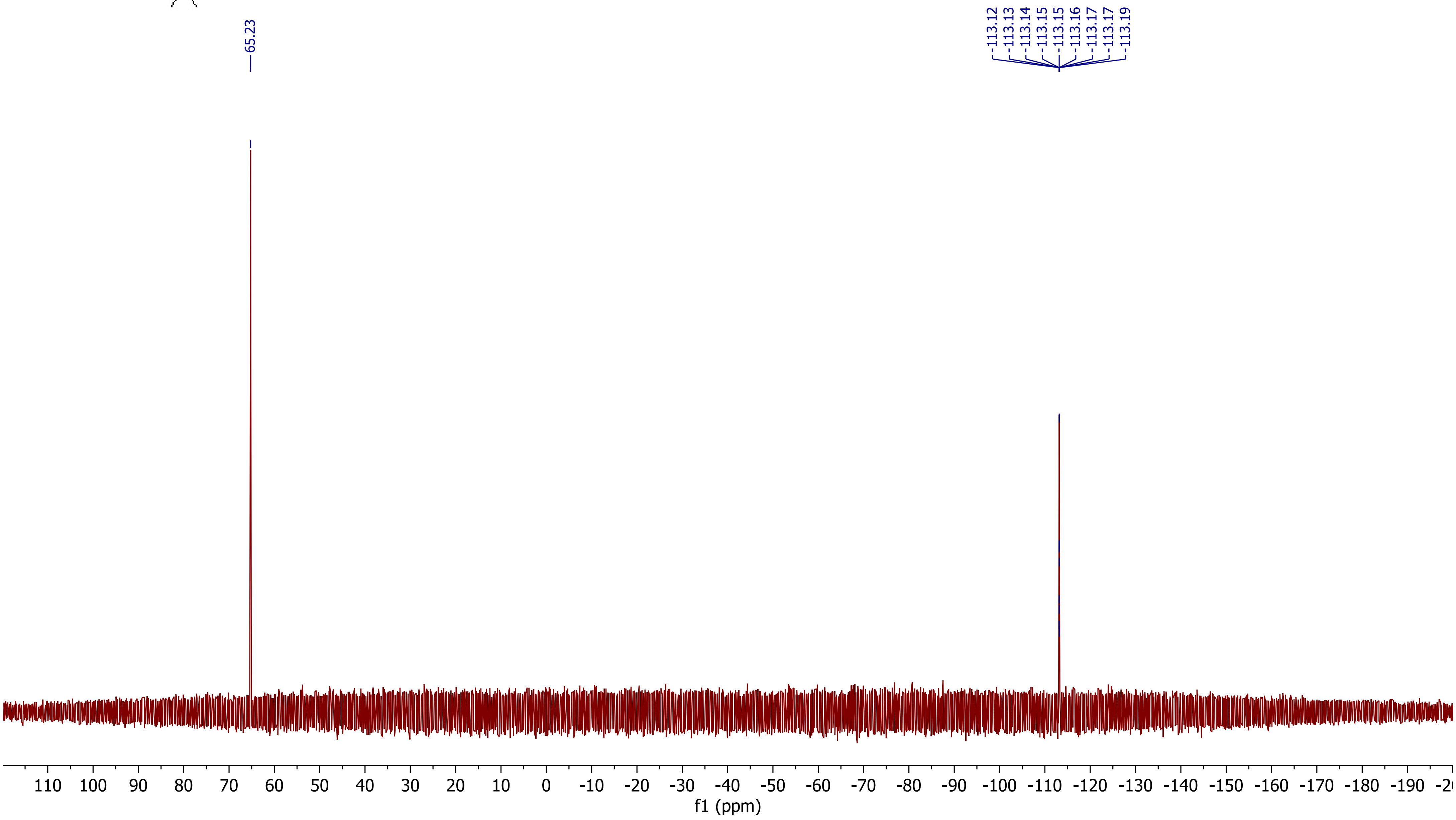

^1H NMR (500 MHz, CD_3CN)

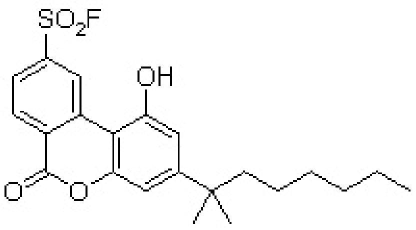

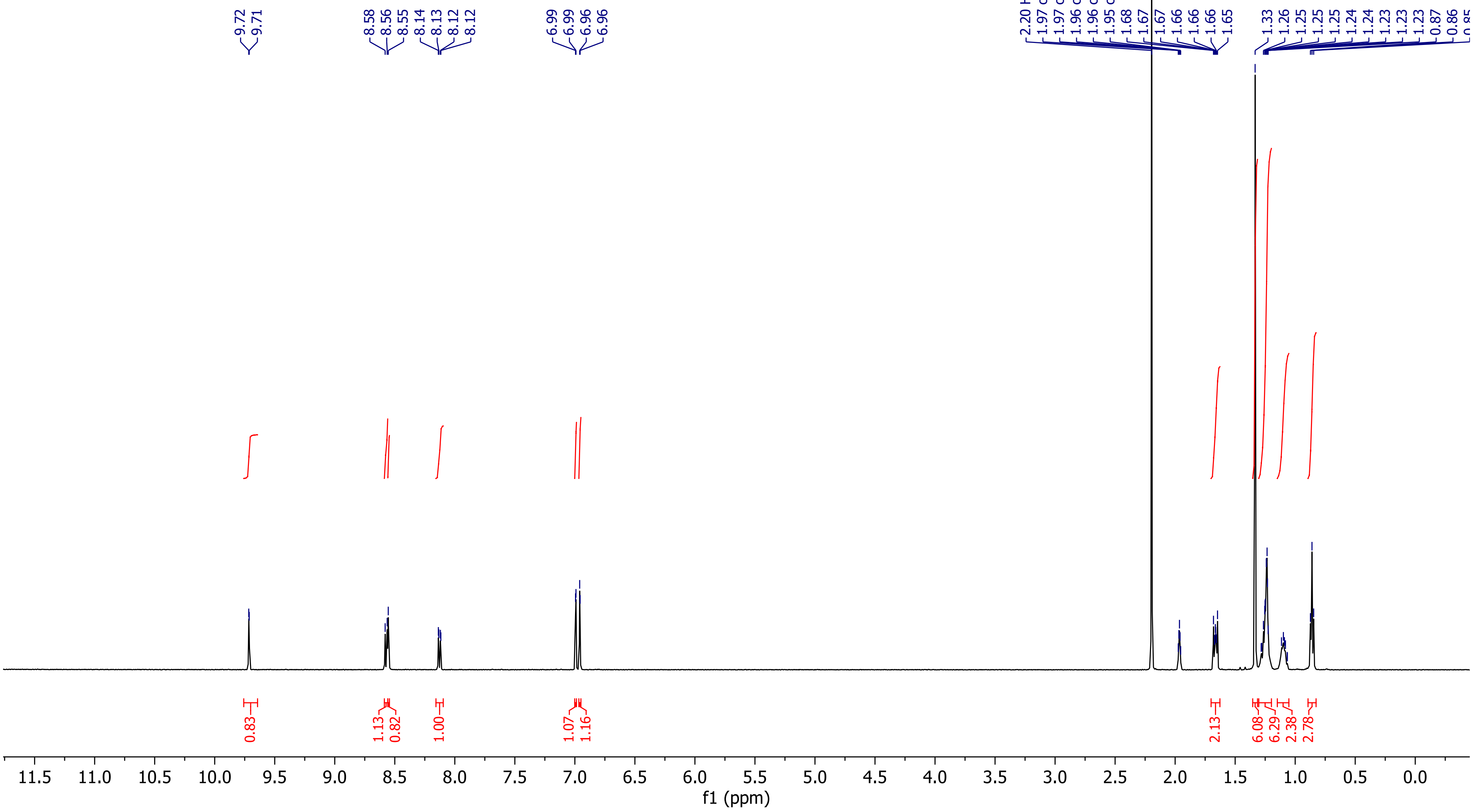

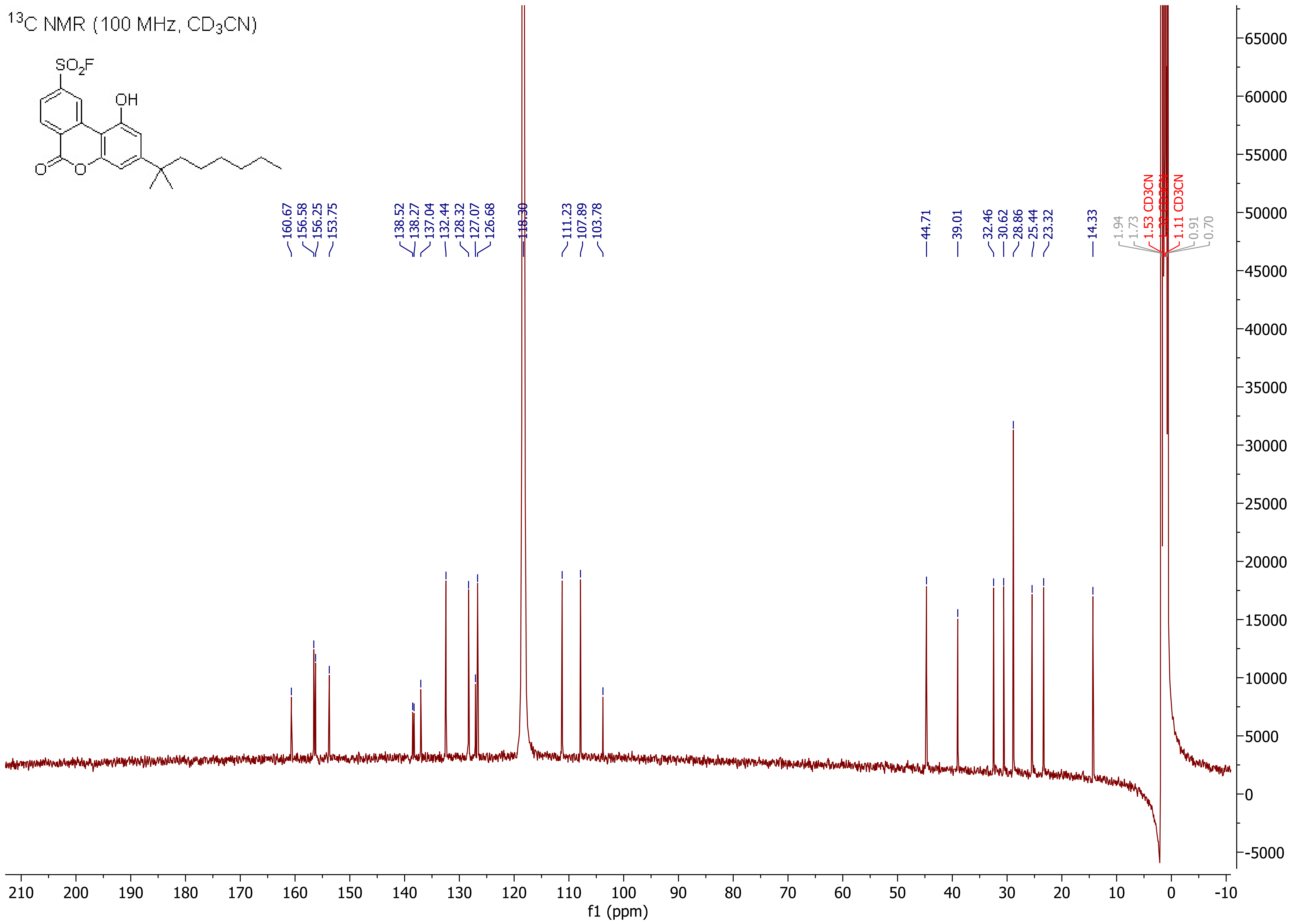

^{13}C NMR (100 MHz, CD_3CN)
SO_2F
OH
O
O
160.67
156.58
156.25
153.75
138.52
138.27
137.04
132.44
128.32
127.07
126.68
118.30
111.23
107.89
103.78
44.71
39.01
32.46
30.62
28.86
25.44
23.32
14.33
1.94
1.73
1.53 CD3CN
1.32 CD3CN
1.11 CD3CN
0.91
0.70
f1 (ppm)

^{19}F NMR (376 MHz, CD_3CN)

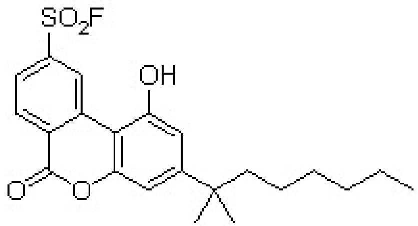

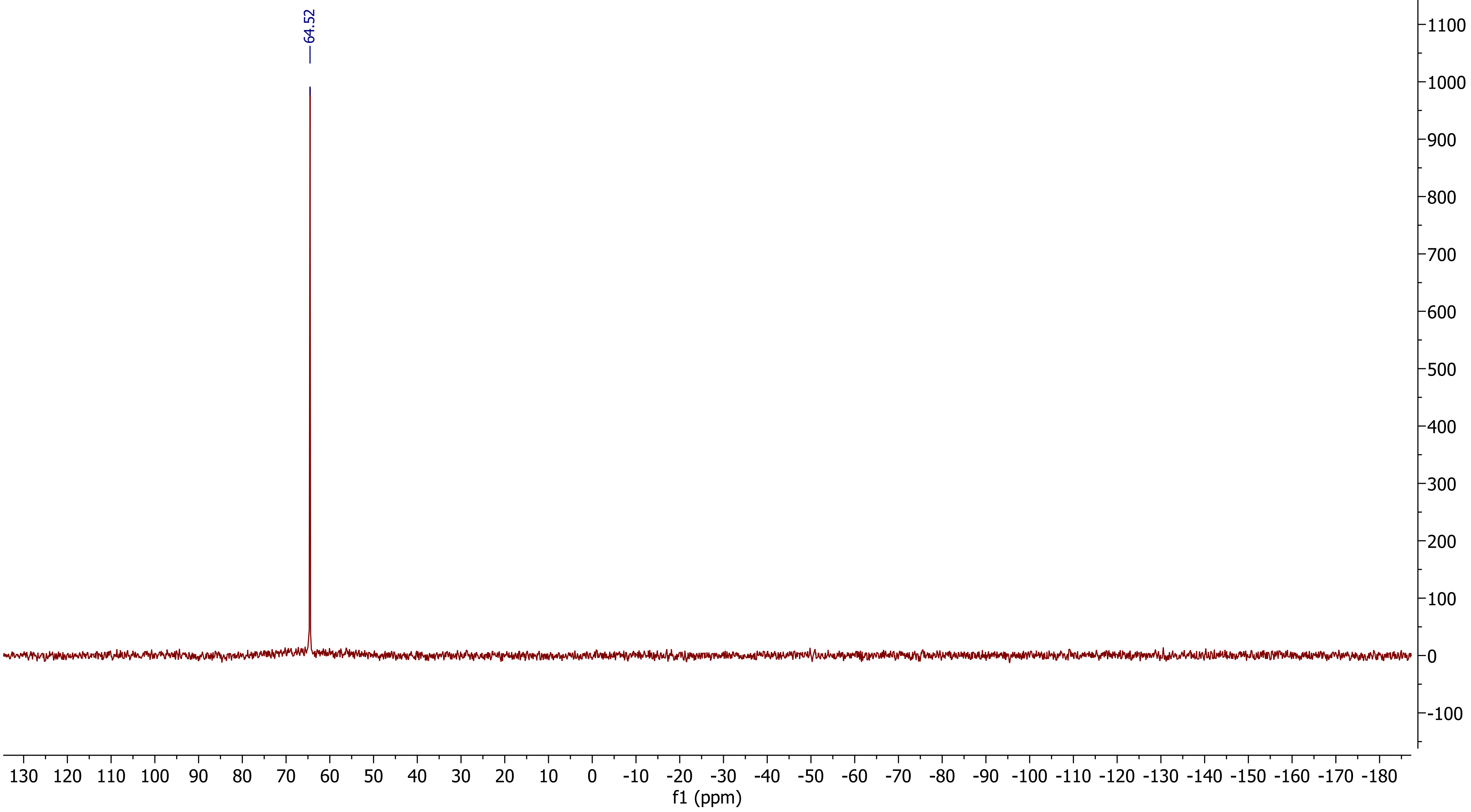

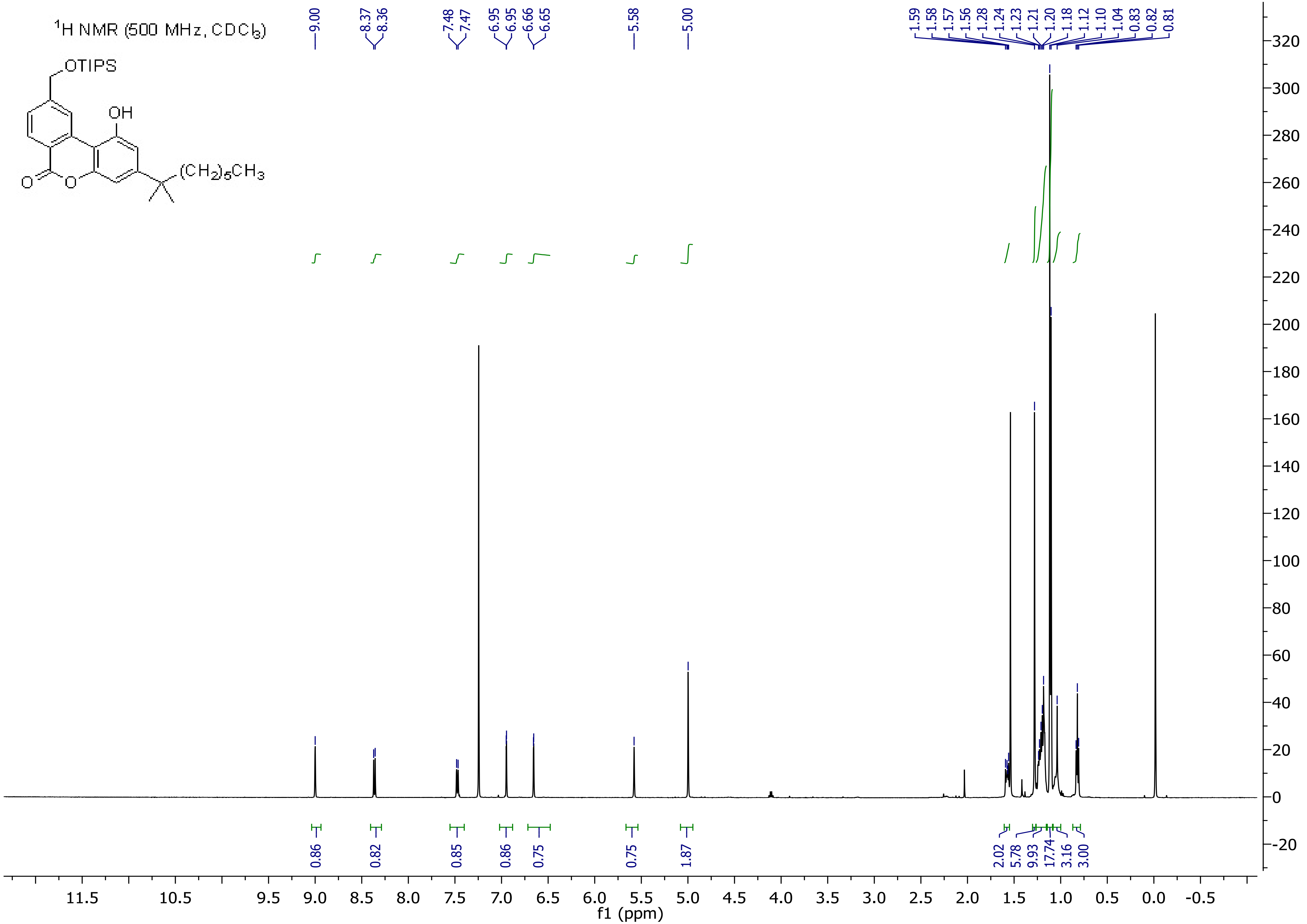

^{1}H NMR (500 MHz, CDCl$_3$)
OTIPS
OH
(CH$_2$)$_5$CH$_3$
9.00
8.37
8.36
7.48
7.47
6.95
6.95
6.66
6.65
5.58
5.00
1.59
1.58
1.57
1.56
1.28
1.24
1.23
1.21
1.20
1.18
1.12
1.10
1.04
0.83
0.82
0.81
0.86
0.82
0.85
0.86
0.75
0.75
1.87
2.02
5.78
9.93
17.74
3.16
3.00
f1 (ppm)

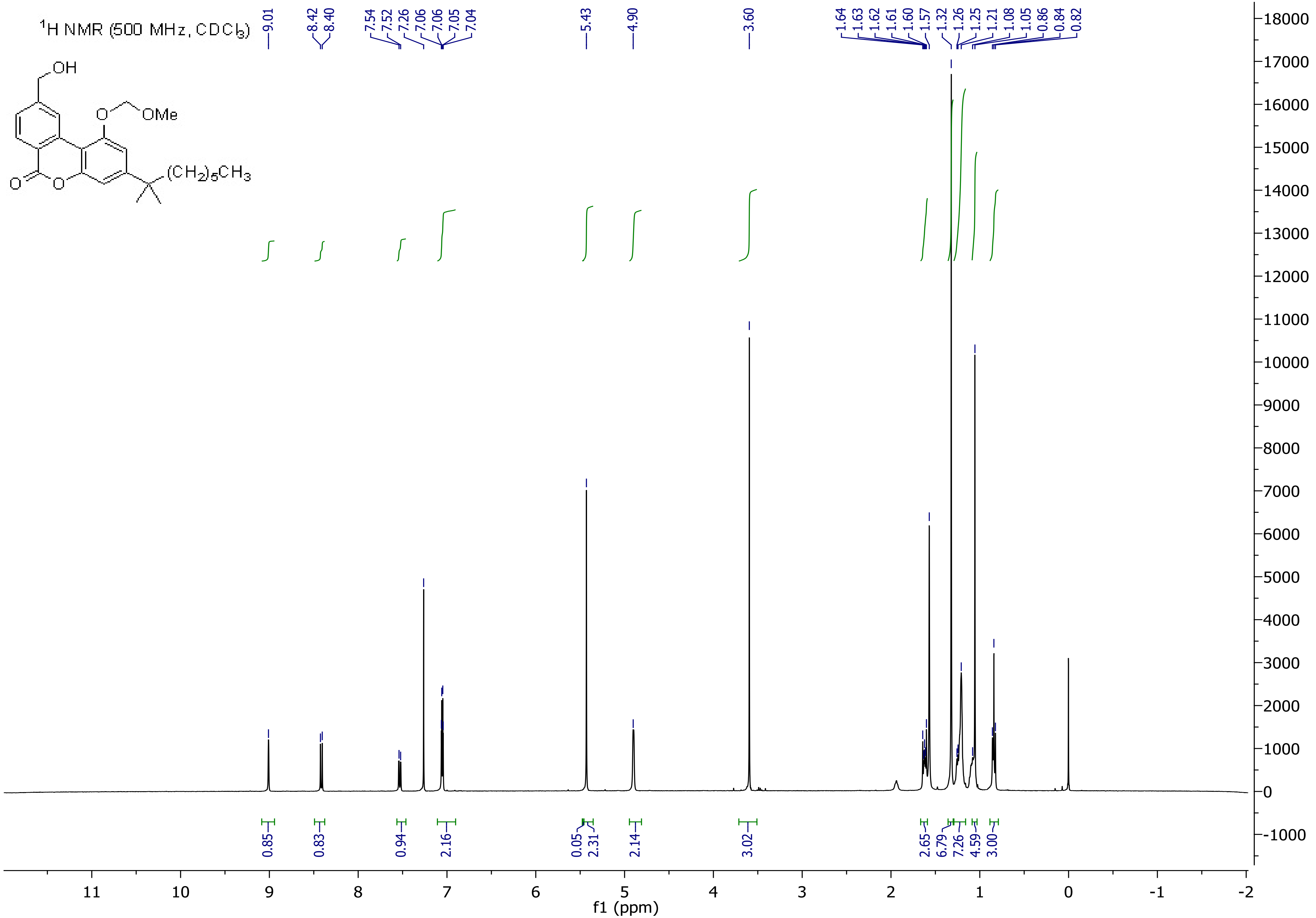

^{1}H NMR (500 MHz, CDCl3)
OH
OMe
O
(CH2)5CH3
9.01
8.42
8.40
7.54
7.52
7.26
7.06
7.06
7.05
7.04
5.43
4.90
3.60
1.64
1.63
1.62
1.61
1.60
1.57
1.32
1.26
1.25
1.21
1.08
1.05
0.86
0.84
0.82
0.85
0.83
0.94
2.16
0.05
2.31
2.14
3.02
2.65
6.79
7.26
4.59
3.00
f1 (ppm)

^1H NMR (500 MHz, CD_3OD)

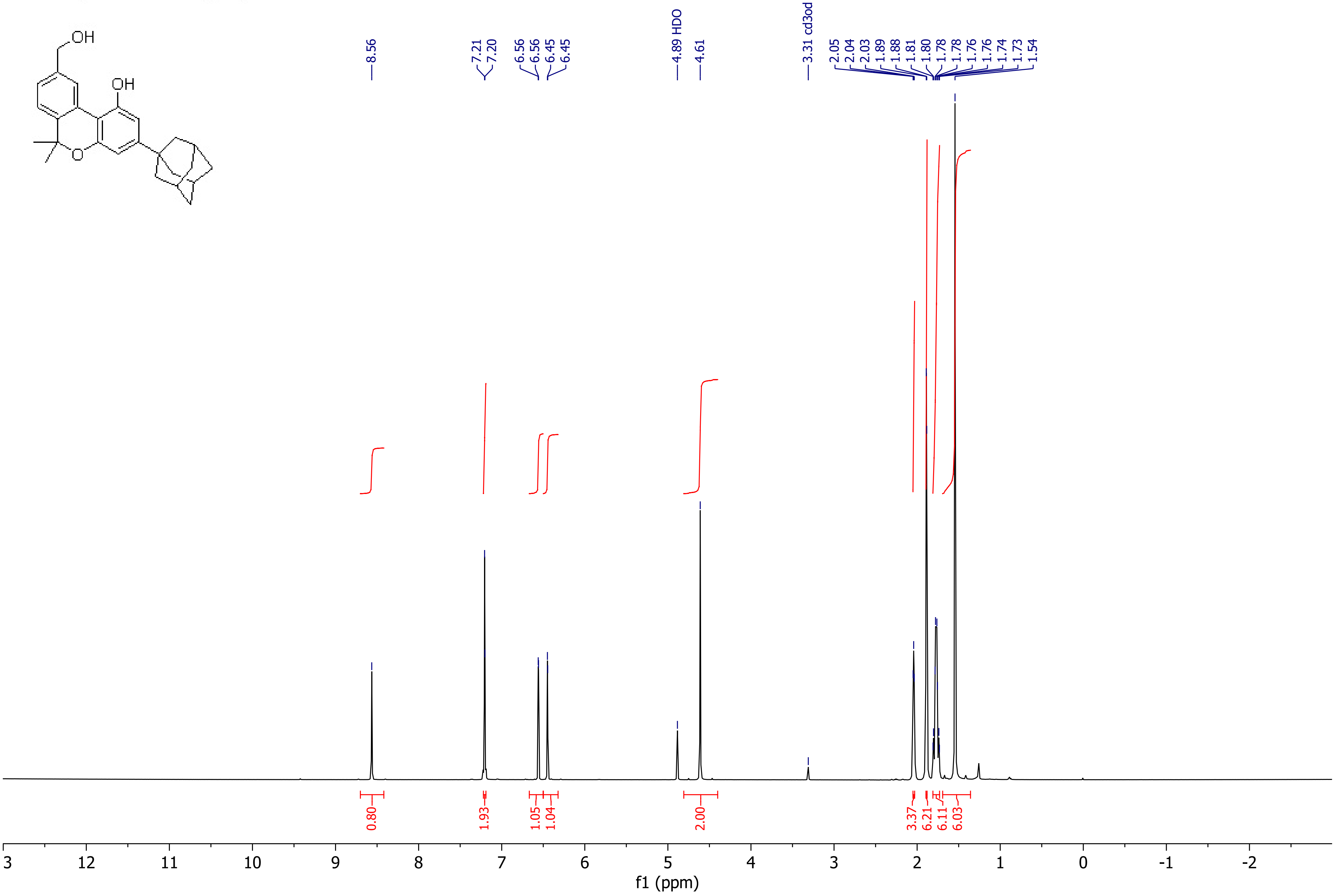

^{13}C NMR (126 MHz, CD_3OD)

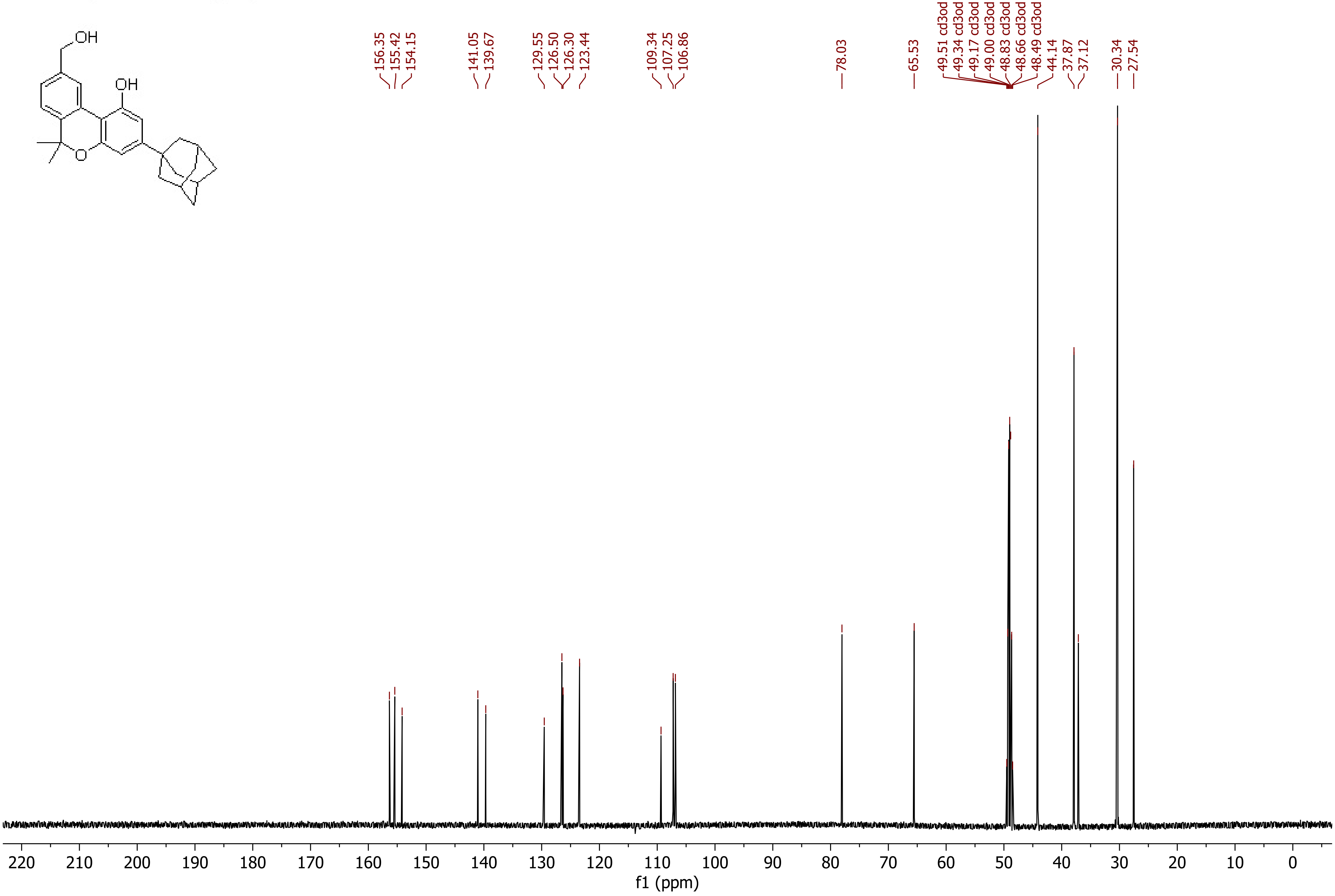

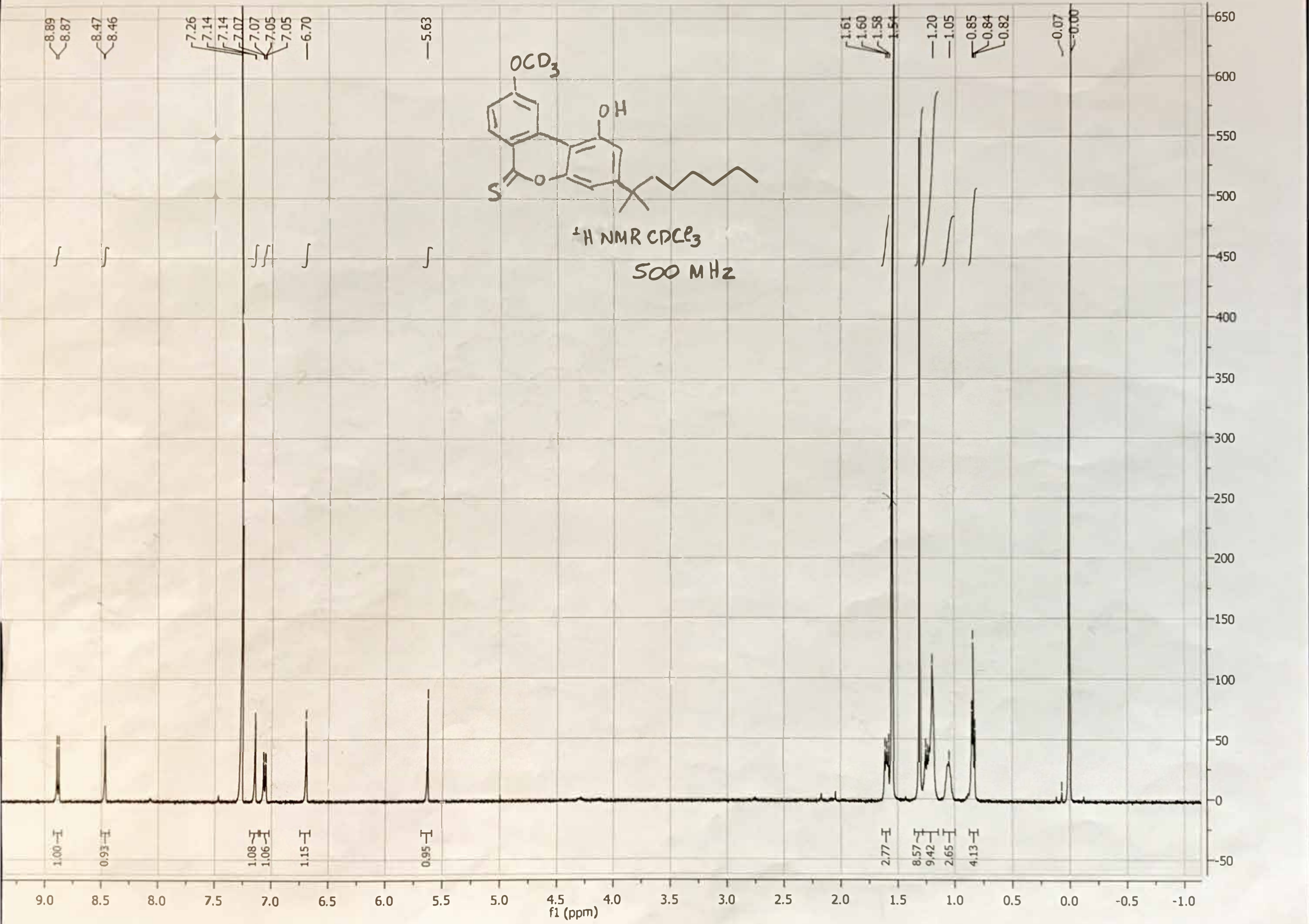
OCD3
OH
S
O
1H NMR CDCl3
500 MHz
f1 (ppm)
